# Identifying a Csmd3^+^ Microglial Subpopulation that Drives Cold‐to‐Hot Transition and Immune‐Cure in Glioblastoma

**DOI:** 10.1002/advs.76690

**Published:** 2026-07-20

**Authors:** Hai‐Feng Jiang, Pan‐Pan Gao, Yu‐Wen Du, Li‐Qin Wu, En‐Zhi Yin, Qi An, Ze‐Hua Ding, Jin‐Wen Shi, Ya Shu, Ruoqiao Chen, Feng Liu, Mingfeng Li, Xiao Qian Chen

**Affiliations:** ^1^ Department of Pathophysiology School of Basic Medicine Tongji Medical College Key Laboratory of Neurological Diseases Ministry of Education Hubei Provincial Key Laboratory of Neurological Diseases Huazhong University of Science and Technology Wuhan China; ^2^ Department of Pharmacy The First Affiliated Hospital of Yangtze University Jingzhou China; ^3^ Trauma Orthopedics Foot and Ankle Surgery Sun Yat‐Sen Memorial Hospital Sun Yat‐Sen University Guangzhou Guangdong People's Republic of China; ^4^ Department of Pharmacology and Toxicology Michigan State University East Lansing Michigan USA; ^5^ Department of Pharmacology School of Basic Medicine Tongji Medical College Huazhong University of Science and Technology Wuhan China; ^6^ The Key Laboratory for Drug Target Researches and Pharmacodynamic Evaluation of Hubei Province Wuhan China; ^7^ Innovation center for Brain Medical Sciences Tongji Medical College Huazhong University of Science and Technology Wuhan China

**Keywords:** anti‐GBM immune memory, Csmd3^+^ microglia, glioblastoma, TME subtype, αPD‐1

## Abstract

Glioblastoma (GBM) is immunologically cold and responds poorly to immune‐based therapies owing to its highly heterogeneous and immunosuppressive tumor microenvironment (TME). However, strategies to achieve a cold‐to‐hot transition remain elusive, and suitable research models are still lacking. Here, TME profiling classifies our refractory G422^TN^‐GBM model as the TME^Med^ (heterogeneous immune populations, “cold”) subtype of human GBM, which can be shifted toward the TME^High^ (immune‐high, “hot”) subtype by inhibiting TGF‐β signaling. In the multi‐drug regimen, only αTGF‐β combining temozolomide chemoradiotherapy and αPD‐1 achieves immune‐cure (ICu, passing tumor rechallenge, 12.5%). ICu screening reveals a newly identified Csmd3^+^ microglial subset with innate immune memory potential, which likely initiates durable anti‐GBM immune memory and closely associates with effective GBM therapy and favorable prognosis. MG^OE•^ *^Csmd3^* ^, BV2^ (*Csmd3*‐overexpressed microglial BV2) elicit robust anti‐GBM effects and achieve a notably 100% tumor rechallenge success in G422^TN^‐GBM mice via promoting TME^Med^‐to‐TME^High^ remodeling. Taken together, our findings identify an immunologically cold TME^Med^ GBM mouse model and provide a proof‐of‐concept for microglia‐based TME reprogramming and cell therapy in GBM.

## Graphical abstract

The study establishes an immune‐cure (ICu) mouse model based on a TME^Med^ G422^TN^‐GBM system that faithfully recapitulates human TME^Med^ GBM. scRNA‐seq analysis reveals a Csmd3^+^ microglial subset with innate immune memory (IIM) potential that potently suppresses GBM growth, drives a TME cold‐to‐hot transition, and induces 100% ICu in long‐term survival (LTS) mice.

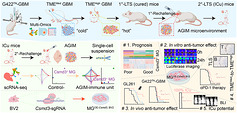

## 1 Introduction

Glioblastoma (GBM) is the most lethal primary brain tumor in adults, with a median overall survival (mOS) of approximately 15 months despite standard‐of‐care, i.e., maximal surgical resection plus temozolomide chemoradiotherapy (RT/TMZ) [1, 2, 3]. Despite extensive efforts in exploring novel therapeutic strategies, including immune checkpoint blockade (ICB) therapies such as anti‐PD‐1 (αPD‐1) that have transformed the treatment of many cancers [4, 5], GBM remains largely refractory due to its profoundly immunosuppressive tumor microenvironment (TME), extensive intra‐/inter‐tumoral heterogeneity, and rapid recurrence driven by high invasiveness [6, 7]. Effectively reprogramming the TME to prolong survival and prevent recurrence in GBM patients remains a pressing scientific and clinical challenge [8]. Lots of newly identified therapeutic targets/drugs are highly effective in conventional GBM preclinical models but fail to extend overall survival in clinical trials, underscoring the urgent need for a more stringent or refractory preclinical model for searching truly effective targets and validation [9, 10].

Recently, we have developed a much more refractory G422^TN^‐GBM model than other preclinical GBM models (such as GL261 and CT‐2A) and verified this by various therapeutic studies (e.g., RT/TMZ plus metformin or disulfiram) [11, 12]. Notably, the commonly used GL261 or CT‐2A models respond well to αPD‐1 therapy, even achieving long‐term survival (LTS, i.e., cure) [13, 14]; such a high ICB‐sensitivity has led to questions about their suitability for immunotherapy studies [15], while the G422^TN^‐GBM model has consistently failed to show survival benefit from αPD‐1 treatment [11, 12]. Further, GL261 and CT‐2A models achieve a 100% success in tumor rechallenge assay (2°‐LTS, i.e., immune‐cure, ICu) [16, 17, 18]; while the G422^TN^‐GBM model shows variable success rates (0%–50%) across different therapeutic regimens [11, 12], thus can effectively distinguish cured from immune‐cured effects, which is highly associated with the TME immune remodeling in GBM. Recently, GBM has been classified into immune TME subtypes, and predicting TME‐subtype transitions may better guide precision medicine, especially immunotherapy, than traditional molecular subtyping [19, 20].

**FIGURE 1:**
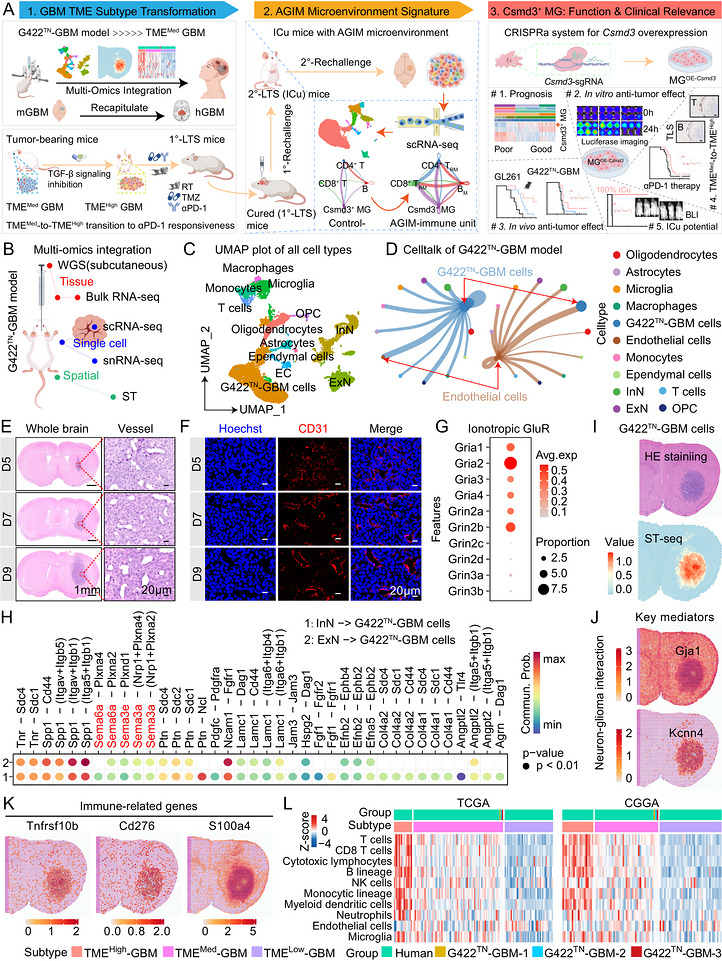
Characterizing TME subtypes in G422^TN^‐GBM compared with human GBM. (A) Experimental workflow (by Figdraw). TME, tumor microenvironment; AGIM, anti‐GBM immune memory; MG, microglia; TME^Med^ GBM, a human GBM subtype with heterogeneous immune cell populations and a “cold” TME; TME^High^ GBM, a human GBM subtype with immune‐high and a “hot” TME; mGBM, mouse GBM; hGBM, human GBM; LTS, long‐term survival; 2°‐LTS, second long‐term survival; 1°‐Rechallenge, first tumor rechallenge; 2°‐Rechallenge, second tumor rechallenge; RT, radiotherapy; TMZ, temozolomide; αPD‐1, anti‐PD‐1; ICu, immune‐cure; T_RM_, tissue‐resident memory T cells; B_M_, memory B cells; MG^OE•^ *^Csmd3^*, Csmd3 overexpression in microglia; TLS, tertiary lymphoid structures. (B) Schematic of integrated multi‐omics analysis in the G422^TN^‐GBM model. ST, spatial transcriptomics; WGS, whole‐genome sequencing. (C) Uniform manifold approximation and projection (UMAP) plot depicting all cell types in the integrated scRNA‐seq and snRNA‐seq dataset of the G422^TN^‐GBM model. OPC, oligodendrocyte progenitor cells; EC, endothelial cells; InN, inhibitory neurons; ExN, excitatory neurons. (D) Network diagram illustrating cell–cell communications between G422^TN^‐GBM cells and other microenvironmental cell types, showing that interactions with endothelial cells are the most abundant. (E‐F) H&E staining (E) and CD31 IF staining (F) showing angiogenesis in G422^TN^‐GBM tumors at days 5, 7, and 9 *p.i*. (G) Dot plots showing the expression of ionotropic glutamatergic receptors (GluRs) in G422^TN^‐GBM cells. Data are plotted as log‐normalized counts, and the dot size represents the proportion of cells with the given gene detected. Avg. exp., average expression. (H) Bubble plot showing significant ligand‐receptor pairs mediating communication from inhibitory neurons (InN, 1) and excitatory neurons (ExN, 2) to G422^TN^‐GBM cells. The size of each bubble represents the significance of the interaction (*p*‐value < 0.01), and the color intensity indicates the communication probability (from min to max). (I) H&E staining and spatial transcriptomics deconvolution jointly showing the localization of G422^TN^‐GBM cells. (J,K) Spatial transcriptomics surface plots showing regional expression of neuron–glioma interaction mediators (Gja, Kcnn4) in (J) and immune‐related genes (Tnfrsf10b, Cd276, S100a4) in (K) within the G422^TN^‐GBM model. (L) Heatmap illustrating three TME subtypes of GBM identified by partition around medoids (PAM) clustering of G422^TN^‐GBM model samples from three independent experiments (n = 14) with either the TCGA‐GBM (n = 126) or CGGA‐GBM (n = 98) cohorts. For each panel, RNA‐seq data from G422^TN^‐GBM and the corresponding cohort were batch‐corrected separately, after which expression profiles from each of the three independent G422^TN^‐GBM experiments were averaged separately. Clustering was based on the cellular TME composition quantified by GBM‐MCP‐counter scores.

In this study, multi‐omics analyses demonstrated that the G422^TN^‐GBM model faithfully recapitulates the TME^Med^ (heterogeneous immune populations, “cold”) subtype of human GBM, and that inhibition of TGF‐β signaling reprograms its TME into a TME^High^ (immune‐high, “hot”) subtype. Furthermore, combined blockade of TGF‐β and PD‐1 signaling plus RT/TMZ achieved only a few cures in the G422^TN^‐GBM model, with even fewer mice exhibiting ICu and durable anti‐GBM immune memory (AGIM). Durable AGIM is critical for preventing GBM recurrence [21], yet its cellular basis and mechanisms remain elusive. To address this, we established an ICu screening paradigm using ICu mice and single‐cell RNA‐sequencing (scRNA‐seq), revealing a significant enrichment of memory Csmd3^+^ microglia, tissue‐resident memory T (T_RM_) cells, and memory B (B_M_) cells (Figure 1A).

Microglia, the resident macrophages of the central nervous system (CNS), are increasingly recognized as pivotal regulators of the GBM's TME and display substantial heterogeneity in their plasticity and immune responsiveness according to specific microenvironmental cues [22]. Furthermore, microglia can acquire innate immune memory (IIM) through molecular reprogramming upon repeated stimulation [23, 24, 25], and newly developed microglial replacement technologies that replace senescent or impaired microglia with functional counterparts are providing novel therapeutic opportunities for brain disorders [26, 27, 28]. Recent studies increasingly emphasize that GBM immunity functions as a unit of multi‐cellular orchestration, including the microglia‐CD4^+^ T cell axis [29], the CD4^+^‐CD8^+^ T cell axis [30], intratumoral immune triads (APC‐CD4^+^‐CD8^+^) [31], and tertiary lymphoid structures (TLS) [32, 33]. Until now, the subtype‐specific function of microglia within the immune unit of GBM and their roles in treatment have not been reported.

Here, we discovered that memory Csmd3^+^ microglia cooperated with T_RM_ cells and B_M_ cells to form the AGIM unit, with Csmd3^+^ microglia likely serving as the primary initiators. We demonstrated that Csmd3^+^ microglia were closely associated with favorable prognosis, exhibited an antitumor phenotype, suppressed GBM progression, and promoted TME^High^ remodeling. Importantly, implantation of MG^OE•^ *^Csmd3^* ^, BV2^ cells (BV2 microglia with *Csmd3* overexpression) was capable of inducing a small subset of ICu mice in the TME^Med^ G422^TN^‐GBM model. Collectively, these findings underscore the translational value of the G422^TN^‐GBM model and identify Csmd3^+^ microglia with ICu potential as a promising therapeutic target in refractory GBM.

## 2 Results

### 2.1 G422^TN^‐GBM Recapitulates Well Human GBM TME^Med^ Features and is a Superior Model for Elucidating TME Subtype Dynamics

Recent GBM‐TME subtyping (TME^Low^, TME^Med^ and TME^High^), integrating features such as αPD‐1 responsiveness, immune cell infiltration, immune molecule expression, cell–cell interactions, and gene mutations (Table S1), is more effective than previous molecular subtypes of GBM in guiding precise immunotherapy strategies [19]. Until now, the TME subtyping of mouse GBM models has not been reported. Therefore, we first defined which specific human GBM TME subtype is recapitulated by the commonly used GL261‐GBM model and our G422^TN^‐GBM model. In the most common preclinical GL261‐GBM model, αPD‐1 effectively prolongs mOS (with some mice achieving cure) (Figure S1A), consistent with other preclinical studies [13, 15], suggesting that the GL261‐GBM model represents the TME^High^ GBM subtype. This is further reflected by the spontaneous formation of tertiary lymphoid structures (TLS, a hallmark of the TME^High^ GBM subtype [19] in the GL261‐GBM model (Figure S1B), as previously reported [32]. In the G422^TN^‐GBM model, αPD‐1 alone or combined with RT/TMZ failed to extend mOS (Figure S1C–I), highly consistent with our previous studies [11, 12] and a clinical trial [34]; and this model did not spontaneously form TLS (Figure S1J), suggesting that it represents the TME^Low^ or TME^Med^ GBM subtype. The differences in TME subtypes between these two models may be attributed to the fact that, under the same conditions, the G422^TN^‐GBM model exhibits higher malignancy compared with the GL261‐GBM model (as reflected by mOS, tumor burden, tissue density, and invasion index) and a colder immune microenvironment (as reflected by intratumoral T‐cell infiltration) (Figures S2 and S3).

Then, we comprehensively profiled the G422^TN^‐GBM TME subtype by integrating multi‐omics data including single‐cell RNA‐seq (scRNA‐seq), single‐nucleus RNA‐seq (snRNA‐seq), spatial transcriptomics, bulk RNA‐seq, and whole‐genome sequencing (WGS) on day 7 tumors (Figure 1B). scRNA‐seq and snRNA‐seq profiling identified the majority of cell types within the G422^TN^‐GBM TME, and revealed that G422^TN^‐GBM cells harbor at least four distinct meta‐programs (Figure 1C and Figure S4 and S5). Cell–cell communication analysis revealed that the strongest interactions were among G422^TN^‐GBM cells and endothelial cells, consistent with pronounced angiogenesis during early tumor progression at day 5–9 (Figure 1D–F and Figure S6). Neuron‐tumor interactions were also prominent, with G422^TN^‐GBM cells expressing most neurotransmitter receptors reported in human GBM, including glutamatergic, GABAergic, cholinergic, serotonergic, adrenergic, and dopaminergic receptors (Figure 1G and Figure S7A–H) [35, 36], along with additional neuronal signaling receptors such as members of the Semaphorin (Sema) receptor family (Figure 1H and Figure S7I) [37, 38]. Spatial deconvolution integrated with sc/snRNA‐seq further resolved cellular composition and dominant cell types at each tissue spatial spot (Figure 1I and Figure S8A), highlighting enrichment of well‐known neuronal‐glioma interaction mediators within the tumor, including Gja1 (connexin 43), KCa3.1 (Kcnn4), and Dlg4 (PSD95) (Figure 1J and Figure S8B) [39, 40]. G422^TN^‐GBM signature genes, derived from spatial and single‐cell data, were significantly enriched in pathways associated with neuronal proliferation, apoptosis, and differentiation (Figures S7J and S8C). Furthermore, different GBM TME subtypes exhibit distinct high‐frequency mutations [19]—Muc16 in TME^High^, Ttn and Trp53 in TME^Med^, and Egfr in TME^Low^—while WGS of G422^TN^‐GBM revealed Ttn and Trp53 mutations (missense or frameshift deletions) but no Muc16 or Egfr alterations (Tables S1 and S2). These data revealed the high endothelial cell characteristics, enrichment of neuronal signaling pathways, and distinct gene mutations in the G422^TN^‐GBM model, supporting its alignment with the TME^Med^ subtype of human GBM.

Further profiling immune genes including cytokines (Ccl25, Tnfrsf10b, Ifngr1), checkpoints (Cd276, Lgals9), and immune cell markers (Tmem119, Mrc1/CD206, Hmox1, S100a4) showed that they were evidently upregulated in tumor regions versus adjacent brain tissue, arguing against a TME^Low^ subtype (Figure 1K and Figure S8D–F). Importantly, integration of three independent G422^TN^‐GBM bulk RNA‐seq datasets with TCGA and CGGA cohorts matched G422^TN^‐GBM TME to human GBM‐TME^Med^ subtype (Figure 1L and Figure S9). Consistently, in the widely recognized tumor immune microenvironment (TIME) classification system for peripheral cancers (Table S1) [19, 20, 41], the G422^TN^‐GBM TME shared considerable similarity to the TIME^Exc^ subtype (immune‐excluded, “cold”) (Figure S9). Taken together, we defined G422^TN^‐GBM TME to be the TME^Med^ subtype, which is an ideal model for investigating the spatiotemporal dynamics of GBM‐TME evolution (from “cold” to “hot”).

### 2.2 TGF‐β Signaling Inhibition Induces “cold‐to‐hot” Transition/TME Remodeling in G422^TN^‐GBM Model and Immune‐Cure After Combining RT/TMZ and αPD‐1

TGF‐β signaling is a key driver of tumor progression, invasion, immune evasion, and therapeutic resistance [42, 43], whereas its inhibition can enhance tumor sensitivity to ICB by alleviating immunosuppression and reprogramming the TME from “cold” to “hot” [44, 45]. Therefore, we tested whether TGF‐β signaling inhibition can induce “cold‐to‐hot” transition and TME remodeling in the TME^Med^ G422^TN^‐GBM model. First, we calculated ssGSEA‐based pathway scores from MSigDB signatures (Table S3) and found that higher scores were consistently associated with poor prognosis across multiple bulk RNA‐seq datasets (TCGA, TCGA‐array, CGGA, GSE16011) (Figure S10). Then, we examined the spatial dynamics of TGF‐β signaling in human GBM using the Ivy‐GAP transcriptomic atlas (Ivy‐hGBM cohort) [46] and found that TGF‐β signature scores were markedly lower at the tumor leading edge and infiltrating regions compared with the cellular tumor core (TC) (Figure S11A), a result that was consistently observed in our TME^Med^ G422^TN^‐GBM model (Figure S11B,C). These findings indicate that TGF‐β pathway activity is largely restricted to the TC in GBM and highlight the potential importance of TGF‐β signaling in regulating the GBM TME.

**FIGURE 2:**
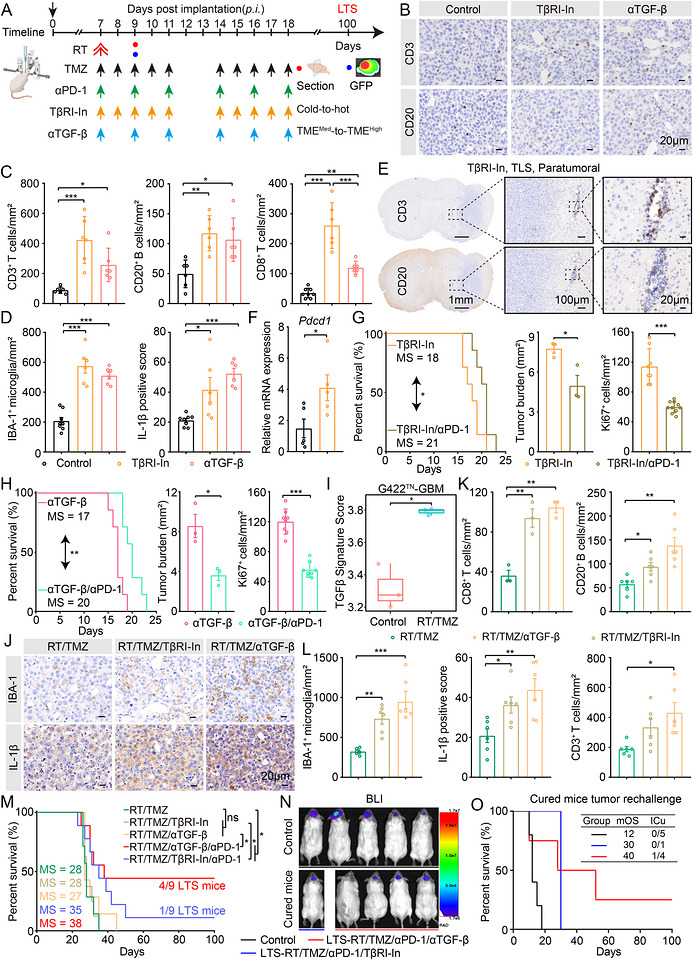
TGF‐β blockade reprograms the G422^TN^‐GBM TME and induces immune‐cure with RT/TMZ and αPD‐1. (A) Schematic diagram depicting the treatment regimens with RT, TMZ, αPD‐1, TβRI‐In, αTGF‐β, or their combinations initiated on day 7 *p.i*., with GFP imaging performed on day 9. RT, a single dose of 10 Gy whole‐brain irradiation (WBI); TMZ, 10 doses of temozolomide (50 mg/kg, ig); αPD‐1, six doses (ip; 400 µg/mouse for the first dose, followed by 200 µg/mouse); TβRI‐In, 50 mg/kg, ig, twice daily; αTGF‐β, six doses (ip; 200 µg/mouse). (B) Representative IHC staining of CD3 and CD20 in G422^TN^‐GBM tumors from Control, TβRI‐In, and αTGF‐β groups on day 9 *p.i*. (C,D) Statistical analysis of IHC staining for CD3, CD20, CD8, IBA‐1, and IL‐1β in G422^TN^‐GBM tumors from Control, TβRI‐In, and αTGF‐β groups (n = 6–8/group). (E) Representative images of peritumoral TLS in the TβRI‐In group detected by sequential whole‐brain sections with IHC staining of CD3 and CD20. (F) RT‐qPCR analysis showing elevated *Pdcd1* mRNA levels in TβRI‐In‐treated G422^TN^‐GBM tumors compared with the control group (n = 5/group). (G,H) Survival analysis (left panel, n = 8/group), together with statistical analyses of tumor burden (middle panel, n = 3/group) and KI67 IHC staining (right panel, n = 8–10/group), collectively demonstrated that blockade of TGF‐β signaling by TβRI‐In (G) or αTGF‐β (H) triggered αPD‐1 responsiveness in G422^TN^‐GBM tumors. (I) Boxplots showing estimated TGF‐β signature scores across different groups (RT/TMZ vs. Control) in G422^TN^‐GBM tumors (n = 3/group). (J) Representative IHC staining of IBA‐1 and IL‐1β in G422^TN^‐GBM tumors from RT/RMZ, RT/TMZ/TβRI‐In, RT/TMZ/αTGF‐β groups on day 9 *p.i*. (K‐L) Statistical analysis of IHC staining for IBA‐1, IL‐1β, CD8 (n = 3/group), CD20, and CD3 in G422^TN^‐GBM tumors from RT/RMZ, RT/TMZ/TβRI‐In, RT/TMZ/αTGF‐β groups (n = 6/group). (M) Survival curve of the G422^TN^‐GBM mice with RT/RMZ, RT/TMZ/TβRI‐In, RT/TMZ/αTGF‐β, RT/TMZ/TβRI‐In/αPD‐1 and RT/TMZ/αTGF‐β/αPD‐1 treatment (n  = 8–9/group). LTS, long‐term survival (>100 d, i.e., cure). (N) BLI showing intracranial tumors at day 7 after tumor rechallenge in cured mice treated with RT/TMZ/TβRI‐In/αPD‐1 (n = 1) and RT/TMZ/αTGF‐β/αPD‐1 (n = 4), with the control group (n = 5) serving as age‐ and sex‐matched controls. BLI, bioluminescence images. (O) Survival curves and LTS outcomes of cured mice from RT/TMZ/TβRI‐In/αPD‐1 (n = 1) and RT/TMZ/αTGF‐β/αPD‐1 (n = 4) groups after tumor rechallenge, with the control group (n = 5) serving as age‐ and sex‐matched controls. mOS, median overall survival. Statistical analysis: one‐way ANOVA followed by Tukey's post hoc test (C, D, K and L), two‐tailed unpaired Student's t test (F, G, H, and I). Survival curves were analyzed using a log‐rank (Mantel‐Cox) test (G, H and M). Error bars, mean ± SEM. ^∗^ *p* < 0.05; ^∗∗^ *p* < 0.01; ^∗∗∗^ *p* < 0.001.

To evaluate the impact of TGF‐β signaling inhibition on GBM TME subtype transition, we pharmacologically blocked the pathway using a TGF‐β receptor I inhibitor (TβRI‐In, Galunisertib) or a neutralizing anti‐TGF‐β antibody (αTGF‐β) in our TME^Med^ G422^TN^‐GBM model (Figure 2A and Figure S12A). First, we found that inhibition of TGF‐β signaling significantly prolonged mOS of mice when treatment was initiated on day 3 post‐implantation (p.i.), but showed no benefit when treatment was started on day 5 or day 7 p.i. (Figure S12). Nevertheless, blockade of TGF‐β signaling initiated at day 7 increased intratumoral infiltration of microglia (IBA1^+^), T cells (CD3^+^, CD8^+^), and B cells (CD20^+^), as well as the expression of interleukin‐1β (IL‐1β), a cytokine associated with M1‐like macrophages (Figure 2B–D and Figures S13, S14A); promoted the formation of TLSs in some, but not all, treated mice (Figure 2E and Figure S14B); and enhanced the upregulation of key TME^High^‐associated genes, including *Pdcd1, Ctla4, Cd274, Il1b, P2ry12, Il2, Il12a, Il23a*, and *Ccl5* (Figure 2F and Figure S15). Spatial analysis revealed the pro‐angiogenic role of TGF‐β signaling (Figure S11A), which was further supported by the observation that its blockade markedly suppressed angiogenesis in TME^Med^ G422^TN^‐GBM tumors (Figure S14C,D). Importantly, TGF‐β inhibition triggered a response to αPD‐1 therapy in TME^Med^ G422^TN^‐GBM tumors (Figure 2G,H and Figure S12E), and dual blockade achieved synergistic tumor suppression, as confirmed by GFP imaging (Figure S16). Collectively, while TGF‐β inhibition alone exerts limited therapeutic efficacy in GBM, it reprograms the TME from TME^Med^ to TME^High^, thereby promoting the conversion of GBM from a “cold” tumor to a “hot” tumor and creating a permissive landscape for effective αPD‐1 therapy.

Furthermore, analysis of TCGA and CGGA cohorts revealed that TGF‐β signaling is upregulated in glioma patients following RT/TMZ treatment, correlating with shorter survival (Figure S17). Similarly, our bulk RNA‐seq dataset [12] showed an increased TGF‐β signature score in the TME^Med^ G422^TN^‐GBM model after RT/TMZ (Figure 2I), suggesting that TGF‐β signaling may be involved in TME remodeling during standard GBM therapy (RT/TMZ). To validate this hypothesis, we further used the TME^Med^ G422^TN^‐GBM model to inhibit TGF‐β signaling in combination with RT/TMZ treatment. Although TGF‐β blockade did not further enhance the therapeutic efficacy of RT/TMZ, we found that TGF‐β inhibition in RT/TMZ‐treated G422^TN^‐GBM tumors increased intratumoral infiltration of microglia (IBA1^+^), T cells (CD3^+^, CD8^+^), B cells (CD20^+^), and IL‐1β expression (Figure 2J–L and Figure S18). Notably, TGF‐β blockade rendered the RT/TMZ‐treated tumors responsive to αPD‐1, resulting in long‐term survival (LTS) in a subset of mice (Figure 2M and Figure S18A–C). These results indicate that, even under RT/TMZ treatment, TGF‐β inhibition can induce a shift of the G422^TN^‐GBM TME toward a TME^High^‐like state and trigger responsiveness to αPD‐1 therapy.

Tumor rechallenge in LTS mice is a key approach for evaluating the durability of antitumor immunity and the establishment of immune memory [16, 17, 18]. Subsequently, we conducted a tumor rechallenge assay using cured (i.e., LTS) mice obtained from RT/TMZ/αTGF‐β/αPD‐1 combined therapies (Figure 2M). In this model, 25% of the cured (i.e., LTS or 1°‐LTS) mice successfully passed tumor rechallenge (without therapy) and achieved the secondary LTS (i.e., 2°‐LTS or ICu), suggesting that the 2°‐LTS mice harbored a durable and functional AGIM microenvironment that can prevent GBM growth during tumor rechallenge (Figure 2N, O). Unlike widely used GL261 and CT‐2A models that nearly 100% of LTS mice can achieve 2°‐LTS across various treatments (Figure S19 and Table S4) [16, 17, 18], the TME^Med^ G422^TN^‐GBM model achieves 0%–50% 2°‐LTS from LTS mice in various combined therapies (Figure 2N,O). Thus, our model can effectively distinguish LTS mice that fail to establish AGIM from 2°‐LTS mice that generate durable AGIM, and our 2°‐LTS mice represent truly ICu from highly refractory GBM.

### 2.3 Cellular Composition of Durable AGIM Revealed by ICu Screening in the TME^Med^ G422^TN^‐GBM Model

**FIGURE 3:**
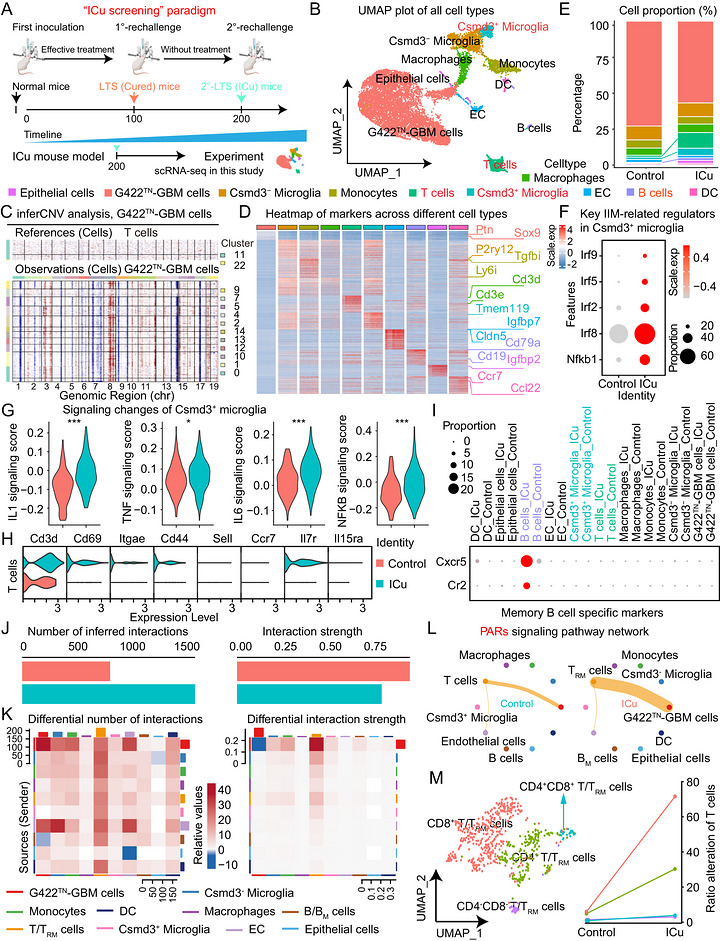
Generating ICu mice and scRNA‐seq analysis following 2°‐rechallenge. (A) Schematic of the “ICu screening” paradigm, in which cured mice achieve ICu upon tumor rechallenge, followed by 2°‐rechallenge and tumor tissue collection for scRNA‐seq to identify ICu‐associated cellular composition and mechanisms (by Figdraw). LTS, long‐term survival; ICu, immune‐cure, i.e., 2°‐LTS. (B) UMAP plot showing all cell types in the integrated scRNA‐seq data from ICu and control mice. DC, dendritic cells. (C) Hierarchical heatmap showing large‐scale CNV profile of each G422^TN^‐GBM cell cluster. Red and blue colors represent high and low CNV levels, respectively. T cells are defined as reference cells. CNV, copy number variation. (D) Heatmap of expression of the marker genes for each cell type from the integrated single‐cell data. Scale.exp, scaled expression. (E) Stacked barplot of cell composition of ICu and control mice from our scRNA‐seq dataset. (F) Dot plots showing the expression of key IIM regulators (Irf9, Irf8, Irf5, Irf2, and Nfkb1) in Csmd3^+^ microglia from ICu versus control mice. IIM, innate immune memory. (G) Violin plots showing changes in IL‐1, TNF, IL‐6, and NF‐κB signaling signature scores in Csmd3^+^ microglia from ICu and control mice. (H) Violin plots showing the expression of *Cd69*, *Itgae* (encoding CD103), *Cd44*, *Sell* (encoding CD62L), *Ccr7*, *Il7r*, and *Il15ra* in Cd3^+^ T cells from ICu and control mice. (I) Dot plots showing the expression of memory B cell markers (Cxcr5 and Cr2, encoding Cd21) across all cell types in ICu and control mice. (J) Bar plots showing the total number and strength of intercellular communications across all cell types in scRNA‐seq data from ICu and control mice. (K) Heatmap showing changes in the number and strength of interactions between different cell types across all cells in ICu mice compared with control mice. (L) Network diagram illustrating changes in intercellular interactions mediated by the PARs signaling pathway, primarily between T/T_RM_ cells and G422^TN^‐GBM cells. T_RM_, resident memory T cells; B_M_, memory B cells. (M) UMAP plot showing subclusters of T cells in control mice and T_RM_ cells in ICu mice (left panel); the right panel illustrates the trends of T cell subcluster changes between the two groups. Statistical analysis: two‐tailed unpaired Student's t‐test (G). ^∗^ *p* < 0.05; ^∗∗∗^ *p* < 0.001.

With these rare ICu mice, we performed the second tumor rechallenge (2°‐rechallenge) and scRNA‐seq on day 7 *p.i*. in order to search for cells or molecules underlying durable AGIM (called “ICu” screening) (Figure 3A). Leveraging the single‐cell dataset, integration, quality control, and cell annotation revealed that all cells could be classified into ten major lineages: G422^TN^‐GBM cells, monocytes, macrophages, endothelial cells (ECs), epithelial cells, B cells, dendritic cells (DCs), Csmd3^+^ microglia, Csmd3^−^ microglia, and T cells (Figure 3B–D and Figure S20). While the cell type compositions were largely similar between control and ICu mice, their relative abundances differed substantially. Notably, Csmd3^+^ microglia, T cells, and B cells were significantly enriched—showing more than a threefold increase—in the AGIM microenvironment of ICu mice (Figure 3E). Although Csmd3^+^ microglia were enriched in our study, their existence has not been validated previously [47, 48]. Double‐fluorescent immunostaining confirmed the existence of a few Csmd3^+^ microglia (IBA1^+^ CSMD3^+^) in the mouse cerebral cortex (Figure S21A). Consistently, immunofluorescence and Western blot analyses confirmed CSMD3 in both murine (BV2) and human (HMO6) microglial cell lines (Figure S21B,C). Furthermore, analysis of the rechallenged tumor in ICu mice (obtained from RT/TMZ/ACT001 combined therapies) [49] confirmed the increase of Csmd3^+^ microglia, T cells, and B cells within the durable AGIM microenvironment (Figure S22). Therefore, Csmd3^+^ microglia, T cells, and B cells may be key mediators of durable AGIM in preventing GBM recurrence.

Durable AGIM depends on memory immune cells; therefore, we examined the memory phenotypes of Csmd3^+^ microglia, T cells, and B cells. It is known that repeated exposure to the same stimulus can induce molecular reprogramming in microglia, resulting in either a trained innate immune memory (IIM) state, with enhanced pro‐inflammatory cytokine production, or a tolerant IIM state, with dampened inflammatory responses [23, 24, 25]. Comparing the Csmd3^+^ microglia subtype in 2°‐rechallenged tumor to that of naïve tumors, Csmd3^+^ microglia in ICu mice were transcriptionally primed for stronger cytokine response, including TNF‐α production (Figure S23) and IL‐1/TNF/IL‐6 pathway activation (Figure 3F,G and Table S3). Notably, transcription factors from the IRF and NF‐κB families—key regulators of IIM [23, 25, 50]—were significantly upregulated in Csmd3^+^ microglia from ICu mice (Figure 3F,G). Thus, Csmd3^+^ microglia subtype in the AGIM microenvironment of ICu mice underwent molecular reprogramming and acquired a distinct antitumor phenotype consistent with a training‐like IIM state.

T cells expressing CD69 alone, or co‐expressing CD69 and CD103 (encoded by *Itgae*), are widely recognized as tissue‐resident memory T (T_RM_) cells, which reside in non‐lymphoid tissues [51, 52]. Consistent with this, T cells in the AGIM microenvironment of ICu mice exhibited a T_RM_ phenotype, characterized by high Cd69, Itgae, and Il7r expression, and low Il15ra (encoding IL‐15R), Ccr7, and Sell (encoding CD62L) expression (Figure 3H). Notably, elevated Il7r and Cd44 expression indicates that T_RM_ cells within the AGIM microenvironment retain functional effector attributes while maintaining a stable memory phenotype (Figure 3H). Additionally, B cells within the AGIM microenvironment (memory B, B_M_) also exhibited elevated expression of memory‐associated markers, including *Cr2* (encoding CD21) and *Cxcr5* [53, 54] (Figure 3I). In summary, these findings suggest that within the AGIM microenvironment of ICu mice, Csmd3^+^ microglia, T_RM_ cells, and B_M_ cells are activated and expand upon repeated G422^TN^‐GBM cell stimulation, thereby contributing to anti‐tumor immunity.

### 2.4 Activated Tumor‐Killing State of the Durable AGIM Microenvironment in ICu Mice

The trained‐like IIM state of Csmd3^+^ microglia following 2°‐rechallenge suggests that the AGIM microenvironment in ICu mice is in an activated tumor‐killing state. Supporting this, cell–cell communication analysis revealed more interactions overall but reduced communication strength, primarily due to diminished G422^TN^‐GBM self‐interaction, indicating suppressed tumor growth (Figure 3J,K). Consistently, G422^TN^‐GBM cell abundance was lower in ICu mice than in controls (Figure 3E). Conversely, communications among Csmd3^+^ microglia, T_RM_ cells, B_M_ cells, and G422^TN^‐GBM cells were generally enhanced, with the greatest increase between T_RM_ cells and G422^TN^‐GBM cells (Figure 3J,K), including an evident increase/activation of PARs (Gamz‐Pard3, Gamz‐Fr2)/TRAIL/LCK signaling pathways within the AGIM microenvironment of ICu mice (Figure 3L and Figure S24A–C). This indicates that T_RM_ cells exhibit robust memory activation and tumor‐killing activity in this context [55, 56]. Notably, CD6, CD226, and TRAIL signaling—mediated via Cd6‐Alcam, Cd226‐Pvr, and Tnfsf10‐Tnfrsf10b receptor‐ligand pairs—constitute unique T_RM_–G422^TN^‐GBM interactions (Figure S24B), potentially underpinning the antitumor memory function of T_RM_ cells in ICu mice [57].

We further subdivided T/T_RM_ cells into CD4^+^, CD8^+^, double‐positive (DP, CD4^+^CD8^+^), and double‐negative (DN, CD4^−^CD8^−^) subsets, which followed potential differentiation trajectories and grouped into three major states represented by DP, CD4^+^, and CD8^+^ T/T_RM_ cells (Figure S25). Compared with control TME, the AGIM of ICu mice showed a marked enrichment of CD8^+^ T_RM_ cells, followed by expansion of CD4^+^ T_RM_ cells (Figure 3M). Notably, CD4^+^ T_RM_ cells displayed an inflammatory phenotype (CD69^hi^CD103^lo^) (Figure S24D) [58], indicating that both CD4^+^ and CD8^+^ T_RM_ subsets were activated and proliferative upon 2°‐rechallenge with G422^TN^‐GBM cells. Communication network analysis revealed that CD8^+^ T_RM_ cells acted as dominant signal senders and receivers, whereas CD4^+^ T_RM_ cells primarily served as signal senders, potentially regulating CD8^+^ T_RM_ activity (Figure S24E). Collectively, the AGIM microenvironment in ICu mice represents an activated tumor‐killing state of the GBM TME.

### 2.5 Csmd3^+^ Microglia Initiate the AGIM Program and Predict Favorable Outcome in Diffuse Glioma

**FIGURE 4:**
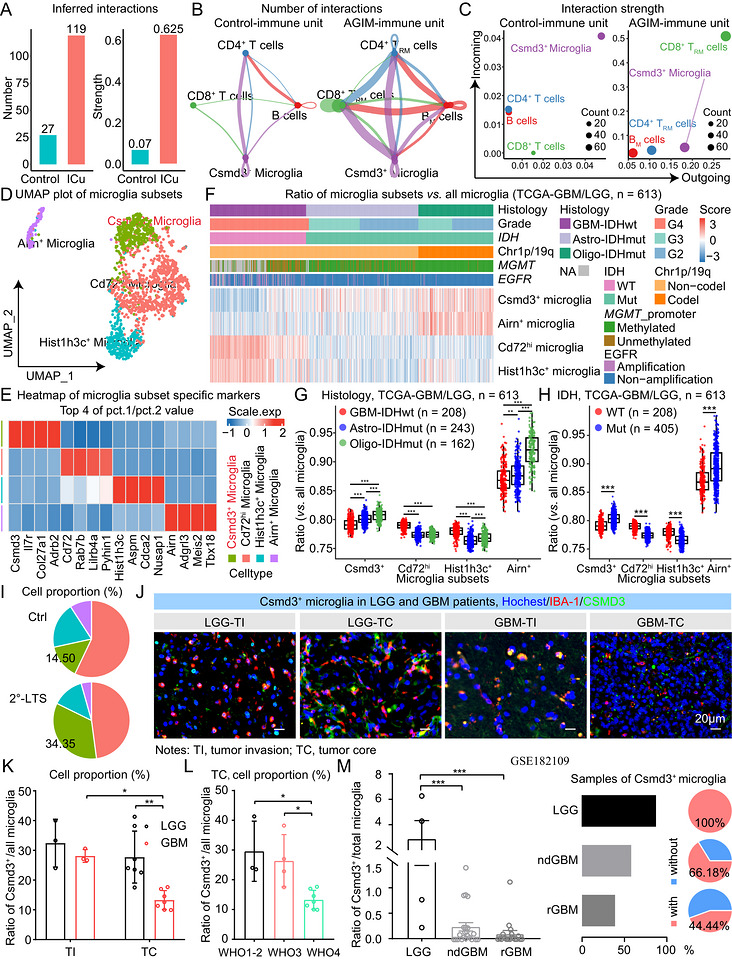
Csmd3^+^ microglia initiate anti‐GBM immune‐memory program and predict favorable outcome in diffuse glioma. (A) Bar plots showing the total number and strength of intercellular communications across microglia, B cells, and CD4^+^/CD8^+^ T cells in scRNA‐seq data from ICu and control mice. (B) Network diagram illustrating cell–cell communications of Control/AGIM‐immune units, including Csmd3^+^ microglia, B/B_M_ cells and CD4^+^/CD8^+^ T/T_RM_ cells. AGIM, anti‐GBM immune‐memory. (C) Scatter plot visualizing the major senders (sources) and receivers (targets) in Control/AGIM‐immune units in two‐dimensional space. (D) UMAP plot showing subclusters of microglia (Csmd3^+^, Airn^+^, Hist1h3c^+^ and Cd72^hi^) in the integrated scRNA‐seq data from ICu and control mice. (E) Heatmap showing the top 4 specific marker genes of microglial subclusters (Csmd3^+^, Airn^+^, Hist1h3c^+^ and Cd72^hi^) in our scRNA‐seq data, ranked by pct.1/pct.2 values. (F) Heatmap depicting the estimated proportions of different microglia subclusters across diffuse gliomas using the TCGA‐GBM/LGG cohort (n = 613 cases). LGG, low‐grade gliomas. Chr, chromosome. Codel, co‐deletion. NA, not applicable. WT, wild‐type; Mut, mutant. (G,H) Boxplots showing the estimated proportions of different microglia subclusters across diffuse gliomas with different types (G) or molecular signatures (H) using the TCGA‐GBM/LGG cohort (*n* = 613 cases). Center line shows median, box limits indicate upper and lower quartiles, and whiskers extend 1.5 times the interquartile range. (I) Pie charts illustrating the estimated proportions of different microglial subclusters in the integrated scRNA‐seq data from ICu and control mice. (J) Representative IF images of IBA1 and CSMD3 staining in the TI and TC regions of LGG and GBM patients. TI, tumor invasion; TC, tumor core. (K,L) Statistical analysis of the proportion of Csmd3^+^ microglia among all microglia in TI and TC regions of glioma patients, stratified by LGG and GBM (K), or by WHO grade I–IV (L). (M) Statistical analysis of the proportion of glioma patients harboring Csmd3^+^ microglia (right panel) and the proportion of Csmd3^+^ microglia among all microglia (left panel), stratified by LGG, ndGBM, and rGBM, based on public scRNA‐seq data from the GEO dataset GSE182109. ndGBM, newly diagnosed glioblastoma; rGBM, recurrent glioblastoma. Statistical analysis: two‐way ANOVA followed by Tukey's post hoc test (K), one‐way ANOVA followed by Tukey's post hoc test (G, L and M), two‐tailed unpaired Student's t test (H). Error bars, mean ± SEM. ^∗^ *p* < 0.05; ^∗∗^ *p* < 0.01; ^∗∗∗^ *p* < 0.001.

Growing evidence indicates that effective antitumor immunity depends on the coordinated activity of distinct immune cell subsets within the TME, forming functional immune units such as the microglia‐CD4^+^ T cell axis [29], the CD4^+^‐CD8^+^ T cell axis [30], intratumoral immune triads (antigen‐presenting cells‐CD4^+^‐CD8^+^ T cells) [31], and tertiary lymphoid structures (TLSs) [32, 33]. In line with this concept, the increased memory Csmd3^+^ microglia, B_M_ cells, CD4^+^ T_RM_ cells, and CD8^+^ T_RM_ cells in ICu mice (Figure 3) may collectively constitute a functional immune unit—the AGIM unit. Cell–cell communication analysis revealed markedly strengthened and frequent interactions among these memory cells in ICu mice (Figure 4A). Within this AGIM unit, newly established (de novo) intercellular regulatory interactions were observed, including reciprocal regulation between CD4^+^ and CD8^+^ T_RM_ cells, Csmd3^+^ microglia‐derived inputs into CD8^+^ T_RM_ cells, and B_M_ cell‐mediated modulation of both Csmd3^+^ microglia and CD8^+^ T_RM_ cells (Figure 4B). While CD8^+^ T cells are the primary cytotoxic effectors, simply enhancing their number or activity is insufficient to achieve durable antitumor responses [59, 60]. Instead, their coordinated regulation of CD8^+^ T_RM_ cells by Csmd3^+^ microglia, B_M_ cells, and CD4^+^ T_RM_ cells appears central to the establishment of the AGIM unit (Figure 4B). Signal flow analysis further revealed that within the AGIM unit, alongside CD8^+^ T_RM_ cells, Csmd3^+^ microglia act as key signal exporters. Notably, in control mice, Csmd3^+^ microglia displayed the highest levels of both signal inflow and outflow (Figure 4C). Combined with their known rapid responsiveness to brain pathology, these findings identify Csmd3^+^ microglia as frontline immune sentinels that initiate defense against GBM progression and orchestrate the AGIM unit.

To explore the relationship between microglial heterogeneity in the TME^Med^ G422^TN^‐GBM model and glioma prognosis, we re‐clustered microglia and identified four distinct subsets: Csmd3^+^, Cd72^hi^, Hist1h3c^+^, and Airn^+^ microglia (Figure 4D,E). Prognostic analyses revealed that the proportions of Csmd3^+^ and Airn^+^ microglia were decreased in IDHwt (IDH wild‐type) GBM compared with IDHmut (IDH mutant) gliomas, whereas Cd72^hi^ and Hist1h3c^+^ subsets were elevated (Figure 4F–H). These shifts also correlated with glioma grade and molecular features, including IDH mutation, 1p/19q codeletion, MGMT methylation, and EGFR amplification, across TCGA cohorts (Figure S26). Survival and Cox regression analyses confirmed these associations (Figure S27), which were consistently validated in CGGA, GSE16011, and TCGA‐array datasets (Figures S26 and S27). Together, these findings indicate that Csmd3^+^ and Airn^+^ microglia are linked to favorable prognosis, whereas Cd72^hi^ and Hist1h3c^+^ microglia predict poor outcomes. Notably, only Csmd3^+^ microglia were increased in ICu mice (Figure 4I), highlighting their unique role in shaping GBM TME.

We further examined Csmd3^+^ microglia in samples from patients with low‐grade gliomas (LGG) and GBM, and found that both the proportion of Csmd3^+^ microglia within tumors (tumor core, TC) progressively decreased with increasing glioma malignancy and grade (Figure 4J–L and Figure S28). Notably, the proportion of Csmd3^+^ microglia in the TC of GBM patients was lower than that in the tumor invasive (TI) region (Figure 4K). Moreover, in a public glioma scRNA‐seq dataset (GSE182109, 44 samples) [61], although some samples failed to detect Csmd3^+^ microglia due to sampling location or sequencing depth, the frequency of Csmd3^+^ microglia also decreased with increasing malignancy (Figure 4M and Figure S29). In summary, Csmd3^+^ microglia serve as a protective prognostic marker in diffuse glioma and may play a key role in reprogramming the GBM TME.

Additionally, analysis of microglial aging‐related scRNA‐seq datasets (GSE207948 and GSE226286) revealed that after three cycles of microglial ablation and regeneration using CSF1R inhibitor PLX5622 [62], the proportion of Csmd3^+^ microglia among total microglia declined with age in both flow cytometry‐isolated microglia (CD45^lo^CD11b^+^) and whole‐brain scRNA‐seq data (Figure S30). This age‐associated reduction may contribute to glioma susceptibility, linking older age to lower Csmd3^+^ microglia abundance and increased glioma risk.

### 2.6 Csmd3^+^ Microglia Predominantly Localize to the Tumor Periphery, But Effective Therapy Drives Their Infiltration Into the Tumor Core

**FIGURE 5:**
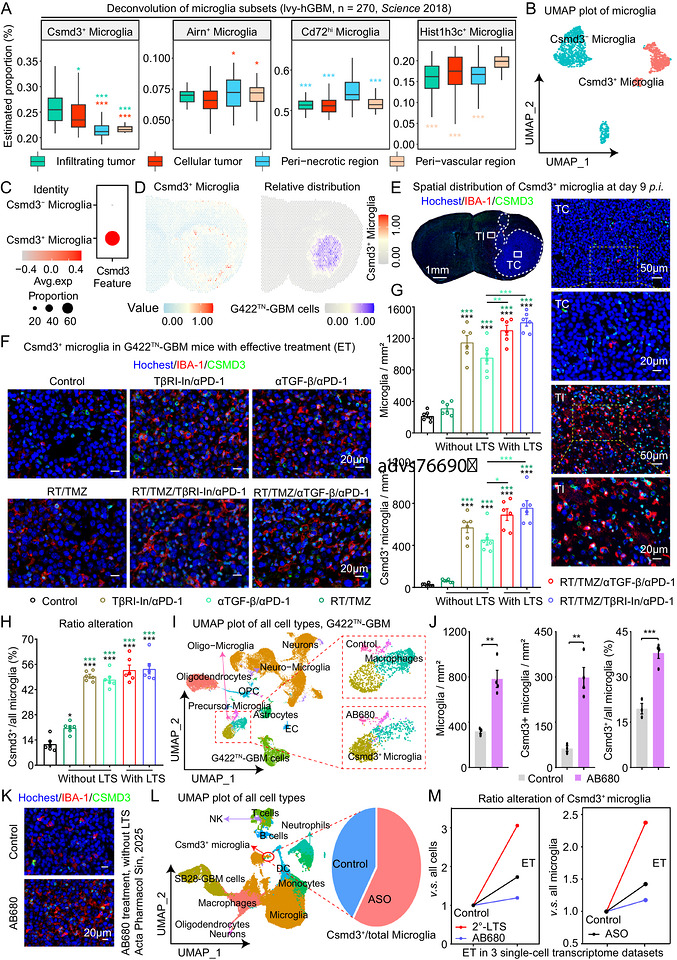
Csmd3^+^ microglia predominantly localize to the tumor periphery, but effective therapy drives their infiltration into the tumor core. (A) Boxplots showing the estimated proportions of different microglial subclusters of our scRNA‐seq data across different histological regions of hGBMs using bulk RNA‐seq data from the Ivy‐hGBM cohort (*n* = 270 cases). Estimated cell proportion of clusters with overhead asterisk differs from that of clusters with corresponding asterisk color. (B) UMAP plots depicting Csmd3^+^ microglia and Csmd3^−^ microglia in the integrated scRNA‐seq and snRNA‐seq dataset of the G422^TN^‐GBM model. (C) Dot plots showing the expression of Csmd3 in Csmd3^+^ microglia and Csmd3^−^ microglia. Data are plotted as log‐normalized counts, and the dot size represents the proportion of cells with the given gene detected. Avg. exp., average expression. (D) Surface plots showing the enrichment of Csmd3^+^ microglia and their spatial relationship with G422^TN^‐GBM cells, based on spatial transcriptomics deconvolution using integrated scRNA‐seq and snRNA‐seq data from the G422^TN^‐GBM model. (E) Representative IF images of IBA1 and CSMD3 staining in the TI and TC region of G422^TN^‐GBM mice. (F) Representative IF images of IBA1 and CSMD3 staining in the TC region of the G422^TN^‐GBM mice across TβRI‐In/αPD‐1, αTGF‐β/αPD‐1, RT/RMZ, RT/TMZ/TβRI‐In/αPD‐1, RT/TMZ/αTGF‐β/αPD‐1 treatment and control group on day 9 *p.i*. (G‐H) Statistical analysis of IF images showing microglia (IBA‐1^+^) (G, upper panel), Csmd3^+^ microglia (IBA‐1^+^CSMD3^+^) (G, lower panel), and the proportion of Csmd3^+^ microglia among all microglia (H) in G422^TN^‐GBM tumors on day 9 *p.i*. across TβRI‐In/αPD‐1, αTGF‐β/αPD‐1, RT/TMZ, RT/TMZ/TβRI‐In/αPD‐1, RT/TMZ/αTGF‐β/αPD‐1 treatment, and control groups (n = 6/group). Cured mice were generated only in the RT/TMZ/TβRI‐In/αPD‐1 and RT/TMZ/αTGF‐β/αPD‐1 groups. (I) UMAP plot showing all cell types in the integrated snRNA‐seq data from AB680‐treated and control G422^TN^‐GBM tumors. Oligo‐microglia, oligodendrocyte‐like microglia; Neuro‐microglia, Neuron‐like microglia. AB680, a small molecule inhibitor to target CD73. (J,K) Representative IF images of IBA1 and CSMD3 staining (K) and statistical analysis showing microglia (IBA‐1^+^) (J, left panel), Csmd3^+^ microglia (IBA‐1^+^CSMD3^+^) (J, median panel), and the proportion of Csmd3^+^ microglia among all microglia (J, right panel) in G422^TN^‐GBM tumors across AB680 treatment (n = 4/group) and control groups (n = 3/group). (L) UMAP plots showing all cell types (left panel) and pie charts illustrating the proportion of Csmd3^+^ microglia among all microglia in ASO‐treated and control SB28‐GBM tumors (right panel), based on public scRNA‐seq data from the GEO dataset GSE226292. NK, natural killer cells; ASO, antisense oligonucleotides to target Trem2. (M) Changes in the proportion of Csmd3^+^ microglia across integrated snRNA‐seq data from AB680‐treated mice (no cured mice), scRNA‐seq data from ASO‐treated mice (with cured mice), and scRNA‐seq data from ICu mice, showing the largest change in Csmd3^+^ microglia proportion in ICu mice and the smallest change in AB680‐treated mice. Statistical analysis: two‐sided unpaired Wilcoxon test (A), one‐way ANOVA followed by Tukey's post hoc test (G and H), two‐tailed unpaired Student's t test (J). Error bars, mean ± SEM. ^∗^ *p* < 0.05; ^∗∗^ *p* < 0.01; ^∗∗∗^ *p* < 0.001.

To map the spatial heterogeneity of microglial subsets (Csmd3^+^, Airn^+^, Cd72^hi^, and Hist1h3c^+^) in the TME^Med^ G422^TN^‐GBM model, we projected their transcriptional signatures onto distinct histological regions of human GBM using the Ivy‐Glioblastoma Atlas Project (Ivy‐GAP) dataset and estimated their relative abundances. The analysis revealed clear spatial preferences: Cd72^hi^ microglia were enriched in peri‐necrotic regions, Hist1h3c^+^ microglia localized primarily to perivascular areas, and Airn^+^ microglia were more diffusely distributed (Figure 5A). Notably, Csmd3^+^ microglia were most abundant in infiltrative tumor (tumor invasive, TI) regions, consistent with immunofluorescence results from glioma patient samples (Figures 4K and 5A). Furthermore, in the TME^Med^ G422^TN^‐GBM model, integration of scRNA‐seq, snRNA‐seq, and spatial transcriptomics data from day 7 of tumor progression also confirmed that Csmd3^+^ microglia predominantly occupy the tumor periphery (TI region) (Figure 5B–D). This spatial pattern was further validated by IBA1+CSMD3 staining, which revealed a significantly higher abundance of Csmd3^+^ microglia in the TI region compared to the TC at day 9 (Figure 5E and Figure S31). Collectively, these findings indicate that during glioma progression, Csmd3^+^ microglia fail to infiltrate the TC region, limiting their antitumor activity.

To assess Csmd3^+^ microglia infiltration during effective GBM therapy, we analyzed TME^Med^ G422^TN^‐GBM mice treated with various regimens, including RT/TMZ and combinations with αPD‐1/TGF‐β blockade. Effective regimens promoted overall microglial accumulation within tumors, increased the number of Csmd3^+^ microglia, and elevated their proportion among total intratumoral microglia (Figure 5F–H and Figure S32A). Notably, therapeutic groups that generated LTS mice exhibited markedly greater intratumoral infiltration of Csmd3^+^ microglia compared with effective but non‐LTS groups, a trend consistent with single‐cell transcriptomic data. Specifically, in our recently published snRNA‐seq datasets of the TME^Med^ G422^TN^‐GBM model (PRJNA1043813, PRJNA1251355) [63], treatment with the CD73 inhibitor AB680—an effective therapy but no LTS—resulted in an increased proportion of Csmd3^+^ microglia (Figure 5I and Figure S33). This elevation was further confirmed by IBA1+CSMD3 staining (Figure 5J,K and Figure S32B). Similarly, in a publicly available scRNA‐seq dataset (GSE226292) using the SB28‐GBM model [64], ASO‐mediated TREM2 inhibition increased the proportion of Csmd3 microglia and generated a subset of LTS mice (Figure 5L and Figure S34). Across these datasets, treatments producing LTS mice induced a more Csmd3^+^ microglia than non‐LTS therapies, with the most pronounced enrichment observed in ICu mice (Figure 5M), highlighting a strong association between Csmd3^+^ microglia accumulation and durable AGIM. Collectively, these results indicate that intratumoral enrichment of Csmd3^+^ microglia is a potential key driver of effective GBM therapy, contributing to LTS and AGIM‐mediated tumor eradication.

### 2.7 Csmd3^+^ Microglia Exhibit a Pro‐Inflammatory Phenotype that Significantly Suppresses GBM Progression

TGF‐β signaling, while essential for the development, maturation, and activation of microglia, may also drive them toward an anti‐inflammatory, tumor‐promoting phenotype [65, 66, 67]. We found that in vitro treatment of BV2 microglia with the TβRI‐In induced increased expression of the inflammatory factors Il1b, Tnf, and Il6, indicating their shift toward a pro‐inflammatory phenotype (Figure S35A,B). Moreover, during this process, we observed an increase in *Csmd3* expression (Figure S35B), which is consistent with the findings from the TCGA and CGGA datasets showing that *Csmd3* expression is negatively correlated with TGF‐β signaling (Figure S35C). Furthermore, in TMEM119‐CreER^T2^; Tgfbr2^fl/fl^ mice that conditionally delete TGF‐βR2 in microglia, we observed that the increase in Csmd3^+^ microglia infiltration into the TC region of G422^TN^‐GBM (Figure S35D–F). These findings suggest that TGF‐β signaling inhibition increases *Csmd3* expression or Csmd3^+^ microglia expansion, reprograms microglia toward a pro‐inflammatory phenotype, and promotes their infiltration to exert antitumor effects.

**FIGURE 6:**
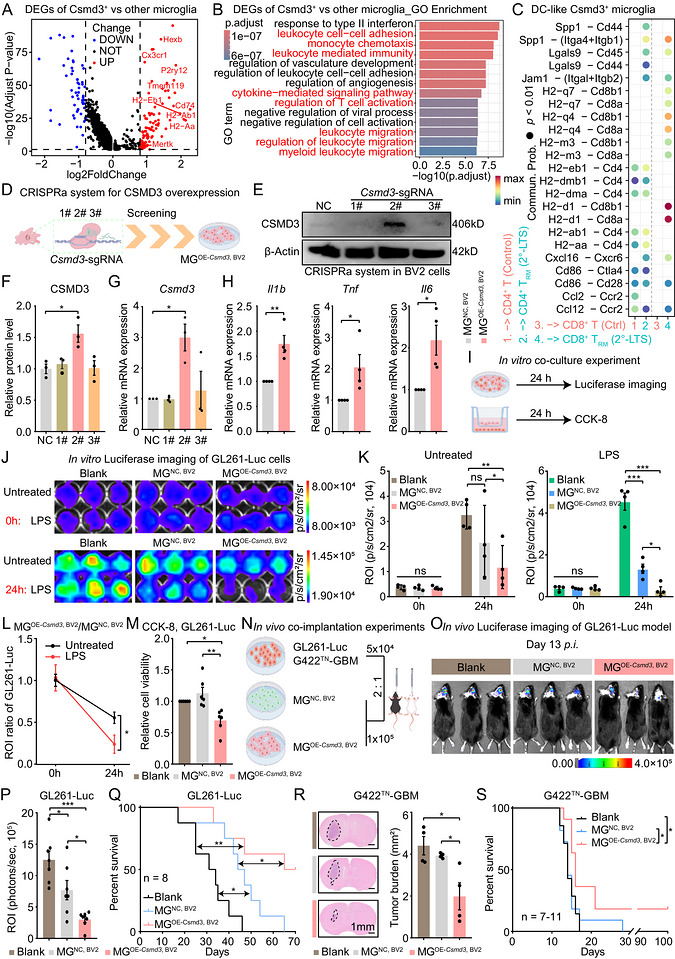
Csmd3^+^ microglia exhibit a pro‐inflammatory phenotype and suppress GBM progression. (A) Volcano plot showing DEGs in Csmd3^+^ microglia compared with other microglia in scRNA‐seq data from ICu and control mice. Upregulated genes are shown in red, including homeostatic microglia markers (Hexb, Cx3cr1, P2ry12, Tmem119), MHC‐II genes (H2‐Eb1, Cd74, H2‐Ab1, H2‐Aa), and phagocytic genes (Mertk); downregulated genes are shown in blue; genes with no significant change are shown in black. DEGs, differentially expressed genes. (B) Top 14 GO‐BP enrichment pathways of the upregulated genes in Csmd3^+^ microglia compared with other microglia in scRNA‐seq data from ICu and control mice identified by the GO pathway enrichment analysis. (C) Dot plot showing that ligand–receptor analysis indicates Csmd3^+^ microglia exhibit DC‐like features, capable of regulating CD4^+^ T cells via MHC‐I and CD8^+^ T cells via MHC‐II, with enhanced regulatory effects observed in ICu mice. (D) Schematic diagram of CRISPRa (CRISPR activation system) activation of endogenous Csmd3 overexpression in microglia (by Figdraw). Csmd3‐sgRNA 1#‐3#, three single guide RNAs of Csmd3; MG^OE‐Csmd3, BV2^, BV2 microglia overexpressing Csmd3. (E‐F) Representative Western blotting results (E) and statistical analysis (F) of CSMD3 protein in BV2 cells infected with Csmd3‐sgRNA #1–3 or its negative control (NC) (n = 3/group), with β‐Actin used as a loading control. Csmd3‐sgRNA #2 significantly induced CSMD3 overexpression. (G) RT‐qPCR analysis showing *Csmd3* mRNA levels in BV2 cells infected with Csmd3‐sgRNA #1–3 and control sequence (NC) (n = 3/group). (H) RT‐qPCR analysis showing elevated Il1b, Tnf, and Il6 in MG^OE‐Csmd3, BV2^ cells compared with MG^NC, BV2^ cells. MG^NC, BV2^, BV2 microglia infected with control (NC) (n = 4/group). (I) Schematic illustration of in vitro co‐culture experiments of MG^OE‐Csmd3, BV2^ cells, and MG^NC, BV2^ cells with GL261‐GBM cells, and subsequent functional assays on GL261‐GBM cells (by Figdraw). (J‐K) Representative in vitro luciferase images (J) of GL261‐GBM cells alone (blank) and direct co‐culture with MG^OE‐Csmd3, BV2^ or MG^NC, BV2^ cells, along with statistical analysis of ROI values (K) before (0 h) and after (24 h) co‐culture and with or without LPS treatment (n = 4/group). ROI, region of interest; LPS, lipopolysaccharide. (L) Direct co‐culture of MG^OE‐Csmd3, BV2^ cells or MG^NC, BV2^ cells with GL261‐GBM cells showing changes in the ROI ratio of MG^OE‐Csmd3, BV2^/MG^NC, BV2^ group before (untreated) and after LPS treatment (n = 4/group). (M) Statistical analysis of relative cell viability (CCK‐8 assay) of GL261‐GBM cells collected after indirect co‐culture with MG^OE‐Csmd3, BV2^ cells or MG^NC, BV2^ cells (*n*  =  6/group). (N) Schematic illustration of in vivo co‐implantation experiments of MG^OE‐Csmd3, BV2^ or MG^NC, BV2^ cells with GBM cells (GL261‐GBM or G422^TN^‐GBM) (by Figdraw). GBM cells were implanted at 5 × 10^4^ and microglia at 1 × 10^5^ (ratio 1:2). GL261‐Luc, GL261‐GBM cell overexpressing luciferase. (O,P) Bioluminescent images (O) and statistical analysis (P) of ROI values of intracranial tumors in the Blank (GL261‐GBM alone), GL261‐GBM + MG^NC, BV2^, and GL261‐GBM + MG^OE‐Csmd3, BV2^ groups on day 13 *p.i*. (n = 7/group). (Q) Survival curve of the GL261‐GBM‐bearing mice in the Blank (GL261‐GBM alone), GL261‐GBM + MG^NC, BV2^, and GL261‐GBM + MG^OE•^ *^Csmd3^* ^, BV2^ groups (n = 8/group). (R) H&E staining (left panel) and statistical analysis (right panel) of G422^TN^‐GBM tumor burden in the Blank (G422^TN^‐GBM alone), G422^TN^‐GBM + MG^NC, BV2^, and G422^TN^‐GBM + MG^OE‐Csmd3, BV2^ groups on day 7 *p.i*. (n = 8/group). (S) Survival curve of the G422^TN^‐GBM‐bearing mice in the Blank group (G422^TN^‐GBM alone, n = 7), G422^TN^‐GBM + MG^NC, BV2^ (n = 11), and G422^TN^‐GBM + MG^OE•^ *^Csmd3^* ^, BV2^ (n = 11) groups. Statistical analysis: one‐way ANOVA followed by Tukey's post hoc test (F, G, M, P and R), two‐tailed unpaired Student's t test (H), two‐way ANOVA followed by Sidak's post hoc test (K), two‐tailed unpaired Student's t test (H and L). Survival curves were analyzed using a log‐rank (Mantel‐Cox) test (Q and S). Error bars, mean ± SEM. ^∗^ *p* < 0.05; ^∗∗^ *p* < 0.01; ^∗∗∗^ *p* < 0.001.

To validate this hypothesis, we first analyzed differentially expressed genes (DEGs, Table S5) between Csmd3^+^ microglia and other microglial subsets, and found that the top four upregulated genes in Csmd3^+^ microglia were major histocompatibility complex class II (MHC‐II) signature genes—*Cd74*, *H2‐Aa*, *H2‐Ab1*, and *H2‐Eb1*—highlighting their pronounced dendritic cell (DC)‐like, antitumor properties (Figure 6A). DC‐like tumor‐associated macrophages (TAMs) are enriched in type II interferon responses and T cell activation, among other antitumor processes [68], many of which were prominently represented in GO enrichment analysis of Csmd3^+^ microglia (Figure 6B and Table S5). Additionally, Csmd3^+^ microglia exhibited elevated expression of microglial homeostatic genes (*P2ry12*, *Tmem119*, *Hexb*, *Cx3cr1*) and the phagocytosis‐associated gene *Mertk* [62], further supporting their potential antitumor role (Figure 6A). Moreover, DC‐like TAMs possess both MHC class I– and class II–mediated antigen‐presenting capabilities, which enhance antitumor activity [68, 69]. Ligand–receptor analysis revealed that in 2°‐LTS (ICu) mice, Csmd3^+^ microglia exhibited enhanced MHC‐II–CD4 signaling and the emergence of MHC‐I–mediated activation of CD8^+^ T cells, potentially representing a key mechanism underlying the therapeutic efficacy observed in these mice (Figure 6C).

Moreover, the four microglial subsets identified in the TME^Med^ G422^TN^‐GBM model appeared to follow a potential differentiation trajectory, with Hist1h3c^+^ microglia—marked by high expression of proliferative genes such as Mki67 and Top2a—representing the early stage, and Csmd3^+^ microglia defining the terminal state (Figure S36A,B). Along this trajectory, the expression of MHC‐II genes, phagocytic markers, homeostatic microglial genes, and pro‐inflammatory cytokines (e.g., Il1b and Il6) progressively increases with rising *Csmd3* expression (Figure S36C). Analysis of the TCGA dataset further revealed strong positive correlations between the Csmd3^+^ microglia transcriptional signature and genes related to MHC‐I, MHC‐II, microglial homeostasis, phagocytosis, pro‐inflammatory cytokines (IL1B, TNF, IL6), and T cell markers (Figure S37). Collectively, these findings indicate that Csmd3^+^ microglia exhibit a pro‐inflammatory, antitumor phenotype.

To experimentally validate the phenotype and function of Csmd3^+^ microglia, we overexpressed *Csmd3* in BV2 microglial cells using a CRISPR/dCas9 activation system, generating MG^OE•^ *^Csmd3^* ^, BV2^ cells (Figure 6D–G and Figure S38). Among the three single‐guide RNAs (sgRNAs), sgRNA‐2# achieved the strongest activation (Figure 6E–G). RT‐qPCR analysis confirmed that *Csmd3* overexpression significantly increased pro‐inflammatory cytokine gene expression (Il1b, Tnf, Il6), supporting the pro‐inflammatory, antitumor phenotype of Csmd3^+^ microglia (Figure 6H). Further results suggest that the pro‐inflammatory phenotype induced by Csmd3 overexpression in microglia is associated with activation of the NF‐κB signaling pathway (Figure S39). We then established an in vitro co‐culture system with MG^OE•^ *^Csmd3^* ^, BV2^ cells, and GL261‐Luc cells at a 2:1 ratio, with or without lipopolysaccharide (LPS) treatment. Luciferase and CCK‐8 assays demonstrated that MG^OE•^ *^Csmd3^* ^, BV2^ cells (untreated) significantly suppressed GL261‐GBM cell proliferation, and these effects were further enhanced with LPS stimulation as compared to BV2 cells (MG^NC, BV2^) or blank control (Figure 6I–M).

Finally, we established in vivo co‐implantation systems by combining MG^OE•^ *^Csmd3^* ^, BV2^ cells with either GL261‐Luc or G422^TN^‐GBM cells (2:1 ratio; Figure 6N). MG^OE•^ *^Csmd3^* ^, BV2^ cells markedly suppressed both G422^TN^‐GBM and GL261‐GBM growth, while MG^NC, BV2^ cells showed mild inhibition only in GL261‐GBM (Figure 6O–S and Figure S40). Importantly, MG^OE•^ *^Csmd3^* ^, BV2^ cell implantation achieved LTS in 50% of GL261‐GBM‐bearing mice and in 18.2% of G422^TN^‐GBM‐bearing mice (Figure 6Q, S). Together, these results establish Csmd3^+^ microglia as potent mediators of anti‐GBM immunity in vitro and in vivo.

### 2.8 Csmd3^+^ Microglia Induce Tertiary Lymphoid Structure Formation and Trigger αPD‐1‐Mediated Survival Benefit in GBM

**FIGURE 7:**
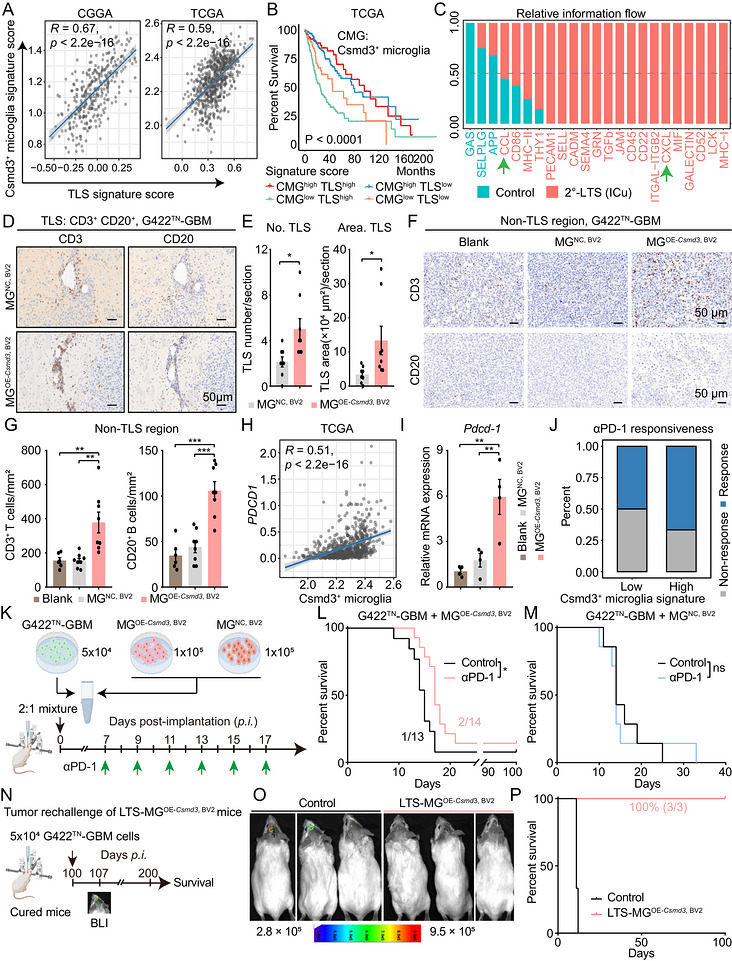
Csmd3^+^ microglia induce TME^Med^‐to‐TME^High^ subtype reprogramming and achieve immune‐cure in G422^TN^‐GBM mice. (A) Correlation plots showing a significant positive correlation between Csmd3^+^ microglia signature scores and TLS signature scores in glioma samples from the Chinese Glioma Genome Atlas (CGGA) (left panel) and The Cancer Genome Atlas (TCGA) (right panel) datasets. (B) Survival analysis of glioma patients from TCGA datasets showing a significantly longer OS in the CMG^high^TLS^high^ group than the CMG^low^TLS^low^ group (*p* < 0.001). CMG, Csmd3^+^ microglia. (C) Signaling flow analysis of cell–cell communication showing that TLS‐associated pathways (CCL and CXCL) are specifically enhanced or activated in the AGIM immune unit of ICu mice compared with control mice. (D) Representative images of TLS in the MG^NC, BV2^ group (G422^TN^‐GBM + MG^NC, BV2^ cells), and the MG^OE•^ *^Csmd3^* ^, BV2^ group (G422^TN^‐GBM + MG^OE•^ *^Csmd3^* ^, BV2^ cells) detected by sequential CD3 and CD20 staining. (E) Statistical analysis of TLS number and area in the MG^NC, BV2^ group and the MG^OE•^ *^Csmd3^* ^, BV2^ group (n = 8/group). No.TLS, TLS number; Area.TLS, TLS area. (F,G) Representative images and statistical analysis of sequential CD3 and CD20 staining in the non‐TLS region of G422^TN^‐GBM tumors from the MG^NC, BV2,^ or the MG^OE•^ *^Csmd3^* ^, BV2^ group (n = 8/group). (H) Correlation plots showing the relationship between Csmd3^+^ microglia signature scores and *Pdcd1* expression in glioma samples from The Cancer Genome Atlas (TCGA) datasets. (I) RT‐qPCR analysis showing *Pdcd1* mRNA levels in the Blank group, the MG^NC, BV2^ group, and the MG^OE‐Csmd3, BV2^ group (n = 4/group). (J) Comparison of response frequencies to αPD‐1 therapy between patients with low and high Csmd3^+^ microglia signatures, based on public bulk RNA‐seq data from the NCBI dataset PRJNA482620. Patients were stratified into high (n = 9) and low (n = 8) groups according to the median signature score. (K) Schematic illustration of the αPD‐1 treatment regimen following co‐implantation of G422^TN^‐GBM cells with either MG^OE‐Csmd3, BV2,^ or MG^NC, and BV2^ cells. αPD‐1, six doses (ip; 400 µg/mouse for the first dose, followed by 200 µg/mouse). (L) Survival curves of mice co‐implanting with G422^TN^‐GBM + MG^OE‐Csmd3, BV2^ cells with (n = 14) or without (n = 13) αPD‐1 therapy. (M) Survival curves of mice co‐implanted with G422^TN^‐GBM + MG^NC, BV2^ cells with or without αPD‐1 therapy (n = 7/group). (N) Schematic illustration of the tumor rechallenge experiment in cured mice derived from G422^TN^‐GBM + MG^OE‐Csmd3, BV2^ group in (L and Figure 6S). (O) Bioluminescence images of intracranial tumors at day 7 after tumor rechallenge in LTS mice, with the control group serving as age‐ and sex‐matched controls (n = 3/group). (P) Survival curves and LTS mice after tumor rechallenge, with the control group serving as age‐ and sex‐matched controls (n = 3/group). Survival curves were analyzed using a log‐rank (Mantel‐Cox) test (B, L and M). Statistical analysis: two‐tailed unpaired Student's t test (E), one‐way ANOVA followed by Tukey's post hoc test (G and I). Error bars, mean ± SEM. ^∗^ *p* < 0.05; ^∗∗^ *p* < 0.01; ^∗∗∗^ *p* < 0.001.

The observed association between Csmd3^+^ microglia and durable AGIM suggests their potential to reprogram the GBM TME. We therefore examined whether Csmd3^+^ microglia could drive the transition of the GBM TME from an immune‐cold to an immune‐hot phenotype. First, we assessed their correlation with TLSs, a hallmark of TME^High^ GBM and a favorable prognostic biomarker for immunotherapy across multiple cancers [70, 71], and found a strong positive association (Figure 7A). Notably, concurrent high levels of Csmd3^+^ microglia and TLS signatures were indicative of favorable survival outcomes (Figure 7B and Tables S3 and S6). Pathway analysis further revealed that TLS‐related pathways, including CCL and CXCL signaling [19, 32], were specifically upregulated or newly activated in the AGIM unit (Figure 7C), suggesting a role for Csmd3^+^ microglia in TLS induction. Consistently, GO analysis of DEGs showed that Csmd3^+^ microglia exhibit strong capacities for immune cell recruitment, T cell activation, and angiogenesis, the latter potentially corresponding to the high endothelial venule (HEV) features of TLSs (Figure 6B). To pinpoint molecular drivers, we applied single‐cell weighted gene co‐expression network analysis (scWGCNA), which identified hub genes of Csmd3^+^ microglia that enable these cells to regulate multiple signaling pathways associated with both T and B cells (Figure S41). Importantly, immunohistochemistry analysis of consecutive brain sections for CD3 and CD20 in the TME^Med^ G422^TN^‐GBM model revealed a marked increase in both the number and area of TLSs in tumors co‐injected with MG^OE•^ *^Csmd3^* ^, BV2^ cells (Figure 7D,E). Additionally, analysis of non‐TLS regions showed an increased presence of CD3^+^ T cells and CD20^+^ B cells in the same group, highlighting the broader immune‐enhancing effect of Csmd3^+^ microglia (Figure 7F,G). Together, these results demonstrate that Csmd3^+^ microglia can promote the formation of TLSs.

GO analysis of hub genes of Csmd3^+^ microglia indicated involvement in PD‐1 signaling‐related processes (Figure S41D), and Csmd3^+^ microglia showed a strong positive correlation with *PDCD1* expression in the TCGA dataset (Figure 7H). RT‐qPCR confirmed significant upregulation of *Pdcd1* in G422^TN^‐GBM/ MG^OE•^ *^Csmd3^* ^, BV2^ tissues (Figure 7I). Importantly, analysis of bulk RNA‐seq data from GBM patients regarding αPD‐1 responsiveness revealed that, although patients with low Csmd3^+^ microglia signatures may respond to αPD‐1, those with high Csmd3^+^ microglia signatures exhibited a greater responsiveness (Figure 7J). Finally, we tested whether Csmd3^+^ microglia could sensitize αPD‐1 responsiveness in the resistant TME^Med^ G422^TN^‐GBM model. Indeed, implanting MG^OE•^ *^Csmd3^* ^, BV2^ cells but not MG^NC, BV2^ cells restored G422^TN^‐GBM's responsiveness to αPD‐1 immunotherapy (Figure 7K–M). Collectively, these results demonstrate that infiltration of Csmd3^+^ microglia into the TC region reprograms the GBM TME to a TME^High^ subtype, promotes TLS formation, and enables responsiveness to αPD‐1 therapy.

### 2.9 Csmd3^+^ Microglia Exhibit Immune‐Cure Potential by Inducing 100% ICu from Cured Mice

Our scRNA‐seq data revealed that Csmd3^+^ microglia in the TME of ICu mice adopt an activated pro‐inflammatory IIM state (Figure 3G), suggesting that they may contribute to AGIM formation and exhibit immune‐cure potential. To test this hypothesis, we rechallenged cured mice derived from G422^TN^‐GBM+MG^OE•^ *^Csmd3^* ^, BV2^ co‐implantation mice (Figure 7N). Strikingly, all cured mice successfully resisted tumor rechallenge and achieved long‐term survival (2°‐LTS, i.e., ICu) (Figure 7O,P). Taken together, these findings demonstrate that Csmd3^+^ microglia possess immune‐cure potential against GBM.

## 3 Discussion

In this study, we demonstrated that the G422^TN^‐GBM preclinical model faithfully recapitulates the TME^Med^ subtype of human GBM, providing a suitable platform to investigate TME’ dynamic. Using this model, we showed that TGF‐β inhibition reprograms the GBM TME into an immune‐inflamed TME^High^ subtype, thereby restoring αPD‐1 responsiveness and enabling LTS under RT/TMZ therapy. Upon tumor rechallenge, a subset of these mice achieved 2°‐LTS with bona fide durable AGIM. scRNA‐seq revealed that the AGIM niche in 2°‐LTS mice was enriched with memory Csmd3^+^ microglia alongside T and B cells. Notably, Csmd3^+^ microglia emerged as a novel therapeutic subset with intrinsic IIM capacity, capable of restraining GBM growth, inducing a TME^High^ phenotype, and sensitizing GBM to αPD‐1 therapy.

Increasing evidence highlights the critical role of the integral TME in cancer progression and therapeutic response [29, 30, 31, 32, 33, 72], prompting the development of GBM TME subtypes as a framework for immunologically informed patient stratification [19]. However, there is currently a lack of suitable models to study the dynamic change of GBM TME subtype. Commonly used GBM models, such as GL261, are αPD‐1 responsive with LTS (Figure S1A,B and Table S4), which did not fully recapitulate the immunosuppressive mechanisms in human GBM [15], suggesting that they align more with the TME^High^ subtype. In contrast, our independently established G422^TN^‐GBM model consistently fails to respond to αPD‐1 therapy across multiple settings [11, 12], exhibiting higher malignancy than GL261 but substantially lower intratumoral T‐cell infiltration under comparable conditions (Figures S1–S3). Integrated multi‐omics analyses confirm that G422^TN^‐GBM intrinsically recapitulates the TME^Med^ subtype of human GBM (Figure 1). TGF‐β signaling critically drives tumor invasion, immune evasion, and drug resistance [42, 43], whereas its inhibition alleviates tumor‐associated immunosuppression, converting the TME from “cold” to “hot” and sensitizing tumors to ICB therapy [44]. In the TME^Med^ G422^TN^‐GBM model, TGF‐β inhibition reprogrammed the TME to a TME^High^ phenotype, restoring αPD‐1 responsiveness regardless of RT/TMZ treatment (Figure 2). In summary, the TME^Med^ G422^TN^‐GBM model provides a suitable platform for investigating the mechanisms underlying GBM subtype transitions and cold‐to‐hot tumor reprogramming.

Furthermore, TGF‐β blockade was effective only when initiated on day 3 after G422^TN^‐GBM implantation, whereas treatment beginning on days 5 or 7 failed to suppress tumor progression. These results underscore the importance of early intervention in GBM. Given the aggressive growth kinetics of both clinical GBM [6, 7] and the G422^TN^‐GBM model (Figure S2), tumors at later time points may already represent an advanced disease state in which TGF‐β inhibition alone is insufficient to achieve therapeutic control.

Durable AGIM may be key to preventing GBM recurrence, yet the underlying mechanisms driving AGIM remain unclear. To address this, we developed an innovative ICu screening paradigm based on the TME^Med^ G422^TN^‐GBM model (Figure 3C). In this paradigm, mice receiving effective treatment achieve LTS and 2°‐LTS (ICu) following tumor rechallenge, successfully establishing durable AGIM in ICu mice. scRNA‐seq analysis of the 2°‐rechallenge tumors in ICu mice reveals that the maintenance of durable AGIM may depend on the coordinated presence and interactions of memory‐like Csmd3^+^ microglia, T cells, and B cells, thereby forming the AGIM unit (Figures 3G–I and 4A–C). The concept of an AGIM unit, which encompasses the coordinated anti‐tumor activity of diverse immune cells within the TME, is increasingly recognized. Similar frameworks have been proposed, including the microglia–CD4^+^ T cell program [29], the CD4^+^–CD8^+^ T cell program [30], intratumoral immune triads involving antigen‐presenting cells, CD4^+^, and CD8^+^ T cells [31], and the tertiary lymphoid structures (TLSs) program [32, 33]. T cells play a central role in antitumor immunity, yet merely increasing the number or activity of CD8^+^ T cells is insufficient to achieve durable antitumor responses [59, 60]. We found that pathways associated with T_RM_ cell activation within the AGIM program were specifically upregulated or newly engaged. Furthermore, T_RM_ cells could be further subdivided into expanded CD4^+^ T_RM_ and CD8^+^ T_RM_ subsets, which establish crucial interactions (Figure 3L,M and Figure S24). In summary, using the ICu mouse model derived from the TME^Med^ G422^TN^‐GBM model, we identified a durable AGIM unit in an antitumor‐activated state via scRNA‐seq, which may be key to preventing GBM recurrence.

In recent years, scRNA‐seq has revealed microglial heterogeneity by defining distinct subpopulations based on gene‐specific expression [22]; however, few have been experimentally validated or functionally characterized. Here, we focused on the Csmd3^+^ microglial subset within the AGIM unit. Integrated bioinformatic and experimental analyses showed that Csmd3^+^ microglia display an anti‐tumor, pro‐inflammatory phenotype and are associated with favorable glioma prognosis. During tumor progression, they are excluded from the tumor core region, whereas effective treatment promotes their infiltration (Figures 4, 5), likely underpinning therapeutic efficacy. Using a CRISPRa system, we generated *Csmd3*‐overexpressing BV2 cells (MG^OE•^ *^Csmd3^* ^, BV2^) and found that this population suppressed GBM growth both in vitro and in vivo in GL261 and TME^Med^ G422^TN^‐GBM models (Figure 6). Moreover, MG^OE•^ *^Csmd3^* ^, BV2^ cells promoted T and B cell infiltration into the TME^Med^ G422^TN^‐GBM TME and facilitated TLS formation (Figure 7), demonstrating that they not only exert tumoricidal activity but also reprogram the TME toward a “hot” phenotype. Given that αPD‐1 therapy efficacy relies on interactions between CD8^+^ T cells and tumor‐associated macrophages (TAMs) [73], MG^OE•^ *^Csmd3^* ^, BV2^ cells may enhance this function by promoting T cell infiltration (Figure 7F,G). Notably, they also shifted the G422^TN^‐GBM TME to a TME^High^ subtype, sensitizing tumors to αPD‐1 therapy (Figure 7M). Collectively, Csmd3^+^ microglia within the AGIM program represent a therapeutic subset whose activation orchestrates GBM TME reprogramming and potentiates immunotherapy responses.

The pro‐inflammatory phenotype of Csmd3^+^ microglia may be associated with activation of the NF‐κB pathway, a canonical inflammatory signaling pathway [74]. Consistent with this notion, scRNA‐seq analysis revealed a higher NF‐κB pathway signature score in Csmd3^+^ microglia compared with Csmd3^−^ microglia, and NF‐κB signaling activation was further supported in BV2 microglia upon Csmd3 overexpression (Figure S39A–E). In addition, IRF5 and IRF8, key members of the interferon regulatory factor family involved in microglial inflammatory regulation [75, 76], showed an increasing trend in Csmd3^+^ microglia based on scRNA‐seq data. However, RT‐qPCR analysis in vitro showed no significant difference in IRF5 and IRF8 expression between MG^OE‐Csmd3, BV2^ and MG^NC, BV2^ cells in vitro (Figure S39F,G). This discrepancy may be attributed to differences in biological context, as the scRNA‐seq data were derived from the entire tumor microenvironment, where Csmd3^+^ microglia are likely influenced by additional extrinsic signals.

Despite the encouraging findings of this study, several important limitations should be acknowledged. First, although BV2 cells are widely used as a convenient and reproducible microglial model, they differ substantially from adult primary microglia in transcriptional programs, inflammatory responsiveness, metabolic state, and epigenetic landscape [77]. Therefore, the antitumor phenotype induced by CRISPRa‐mediated Csmd3 overexpression in BV2 cells (MG^OE‐Csmd3, BV2^) may not fully recapitulate the behavior of endogenous microglia in vivo. Further validation of MG^OE‐Csmd3, BV2^ roles and functions by using primary murine microglial cultures or human iPSC‐derived microglia is required to strengthen the conclusion. Due to the technique constrains of the CRISPRa system, we could not accomplish Csmd3 overexpression in primary microglia to validate our finding in MG^OE‐Csmd3, BV2^ (Figure S42), which deserves further investigation. Validation with human iPSC‐derived microglia not only strengthens our conclusion, but also paves microglia^Csmd3^ translation. Future studies may require simplified single‐vector CRISPRa systems or alternative delivery strategies, including non‐viral or transient engineering approaches. Despite these limitations, our study provides a proof‐of‐concept framework suggesting that engineering microglia toward a Csmd3‐associated state may represent a promising therapeutic strategy for GBM immunomodulation.

In summary, our findings highlight the translational value of the TME^Med^ G422^TN^‐GBM model and, through the ICu screening paradigm, position Csmd3^+^ microglia as a promising target for reprogramming the GBM TME and enhancing responsiveness to ICB.

## 4 Experimental Section

### 4.1 Human Glioma Specimens

This study was approved by the Medical Ethics Committee of The First Affiliated Hospital of Yangtze University (approval No. KY2024‐114‐02). Formalin‐fixed, paraffin‐embedded specimens from adult diffuse glioma patients with written informed consent were collected from the paraffin specimen repository of the Department of Pathology, Peking University First Hospital. Clinicopathological data are provided in Table S7. All procedures complied with the Declaration of Helsinki.

### 4.2 Animals

Male Kunming mice (20–25 g) and C57BL/6J mice were obtained from Hubei Biont Biological Technology Co., Ltd. Transgenic mouse strains included B6.129S6‐Tgfbr2^tm1Hlm/Nci^ (Tgfbr2^flox/flox^) mice (strain code: 01XN5; Frederick National Laboratory for Cancer Research, USA), kindly provided by Prof. Ge Gaoxiang (Center of Excellence in Molecular and Cellular Science, Chinese Academy of Sciences, China); and TMEM119‐CreER^T2^ transgenic mice (stock no. 012849; The Jackson Laboratory, USA), generously provided by Prof. Song Qin (Fudan University, Shanghai, China), which were crossed to generate TMEM119‐CreER^T2^;Tgfbr2 ^flox/flox^ mice. All mice were group‐housed in the Animal Core Facility of Tongji Medical College under a 12‐h light/dark cycle with ad libitum access to food and water. All procedures were approved by the Institutional Ethics Committees of Huazhong University of Science and Technology ([2023] IACUC Number: 4917) and complied with the National Institutes of Health (NIH) and the ARRIVE guidelines.

### 4.3 Cells

G422^TN^‐GBM cells, genetically corresponding to human IDH wild‐type (IDHwt) GBM, were purified from orthotopic murine G422 tumors and maintained exclusively through in vivo passaging, as they cannot be propagated in vitro [11, 12]. For experimental expansion, G422^TN^‐GBM cells were subcutaneously inoculated into Kunming mice, followed by tumor dissociation and filtration through a 75 µm mesh. To restore cell viability after digestion, G422^TN^‐GBM cells were briefly cultured in RPMI‐1640 Medium (Servicebio, G4351, Wuhan, China) supplemented with 10% fetal bovine serum (FBS; HYcezmbio, FBS500‐H, Wuhan, China) and 1% Penicillin‐Streptomycin Solution (HYcezmbio, HYG2222, Wuhan, China).

GL261 (murine GBM cell line), widely regarded as having an IDHwt GBM molecular phenotype [78, 79], was kindly provided by Prof. Wei‐Min Wang (Department of Immunology, School of Basic Medicine, Tongji Medical College, Huazhong University of Science and Technology). Luciferase‐overexpressing GL261 (GL261‐Luc) cells were kindly provided by Prof. Xiaobing Jiang (Department of Neurosurgery, Union Hospital, Huazhong University of Science and Technology, China). BV2 (murine microglial) and HMO6 (human microglial) cell lines were generously provided by Huazhong University of Science and Technology Union Hospital.

GL261, GL261‐Luc, BV2, and HMO6 cells were maintained in high‐glucose Dulbecco's modified Eagle's medium (DMEM, Servicebio, G4515, Wuhan, China) supplemented with 10% FBS and 1% Penicillin‐Streptomycin Solution, under 5% CO_2_ at 37 °C. Cells were used at passages below 50.

Primary microglia were isolated from postnatal day 1 wild‐type C57BL/6 mice. Briefly, the cerebral cortices were dissected, and the meninges were carefully removed. The tissues were gently minced and enzymatically dissociated with 0.125% trypsin for 15 min to generate a single‐cell suspension. Cells were seeded into T75 flasks and cultured in DMEM supplemented with 15% FBS and 1% Penicillin‐Streptomycin Solution. After 3 days, the medium was replaced with fresh complete DMEM containing 20 ng/mL GM‐CSF (Beyotime, P6006, China). After 7 days of culture, microglia were detached by vigorous shaking and collected from the culture supernatant. This procedure was repeated every 3 days thereafter to increase the yield of microglial cells.

### 4.4 Orthotopic GBM Mouse Models

The orthotopic G422^TN^‐GBM murine model was established as previously described [11, 12]. Briefly, Kunming mice were anesthetized via intraperitoneal injection of chloral hydrate (350 mg/kg). Freshly isolated G422^TN^‐GBM cells from subcutaneous tumors were cultured for 4 h to recover their activity, and then 5 × 10^4^ cells in 1µl PBS were stereotactically injected into the right striatum (coordinates: 0.5 mm anterior and 2.0 mm lateral from the bregma, and 3.5 mm deep) using a 10‐µL Hamilton syringe. For transgenic TMEM119‐CreER^T2^; Tgfbr2 ^flox/flox^ mice, tamoxifen (TAM, MedChemExpress, HY‐13757A, China) was administered by oral gavage at 100 mg/kg for 7 consecutive days, followed by a 7‐day withdrawal, before orthotopic injection of 5 × 10^3^ G422^TN^‐GBM cells.

For the GL261‐GBM model, male C57BL/6J mice were anesthetized and stereotactically injected with varying numbers of GL261 or GL261‐Luc cells (5 × 10^4^ cells in 2µl PBS, 1 × 10^5^ in 2µl PBS, or 2 × 10^5^ in 2µl PBS) into the right striatum as required, following the same procedure. Then, mice were regularly monitored for body weight and general condition, and no unexpected adverse events were observed.

### 4.5 RT/TMZ, TβRI‐In, αTGF‐β, and αPD‐1 Treatments

G422^TN^‐GBM‐bearing mice received therapy starting on day 3, 5, or 7 post‐implantation (*p.i*.), as previously described [11, 12]. For radiotherapy (RT), anesthetized mice were positioned prone and subjected to whole‐brain irradiation (WBI) with a single dose of 10 Gy using an X‐ray irradiator (RS2000pro‐225, Rad Source Technologies, USA) under fixed parameters (225 kV, 12 mA, 1 Gy/48.4 s). A reflector ensured uniform irradiation, while the remainder of the body was shielded with a 3‐mm lead plate.

For TMZ therapy, TMZ (M2129, AbMole, China) was dissolved in 0.5% Carboxymethylcellulose sodium (CMC‐Na, C8621, Solarbio, China) at 5 mg/mL and administered daily by oral gavage at 50 mg/kg, according to the corresponding therapeutic schedule. The treatment course consisted of 5 consecutive days of dosing, followed by a 2‐day interval, and another 5 days of dosing. On the first day of combined RT/TMZ treatment, TMZ was administered 2 h after radiation.

For TGF‐β receptor I inhibitor (TβRI‐In) therapy, galunisertib (LY2157299, S2230, Selleck, USA) was dissolved in 1.0% CMC‐Na to a final concentration of 5 mg/mL and administered by oral gavage at 50 mg/kg twice daily.

For αTGF‐β therapy, αTGF‐β (clone 1D11.16.8, BE0057, Bio X Cell, USA) was given via intraperitoneal injection at 200 µg per mouse every other day for a total of 6 doses.

For αPD‐1 therapy, αPD‐1 (clone RMP1‐14, BE0146, Bio X Cell, USA) was administered intraperitoneally, with an initial dose of 400 µg per mouse, followed by 200 µg per mouse every other day for a total of 6 doses. Control mice received isotype‐matched murine IgG according to the same schedule. When multiple drugs were administered via the same route, a 2‐h interval was required. Subsequently, mice were monitored daily and euthanized upon development of morbidity, including hunched posture, lethargy, impaired mobility, or >20% body weight loss.

### 4.6 Bioluminescence (BLI) and Whole‐Brain GFP (wbGFP) Imaging

G422^TN^‐GBM cells stably overexpressing luciferase and GFP were used for in vivo BLI and wbGFP imaging to monitor tumor volume [11, 12]. For BLI, mice bearing G422^TN^‐GBM or GL261‐Luc tumors received an intraperitoneal injection of sterile D‐luciferin potassium (15 mg/mL in PBS; Cayman Chemical Company, 14681, USA) at 10 µL/g of body weight, and intracranial tumors were imaged 10 min later using a small‐animal in vivo optical imaging system in luminescence mode (Lago X, USA). For wbGFP imaging, mice were euthanized, perfused with paraformaldehyde, and the whole brain was harvested. Intracranial G422^TN^‐GBM tumors were then imaged in fluorescence mode using the same system. Regions of interest (ROIs) were quantified using Amiview software (Spectral Instruments Imaging, USA) for statistical analysis of optical density values.

### 4.7 Tumor Rechallenge Assay

Mice bearing G422^TN^‐GBM tumors that survived for over 100 days following effective treatment, indicative of LTS or cure, were subjected to the first tumor rechallenge assay. Briefly, 5 × 10^4^ G422^TN^‐GBM cells in 2µl PBS (same as the initial implantation) were stereotactically injected into the left striatum of cured mice (coordinates: 0.5 mm anterior and 2.0 mm lateral from the bregma, and 3.5 mm deep). Age‐matched naïve male mice served as controls and received the same number of G422^TN^‐GBM cells in the left striatum. No additional treatment was administered; intracranial tumors were monitored by BLI on day 7 *p.i*., and mouse survival was recorded. Cured mice that survived more than 100 days again, indicative of 2°‐LTS (i.e., ICu), were subjected to a second tumor rechallenge (2°‐rechallenge) with 5 × 10^4^ G422^TN^‐GBM cells in the right striatum for subsequent experiments. Mice were monitored daily and euthanized upon development of morbidity, including hunched posture, lethargy, impaired mobility, or >20% body weight loss.

### 4.8 Hematoxylin & Eosin (H&E) Staining and Immunohistochemistry (IHC)

H&E staining and IHC were performed as previously reported [11, 63]. Briefly, paraffin‐embedded mouse brains were sectioned at 5 µm thickness. For H&E staining, sections were deparaffinized, rehydrated, and stained with hematoxylin for 5 min, followed by differentiation in 1% acid alcohol and counterstaining with eosin for 2 min.

For IHC, sections were deparaffinized, rehydrated, endogenous peroxidase activity was blocked, and antigens were retrieved. Sections were blocked with 5% bovine serum albumin (BSA, GC305010, Servicebio, Wuhan, China) and incubated with primary antibodies followed by corresponding HRP‐conjugated goat anti‐rabbit/mouse IgG secondary antibodies, and finally visualized using a 3,3′‐diaminobenzidine (DAB) substrate kit (G1212, Servicebio, Wuhan, China). Primary antibodies included: anti‐CD3 (1:2000, ab237721, Abcam, UK), anti‐CD4 (1:2000, ab183685, Abcam, UK), anti‐CD8 (1:2000, ab209775, Abcam, UK), anti‐CD20 (1:400, ab122222, Abcam, UK), anti‐IBA‐1 (1:500, Servicebio, GB12105, Wuhan, China), anti‐IL‐1β (1:1000, Servicebio, GB11113, Wuhan, China), anti‐Ki67 (1:200, Abcam, ab16667, UK), anti‐CD31 (1:400, Abcam, ab182981, UK). Notably, when assessing TLSs, serial sections should be used for simultaneous CD3 and CD20 staining [32, 33]. After staining, sections were dehydrated, mounted with coverslips using mounting medium, and imaged with an automated slide scanning system (SV120, Olympus, Tokyo, Japan).

### 4.9 Immunofluorescence (IF) and Tyramine Signal Amplification (TSA)

IF and TSA staining were performed as previously described [11, 63]. Briefly, paraffin‐embedded mouse brain sections were deparaffinized, rehydrated, subjected to antigen retrieval, blocked with 5% BSA, incubated with primary antibodies, and then with Alexa Fluor 488‐conjugated goat anti‐rabbit IgG (1:200, Jackson ImmunoResearch, 111‐545‐003, Pennsylvania, USA) and DyLight 594‐conjugated goat anti‐mouse IgG (1:200, Abbkine, A23410, Wuhan, China). The primary antibodies included anti‐CD31 (1:500, Abcam, ab182981, UK), anti‐IBA‐1 (1:200, GB12105, Servicebio, China), anti‐CSMD3 (1:200, orb2197, Biorbyt, UK), anti‐TMEM119 (1:400, CST, 90840T, USA).

For multiplex immunofluorescence, conventional IF was combined with the TSA method [11, 63]. Three dual‐marker pair—TMEM119/IBA‐1, CSMD3/IBA‐1, and CD3/CD20—were sequentially detected using TSA with the corresponding primary antibodies and polymer horseradish peroxidase (polyHRP)‐conjugated secondary antibodies. TSA signals were visualized with tyramide‐Cy3 and tyramide‐FITC, which are HRP‐activated and covalently deposited onto tyrosine residues of nearby proteins recognized by the primary antibodies. After TSA detection of these dual‐marker pairs, all non‐covalently bound antibodies were stripped by microwave treatment and washing. Subsequent multiplex IF staining for additional antigens was then performed using conventional IF as described above. Stained sections were imaged with an automated slide scanning system (SV120, Olympus, Japan).

### 4.10 Preparation of Single‐Cell Suspensions from Mouse GBM

2°‐LTS and age‐matched control mice were simultaneously inoculated with 5 × 10^4^ G422^TN^‐GBM cells. On day 7 *p.i*., fresh brain tissue containing tumors were harvested, ensuring comparable location and size across samples, and were enzymatically dissociated in a digestion buffer containing 1.2 mg/mL Collagenase IV (C8160, Solarbio, Wuhan, China) and 30 U/mL DNase I (D8071, Solarbio, Wuhan, China) for 20 min at 37°C, followed by filtration through a 70‐µm mesh and centrifugation at 1,200 rpm for 5 min. To remove red blood cells, the pellet was resuspended in red blood cell lysis buffer (G2015, Servicebio, Wuhan, China) and incubated for 2 min at room temperature, then diluted with two volumes of 1×PBS and centrifuged at 1200 rpm for 5 min. Immune cells were enriched by Percoll density gradient centrifugation: cell pellets were resuspended in 3 mL of 30% Percoll (17‐0891‐01, GE HealthCare, USA) prepared in RPMI‐1640 medium (G4532, Servicebio, Wuhan, China) with 10% FBS, overlaid sequentially onto 3 mL of 37% Percoll and 3 mL of 70% Percoll in a 15‐mL centrifuge tube, and centrifuged at 800 × g for 30 min at 4°C. Cells at the interphase (“white layer”) were collected, washed, and resuspended in precooled PBS containing 2% FBS, counted, and adjusted to the appropriate concentration for library construction and sequencing.

### 4.11 Single‐Cell RNA Sequencing (scRNA‐Seq)

Live cells isolated from tumor‐bearing brain tissues of G422^TN^‐GBM mice were loaded onto Chromium microfluidic chips (Single Cell 3′ Library & Gel Bead Kit v2, 10× Genomics, USA) and barcoded using a 10× Chromium Controller. Barcoded RNA was reverse‐transcribed, and sequencing libraries were generated with the Chromium Single Cell 3′ v2 reagent kit (10× Genomics) following the manufacturer's protocol. Purified libraries were then sequenced on an Illumina NovaSeq 6000 platform.

### 4.12 Processing of scRNA‐Seq Data

Raw sequencing data were processed and aligned to the mm10 mouse reference genome using CellRanger (v.7.1.0, 10x Genomics). The resulting filtered feature‐barcode matrices containing molecular counts were used as input for downstream analysis in R (v4.2.3) using the Seurat package (v.4.3.0) [80]. Prior to integration, doublet scores for each cell were predicted using scrublet (v.0.2.3) [81] in Python and DoubletFinder (v.2.0.3) [82] in R, and cells with a score > 0.15 were excluded. Seurat objects were created for each sample group, and quality control was performed to remove cells expressing < 200 or > 10 000 genes, as well as cells with > 15% mitochondrial gene expression. Highly Variable features were identified using FindVariableFeatures, followed by normalization (NormalizeData) and scaling (ScaleData). Principal component analysis (PCA) was performed on the top 2000 highly variable genes using RunPCA. To correct for batch effects across samples, the harmony algorithm (v.1.2.3) [83] was applied. Dimensional reduction was performed using UMAP or t‐SNE on the first 15 Harmony‐corrected principal components. The first 15 principal components were used to compute shared nearest neighbors (SNN) via FindNeighbors, and cell clusters were identified using FindClusters with a resolution of 0.8.

### 4.13 Cell Type Annotation and Identification of Signature Genes for Distinct Microglial Subpopulations

To identify cell types, the FindAllMarkers function (assay = “RNA”, logfc.threshold = 0.25) was applied to determine cluster‐specific differentially expressed features. Cell annotation was then performed based on these markers, with reference to annotations from the SingleR package (v.2.0.0) [84]. To distinguish G422^TN^‐GBM cells from non‐tumor cells, we applied the inferCNV algorithm (v.1.14.2) [85] to infer copy number variation (CNV) events across chromosomes. Briefly, the raw count matrix, cell annotation file, and gene/chromosome position file were prepared according to the data requirements. T cells were used as reference normal cells, and the analysis was performed with default parameters (cutoff = 0; denoise = 0.2). For microglial subclustering, FindMarkers was used to identify differentially expressed genes (DEGs) between each microglial cluster and all other microglia, and clusters were designated according to the gene with the highest pct.1/pct.2 ratio. T‐cell subclustering was performed based on conventional CD4 and CD8 marker expression. Following cell type annotation, signature genes for each cell type were again identified using FindAllMarkers. The top 100 genes from each cell type were selected to generate heatmaps using the CompleHeatmap package (v.2.14.0) [86]. Distinct features of individual microglial subpopulations, as well as total microglia, were identified by selecting genes with log2FC > 0.25 and adjusted *p* < 0.05, then ranking by descending log2FC to define the top 150 genes (Table S6).

### 4.14 Pseudotime Trajectory Analysis of Microglia and T Cells

In our scRNA‐seq dataset, microglia and T cells were further subdivided into distinct subclusters based on transcriptional profiles and biological relevance. Prior to trajectory reconstruction, stringent quality control was applied to exclude cells with low transcript counts, high mitochondrial gene content, or aberrant gene complexity. Monocle2 (v.2.24.1) [87] was then employed to investigate pseudotemporal gene expression dynamics and phenotypic transitions across microglial and T‐cell subpopulations. Guided by biological prior knowledge, subclusters with high expression of proliferative markers such as *Ki67* and *Top2a* were designated as the root states for trajectory ordering. Approximately 2000 pseudotime‐associated genes were identified using the *differentialGeneTest* function and subsequently utilized for dimensionality reduction and trajectory construction. Finally, the biological characteristics of each subcluster were inferred by integrating pseudotime‐dependent expression dynamics with established functional annotations, thereby providing insights into lineage progression and functional heterogeneity within the tumor microenvironment.

### 4.15 Co‐Expression Network Analysis in Microglia

Co‐expressed gene modules in microglia were identified using the hdWGCNA package (v.0.4.0) [88]. Genes expressed in at least 5% of all cells were retained to construct expression matrices. Meta‐cells were generated by aggregating 25 neighboring cells (k = 25) with a maximum of 10 shared cells (max_shared = 10) using the MetacellsByGroups function. Network construction and module identification were performed with ConstructNetwork, applying a soft‐thresholding power of 6. Module eigengene (ME) scores were calculated using AddModuleScore from Seurat, and visualized using ModuleFeaturePlot and ModuleRadarPlot from hdWGCNA, as well as DotPlot from Seurat. Finally, Gene Ontology (GO) enrichment analysis for genes in the turquoise module was performed using enrichGO from the clusterProfiler package (v.4.6.2) [89].

### 4.16 Integrated Analysis of scRNA‐Seq and snRNA‐Seq Data in the G422^TN^‐GBM Model

ScRNA‐seq and snRNA‐seq each offer distinct advantages for resolving diverse cell types [90, 91]. In our previous work, snRNA‐seq of the G422^TN^‐GBM model at day 7 *p.i*. was performed and deposited in the GEO database (BioProject ID PRJNA1043813). To obtain a more comprehensive characterization of the G422^TN^‐GBM model, we integrated scRNA‐seq and snRNA‐seq datasets from day 7 tumors using the harmony algorithm (v.1.2.3) [83]. Quality control and preprocessing followed the same pipeline as described for scRNA‐seq. Unlike the scRNA‐seq analysis, however, clustering of the integrated dataset was performed using the FindClusters function with a resolution of 0.5. Cell type annotation was carried out based on the scRNA‐seq–derived markers, and the annotated integrated dataset was subsequently combined with spatial transcriptomic data from day 7 G422^TN^‐GBM tumors for downstream analysis.

### 4.17 Non‐Negative Matrix Factorization (NMF) Analysis of G422^TN^‐GBM Cells

To systematically decompose the transcriptional landscape of G422^TN^‐GBM cells and uncover latent gene expression programs underlying tumor heterogeneity, we applied non‐negative matrix factorization (NMF) using the NMF R package (v.0.23.0) [92, 93]. The input matrix consisted of log‐normalized expression values from G422^TN^‐GBM cells in the integrated single‐cell transcriptomic dataset, with lowly expressed genes filtered to minimize background noise. NMF was performed over a range of factorization ranks (k = 3–15) using the “brunet” algorithm with 50 iterations per rank and a fixed random seed to ensure reproducibility. The optimal rank (k = 4) was determined based on a comprehensive evaluation of the cophenetic correlation coefficient, residual sum of squares, and silhouette width. Robust NMF programs were subsequently clustered according to Jaccard similarity using a customized iterative algorithm, which merged highly overlapping programs into clusters. Each resulting metaprogram was defined by the top 50 most recurrent genes within its cluster, yielding a total of four distinct metaprograms. This NMF‐based framework enabled a modular dissection of gene expression patterns, thereby revealing diverse cellular states and functional heterogeneity within the G422^TN^‐GBM compartment.

### 4.18 Cell–Cell Interaction Analysis

To explore cell–cell communication within the integrated single‐cell transcriptomic dataset from G422^TN^‐GBM‐bearing mice and the scRNA‐seq dataset from ICu mice, we applied CellChat (v.1.6.1) [94, 95] using default parameters, leveraging its curated ligand–receptor database to infer putative intercellular signaling interactions. Beyond mapping the global communication landscape, we specifically focused on interactions among endothelial cells, neurons, and G422^TN^‐GBM cells in the integrated single‐cell dataset from the G422^TN^‐GBM model, and on interactions among microglia, T cells, B cells, and G422^TN^‐GBM cells in the ICu mouse model dataset. Network graphs, heatmaps, and hierarchical pathway clustering were generated using CellChat's built‐in functions to visualize intercellular signaling architecture comprehensively. This approach provides a systematic framework for dissecting intercellular dynamics and offers mechanistic insights into the coordinated interplay between immune/non‐immune and tumor cell populations within the glioblastoma microenvironment.

### 4.19 Visium Spatial Transcriptomics

Fresh brain tissue was dissected on dry ice following a 4‐min perfusion with pre‐cooled saline and immediately embedded in optimal cutting temperature (OCT) compound on day 7 *p.i*. of 5×10^4^ G422^TN^‐GBM cells. RNA quality of the OCT‐embedded block was assessed using an Agilent 2100 system, and tissues with an RNA integrity number (RIN) greater than 4 were selected for Visium spatial gene expression experiments. The OCT blocks were then sectioned into 10 µm slices using a Leica CM3050s cryostat. Sections were mounted on the Sigma–Aldrich Poly Prep Slide following the Visium CytAssist Spatial Gene Expression for Fresh Frozen Tissue Preparation Guide (10x Genomics, CG000636). To identify the optimal tumor section, methanol fixation and H&E staining were performed according to the Demonstrated Protocol VisiumCytAssist_FreshFrozen_H&E_RevA (10x Genomics, CG000614), and the stained sections were imaged at 20x magnification using the brightfield setting on a Leica Aperio Versa8 whole‐slide scanner. Adjacent sections were then applied to mouse whole‐transcriptome probe panels (6.5mm × 6.5mm) as described in the Visium CytAssist Spatial Gene Expression User Guide (10x Genomics, CG000495). Following hybridization of probe pairs to their target genes and ligation, slides were processed on the Visium CytAssist instrument for RNase treatment and permeabilization. The ligated probes were subsequently hybridized to spatially barcoded oligonucleotides on the Capture Area. Libraries for spatial transcriptomics were generated from these probes and sequenced on an Illumina NovaSeq 6000 system (Beijing Novogene Technology Co., Ltd.).

### 4.20 Preprocessing of Spatial Transcriptomic Data

Raw FASTQ files and histology images were processed using the short‐read probe alignment algorithm for FFPE ‘count’ method in Space Ranger (v.1.3.0, 10x Genomics) to align probe reads to the mm10 mouse reference genome. The resulting filtered gene‐spot matrices and fiducial‐aligned low‐resolution histology images were subsequently imported into the Seurat package (v.4.3.0) in RStudio for downstream analysis. Visium spots with fewer than 1000 unique molecular identifiers (UMIs), fewer than 500 detected genes, or with a mitochondrion gene fraction exceeding 15% were removed from further analysis. Data normalization, scaling, and regression were performed using the SCTransform method with vars.to.regress = “percent.mt”. After quality control and filtering, the spatial transcriptomic dataset from the G422^TN^‐GBM model was integrated with matched scRNA‐seq and snRNA‐seq datasets collected at day 7 *p.i*., and spatial gene expression patterns were visualized using the SpatialFeaturePlot function.

### 4.21 Applying Conditional Autoregressive‐Based Deconvolution (CARD) for Spatial Deconvolution of the G422^TN^‐GBM Model

To dissect the spatial cellular architecture of the G422^TN^‐GBM TME, we employed CARD (v.1.1) [96] to integrate spatial transcriptomics data with cell type–specific reference matrices derived from single‐cell transcriptomic datasets (scRNA‐seq and snRNA‐seq). CARD builds upon an NMF framework by incorporating a cellular spatial autoregression assumption, which accounts for spatial correlations among neighboring spots and thereby improves deconvolution accuracy compared with standard NMF‐based approaches. In this study, spatial transcriptomic data from the G422^TN^‐GBM model were first preprocessed to normalize gene expression and exclude low‐quality spots. Reference matrices were then constructed from annotated single‐cell transcriptomic clusters representing the major cell types in the TME. These inputs were integrated to estimate cell type proportions across spatial spots, ultimately generating high‐resolution spatial maps of cellular composition within tumor tissue.

### 4.22 Public scRNA‐Seq and snRNA‐Seq Data Processing and Analysis

Publicly available scRNA‐seq datasets from glioma patients were obtained from the GEO database (GSE182109). This dataset includes 44 samples from 2 patients with low‐grade glioma (LGG), 11 patients with newly diagnosed glioblastoma (ndGBM), and 5 patients with recurrent glioblastoma (rGBM) [61]. The scRNA‐seq dataset of the SB28‐GBM model was retrieved from the GEO database (GSE226292), in which targeting TREM2 has been shown to effectively treat GBM [64]. In addition, publicly available scRNA‐seq datasets of a microglial aging model were obtained from GEO (GSE207948, GSE226286), where three cycles of CSF1R‐targeted microglial depletion followed by repopulation were used to induce an aging‐associated microglial phenotype [62]. Our previously generated snRNA‐seq dataset, which demonstrated that CD73 inhibitor AB680 effectively suppressed G422^TN^‐GBM progression [63], has been deposited in the SRA database (PRJNA1251355) and was also used in the present study. Quality control and downstream analyses were carried out according to the same standards applied in our sc/snRNA‐seq analyses as well as those specified in the original publications. Then, sample data from different studies were integrated using the harmony algorithm (v.1.2.3) [83]. Finally, microglia were identified in these datasets using established marker genes (human: *GPR34*, *AIF1*; mouse: *Gpr34*, *Aif1*). Cells were further stratified into Csmd3^+^ and Csmd3^−^ microglial subpopulations based on *Csmd3* expression levels.

### 4.23 Public Bulk RNA‐Seq Data Processing and Analysis

Bulk RNA‐seq datasets and corresponding clinicopathological information of glioma patients were obtained from The Cancer Genome Atlas (TCGA) via the UCSC Xena browser (https://xenabrowser.net/) and from the Chinese Glioma Genome Atlas (CGGA) database (http://www.cgga.org.cn/) [97]. Additional bulk RNA‐seq datasets, including GSE16011 [98] as well as our G422^TN^‐GBM tumors (PRJNA1042906, PRJNA1298905), were retrieved from NCBI GEO and SRA. Gene expression levels were quantified as FPKM.

For functional and cell type‐specific analyses, single‐sample gene set enrichment analysis (ssGSEA) was performed using the GSVA package (v.1.46.0) [99]_,_ based on curated marker genes for specific cell subpopulations and TGF‐β signature gene sets obtained from MSigDB (http://software.broadinstitute.org/gsea/msigdb/) [100]. Patients were stratified into high‐ or low‐signature groups according to the median ssGSEA scores. Kaplan‐Meier survival analyses were conducted to evaluate the prognostic significance of gene signatures, and statistical significance assessed using the log‐rank test. Hazard ratios and 95% confidence intervals were calculated using the survfit and survdiff functions from the R survival package (v.3.5.8), providing an integrated framework for correlating transcriptional programs with clinical outcomes in glioma.

### 4.24 Generation of Csmd3‐Overexpressing Microglia Using the CRISPRa System

Due to the large genomic size and complex exon–intron structure of *Csmd3*, full‐length cDNA overexpression was not feasible. We therefore employed a CRISPR activation (CRISPRa) approach using the dCas9‐SAM system to transcriptionally upregulate the endogenous *Csmd3* locus [101]. Specifically, a three‐component CRISPRa system was implemented using the following VSVG‐LENTAI constructs: (1) VSVG‐LENTAI‐EF1a.core‐dCas9‐VP64‐2A‐BSD‐WPRE‐pA (L7130), expressing a nuclear‐localized dCas9‐VP64 activator with blasticidin resistance for stable selection; (2) VSVG‐LENTAI‐hEF1a‐MPH(MS2(N55K)‐p65‐HSF1)‐HygroR‐WPRE‐pA (L7129), expressing the MS2‐p65‐HSF1 (MPH/SAM) co‐activator under hEF1a with hygromycin resistance; (3) VSVG‐LENTAI‐hU6‐sgRNA.sp(w/MS2)‐esEF1a‐MataGFP‐IRES‐PuroR‐WPRE‐pA, encoding either a non‐targeting control sgRNA (NC, L7138) or *Csmd3*‐targeting sgRNAs (Csmd3 #1–#3, three distinct guides, WL2696–WL2698) containing MS2 hairpins for MPH recruitment, together with EF1a‐driven MataGFP and puromycin resistance for tracking and selection. Lentiviral vectors were constructed by Shanghai Taitool Bioscience Company, China. Prior to formal experiments, the optimal multiplicity of infection (MOI) for BV2 microglial cells was determined by consulting previous literature [102, 103] and testing comparable viral constructs. Minimum concentrations of blasticidin, hygromycin, and puromycin required to completely eliminate BV2 cells were also established.

BV2 cells were sequentially infected as follows: first with L7130 and selected with blasticidin for 4 days; then with L7129 and selected with hygromycin B for 7 days; and with L7138 (non‐targeting control) or WL2696–WL2698 (Csmd3‐targeting sgRNAs #1–#3) and selected with puromycin for 7 days. During each infection, 5 µg/mL polybrene was added to enhance transduction efficiency, and culture medium was replaced 12 h post‐infection. The resulting *Csmd3*‐overexpressing BV2 microglial (MG^OE•^ *^Csmd3^* ^, BV2^) cells were validated for Csmd3 upregulation by Western blotting and RT‐qPCR. BV2 microglia infected with L7138 served as the non‐targeting control (MG^NC, BV2^). For primary microglia, the three viruses were co‐transduced at an MOI ratio of 1:1:1 (total MOI = 30).

### 4.25 Western Blotting (WB) Analysis

Western blotting was performed as previously described [11, 63]. Briefly, total protein was extracted from BV2, HMO6, MG^OE•^ *^Csmd3^* ^, BV2^, and BV2 cells stimulated with 10 µM TβRI‐In for 24 h. Cells were lysed in radioimmunoprecipitation assay (RIPA) buffer containing phenylmethanesulfonyl fluoride (PMSF), and equal amounts of protein were separated by sodium dodecyl sulfate–polyacrylamide gel electrophoresis (SDS–PAGE) and transferred onto 0.45 µm nitrocellulose (NC) membranes (Merck Millipore, Ireland). Membranes were incubated with the primary antibodies, including anti‐CSMD3 (1:1000, orb2197, Biorbyt, UK), anti‐p‐NF‐κB‐P65 (1:5000, AP1294, ABclonal, China), anti‐ NF‐κB‐P65 (1:10000, 80979, Proteintech, China), and anti‐β‐actin (1:1000, GB11001, Servicebio, Wuhan, China) as the loading control. Horseradish peroxidase–conjugated secondary antibody was applied, and protein bands were subsequently detected and quantified using an enhanced chemiluminescence system (Bio‐Rad, California, USA).

### 4.26 RNA Extraction and Quantitative Reverse Transcription PCR (RT‒qPCR)

RT–qPCR was performed as previously described [11, 63]. In brief, total RNA was extracted from orthotopic tumor tissues and cultured microglia using TRIzol reagent (Invitrogen, 15596‐026, Massachusetts, USA) following the manufacturer's protocol. Subsequently, 0.1 µg of RNA was reverse‐transcribed into cDNA using the HiScript III RT SuperMix for qPCR with gDNA wiper (Vazyme, R323‐01, Nanjing, China). RT–qPCR assays were constructed with ChamQ Blue Universal SYBR qPCR Master Mix (Vazyme, Q312, Nanjing, China) on a Quantagene q225 real‐time PCR system to assess the expression levels of *Il1b, Ctla4, Ccl5, Il23a, P2ry12, Cd274, Il2, Il12a, Il12b, Tnf, Il6, Csmd3, Pdcd1, Rela, Irf5, Irf8* and *Actb*. Primer sequences were listed in Table S8. Relative expression levels were calculated using the 2^−ΔΔCT^ method, with β‐actin as the internal reference.

### 4.27 In Vitro Co‐Culture Assays for GBM Cell Proliferation and Viability

Direct and indirect co‐culture systems were established to evaluate the effects of MG^OE•^ *^Csmd3^* ^, BV2^ cells on the proliferation and viability GL261‐Luc cells. For the direct co‐culture, 1 × 10^5^ MG^OE•^ *^Csmd3^* ^, BV2^ cells were mixed with 5 × 10^4^ GL261‐Luc cells (2:1 ratio) in 96‐well plates and cultured for 24 h with or without 0.2 µg/mL lipopolysaccharide (LPS, GC205009, Servicebio, Wuhan, China). At 0 h and 24 h, D‐Luciferin potassium (0.15 mg/mL, Cayman Chemical, USA) was added and incubated for 3 min at 37°C, followed by luminescence imaging using an in vivo optical imaging system (Lago X, USA). Control wells included GL261‐Luc cells alone (blank control) and MG^NC, BV2^ cells (non‐targeting control). The regions of interest (ROI) were quantified using Amiview software (Spectral Instruments Imaging Company, USA) for statistical analysis of luminescence density.

For the indirect co‐culture, 1 × 10^5^ MG^OE•^ *^Csmd3^* ^, BV2^ cells were seeded in the upper chamber of a 0.4 µm pore Transwell insert, while 5 × 10^4^ GL261‐Luc cells were seeded in the lower chamber. Identical control groups were included. After 24 h, GL261‐Luc cells were harvested from the lower chamber by digestion with 2.5% trypsin, transferred to 96‐well plates, and incubated with CCK‐8 reagent (G4103, Servicebio, China). Absorbance at 450 nm was measured after 1–2 h of incubation at 37°C, following the manufacturer's instructions.

### 4.28 In Vivo Co‐Implantation of MG^OE‐Csmd3, BV2^ and GBM Cells with αPD‐1 Blockade

In vivo co‐implantation models were established using GL261‐Luc and G422^TN^‐GBM cells, as previously described [64]. Briefly, 1 × 10^5^ MG^OE•^ *^Csmd3^* ^, BV2^ cells were mixed directly with 5 × 10^4^ GL261‐Luc or G422^TN^‐GBM cells (2:1 ratio) and orthotopically injected into the right striatum according to the standard coordinates and procedures of the orthotopic GBM model. Control groups included GL261‐Luc cells or G422^TN^‐GBM alone (blank control) and MG^NC, BV2^ cells (non‐targeting control). For αPD‐1 blockade studies, we employed the αPD‐1–nonresponsive G422^TN^‐GBM model to test whether co‐implantation with MG^OE•^ *^Csmd3^* ^, BV2^ cells could confer sensitivity to αPD‐1 therapy. Specifically, αPD‐1 was administered intraperitoneally on a six‐dose schedule: with an initial 400 µg per mouse followed by 200 µg per mouse every other day. Control mice received equivalent doses of isotype‐matched murine IgG on the same schedule.

### 4.29 Statistical Analysis

Statistical details of the experiments, including sample sizes and statistical tests, are provided in the figure legends. Mean values among multiple groups were compared using one‐way ANOVA followed by Tukey's post hoc test (for all group comparisons). Mean values between two groups across time or tumor regions were compared using two‐way ANOVA followed by Tukey's post hoc test. All post hoc analyses were adjusted for multiple comparisons. Mean values between two independent groups were assessed with two‐tailed unpaired Student's *t*‐tests. Differences between two independent groups were assessed using a two‐sided unpaired Wilcoxon test (non‐parametric). Animal survival was evaluated using the Kaplan‒Meier method and compared using the log‐rank (Mantel–Cox) test. Data were presented as means ± SEM, and *P* < 0.05 was considered statistically significant. Survival curves were generated using the gsurvplot function in the R package survminer (v.0.4.2) with the Log–rank test for comparison. Patients with glioma from the TCGA, CGGA, and GSE16011 cohorts were stratified into high‐ and low‐expression groups using the surv_cutpoint function in the R package survminer. All statistical analyses were performed in R (v.4.2.3) and GraphPad Prism (GraphPad Software, USA).

## Author Contributions

H.F.J. and X.Q.C. conceived the research concept, designed the experiments, and drafted the manuscript. H.F.J., P.P.G., F.L., and Y.W.D. performed the majority of experimental work and data analysis. L.Q.W., E.Z.Y., Q.A., Z.H.D., J.W.S and Y.S. assisted with experimental execution and data interpretation. R.Q.C., F.L., M.L., and X.Q.C. contributed to manuscript writing, review, and revision. R.Q.C. and M.L. performed bioinformatics analysis. R.Q.C. and X.Q.C. provided intellectual support for the study. F.L., M.L., and X.Q.C. secured funding for the project. All authors have read and approved the final version of the manuscript.

## Funding

The National Natural Science Foundation of China (Grant No. 82173197 to XQC); the Hubei Provincial Natural Science Foundation of China (Grant No. 2024AFB978, 2026AFC0551 to FL); the Jingzhou Joint Scientific Research Fund (Grant No. 2024LHY14 to FL); the National Natural Science Foundation of China (Grant No. 32270715, 32470712, 32421003 to M.L).

## Conflicts of Interest

The authors declare no conflicts of interest.

## Supporting information




**Supporting File 1**: advs76690‐sup‐0001‐SuppMat1.docx

**Supporting File 2**: advs76690‐sup‐0002‐Table.zip.

**Supporting File 3**: advs76690‐sup‐0003‐Raw_Western_blot_images‐260606.docx.

## Data Availability

The data that support the findings of this study are openly available in GEO at https://www.ncbi.nlm.nih.gov/geo/, reference number GSE166525 and GSE253428.
