# Supplementary material for: Identifying a Csmd3+ Microglial Subpopulation that Drives Cold‐to‐Hot Transition and Immune‐Cure in Glioblastoma
Source: Adv Sci (Weinh). 2026 Jul 20:e76690. Online ahead of print. doi: 10.1002/advs.76690 (PMC13383698; doi:10.1002/advs.76690)
Supplement: Supplementary file 1 — Supporting File 1: advs76690‐sup‐0001‐SuppMat1.docx [file ADVS-9999-e76690-s002.docx]

**Identifying a Csmd3⁺ microglial subpopulation that drives cold-to-hot transition and** **immune-cure in glioblastoma**

*Hai-Feng Jiang, Pan-Pan Gao, Yu-Wen Du, Li-Qin Wu, En-Zhi Yin, Qi An, Ze-Hua Ding, Jin-Wen Shi, Ya Shu,* *Ruoqiao Chen *, Feng Liu *, Mingfeng Li *, Xiao Qian Chen **

**This PDF file includes:**

Figs. S1 to S42

Legends for tables S1 to S8

**Other Supplementary Materials for this manuscript include the following:**

Tables S1 to S8

Raw Western blot images


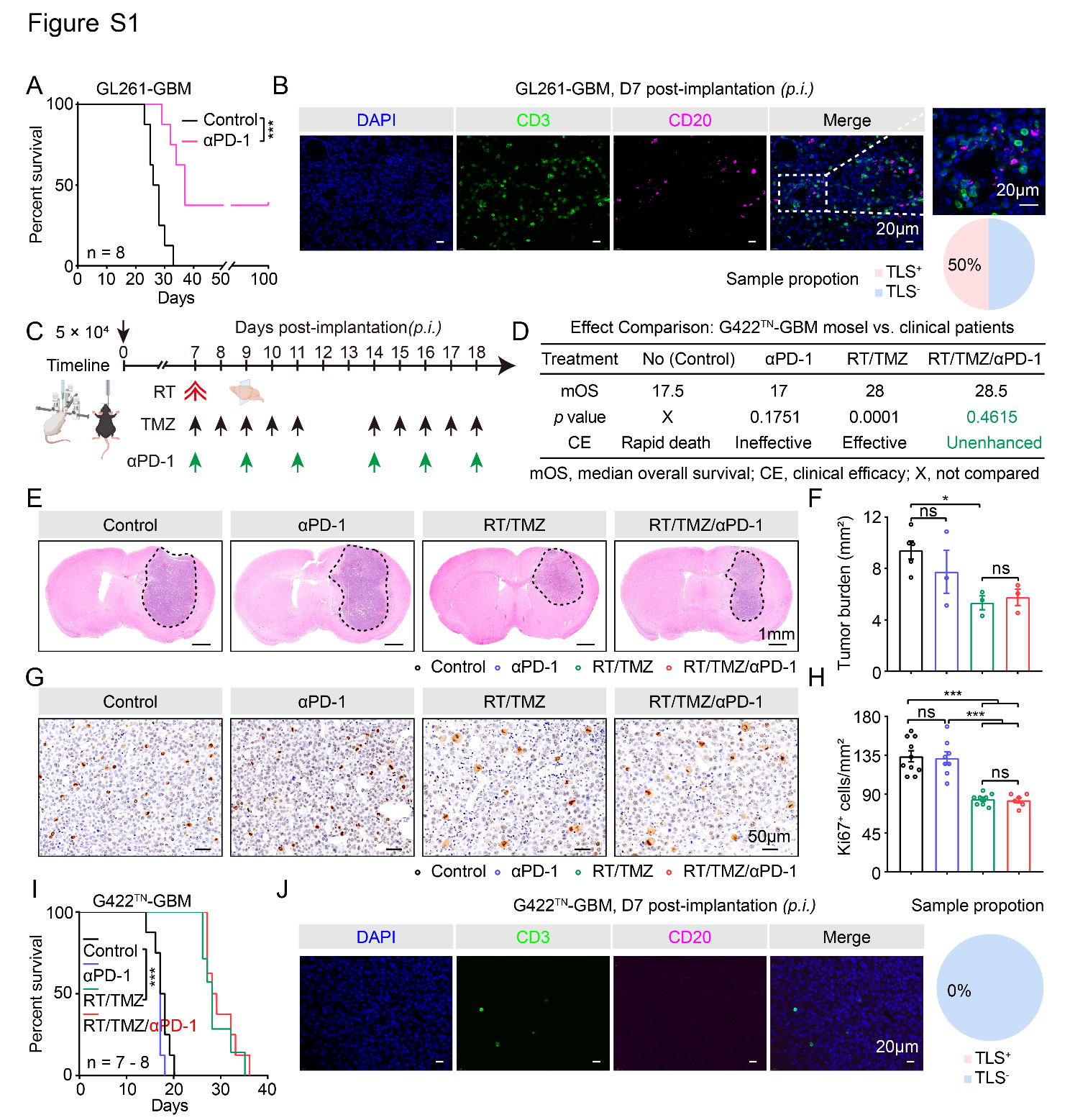


**Figure S1.** **Comparison of therapeutic responses and tertiary lymphoid structure (TLS) assessment between the G422^TN^-GBM and GL261-GBM models, related to Figure 1.**

(A) Survival curve of the GL261-GBM mice with αPD-1 treatment and control group (n = 8/group).

(B) CD3/CD20 dual immunofluorescence (IF) staining for TLS in GL261-GBM tumors and the percentage of TLS-positive (TLS^+^) samples (n = 4).

(C) Schematic illustration depicting the treatment regimens with αPD-1, RT/TMZ, and their combination initiated on day 7 *p.i.*. RT, radiotherapy, a single dose of 10 Gy whole-brain irradiation (WBI); TMZ, temozolomide, 10 doses of temozolomide (50 mg/kg, ig); αPD-1, anti-PD-1, six doses (ip; 400 μg/mouse for the first dose, followed by 200 μg/mouse). *p.i.*, post-implantation.

(D) Three-line table illustrating that the therapeutic responsiveness of the G422^TN^-GBM model to αPD-1, RT/TMZ, and their combination mirrors that observed in clinical patients. mOS, median overall survival; CE, clinical efficacy; X, not compared. Green text indicates that αPD-1 fails to further extend the therapeutic benefit of RT/TMZ.

(E-F) Representative H&E images (C) and quantitative analysis (D) of G422^TN^-GBM tumor burden in the control group (n = 5), as well as in the αPD-1, RT/TMZ, and RT/TMZ/αPD-1 treatment groups (n = 3/group).

(G-H) Representative IHC images and quantitative analysis of Ki67 staining in the control group (n = 10), as well as in the αPD-1 (n = 8), RT/TMZ (n = 8), and RT/TMZ/αPD-1 (n = 6) treatment groups.

(I) Survival curve of the G422^TN^-GBM mice with αPD-1, RT/TMZ, RT/TMZ/αPD-1 treatment and control group (n = 7-8/group).

(J) CD3/CD20 dual IF staining for TLS in G422^TN^-GBM tumors and the percentage of TLS^+^ samples (n = 4).

Survival curves were analyzed using a log-rank (Mantel-Cox) test (A and I). Statistical analysis, one-way ANOVA followed by Tukey’s post hoc test (F and G), Error bars, mean ± SEM. ^*^*p* < 0.05; ^***^*p* < 0.001; ns, not significant.


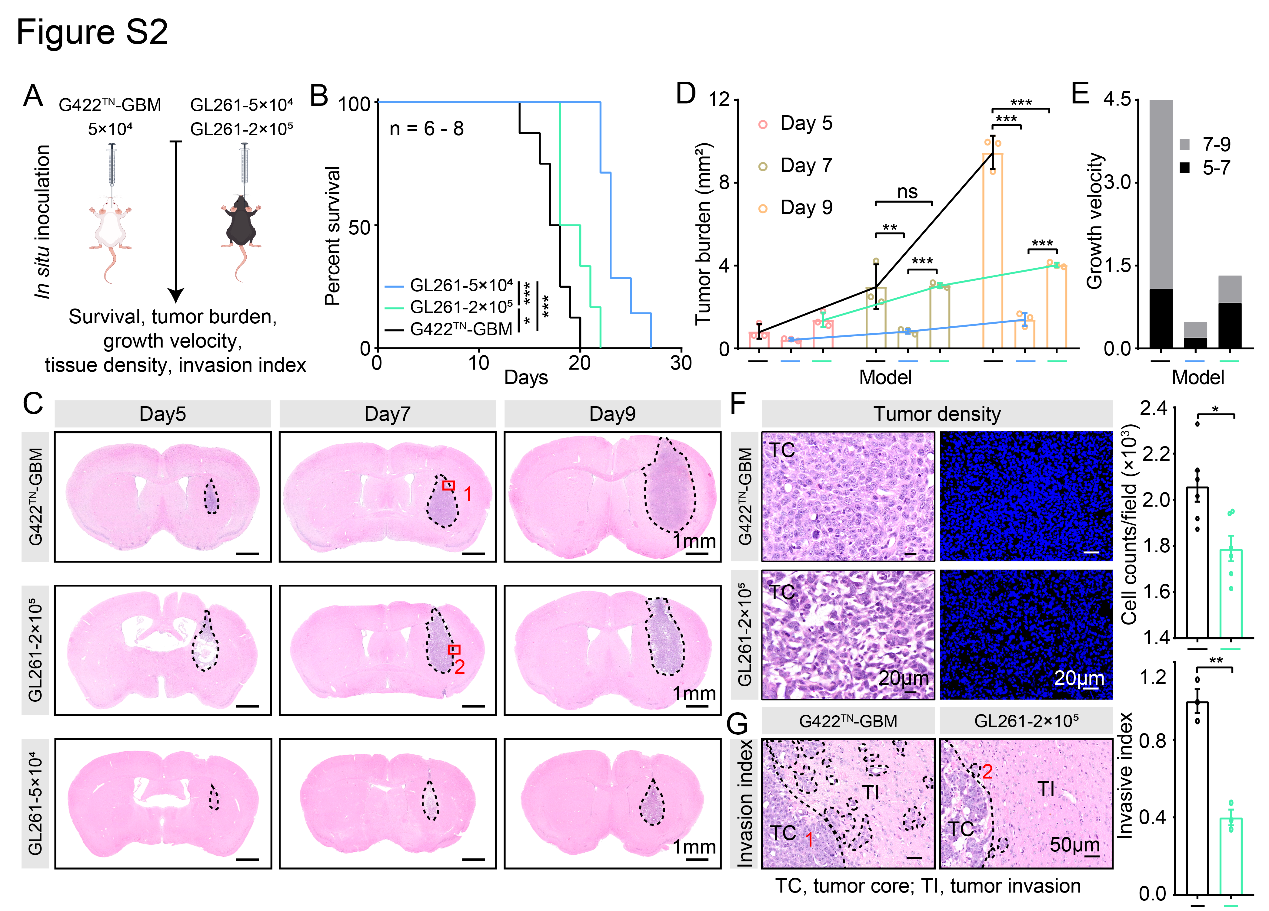


**Figure S2. Comparison of malignancy between the G422^TN^-GBM model and the GL261-GBM model, related to Figure 1.**

(A) Schematic illustration comparing the G422^TN^-GBM and GL261-GBM models (by Figdraw).

(B) Survival curves of the G422^TN^-GBM model with an initial inoculation of 5×10⁴ cells and the GL261-GBM model with initial inoculations of 5×10⁴ and 2×10⁵ cells (n = 6-8).

(C-E) Representative H&E images (C) and quantitative analyses of tumor burden (D) and growth velocity (E) in the G422^TN^-GBM model (initial inoculation of 5×10⁴ cells) and the GL261-GBM model (initial inoculations of 5×10⁴, and 2×10⁵ cells) on days 5, 7, and 9 *p.i.*.

(F) Representative images (left panel) and quantitative analyses of tumor density (right panel) in the G422^TN^-GBM model (initial inoculation of 5×10⁴ cells) and the GL261-GBM model (initial inoculations of 2×10⁵ cells) on day 7 *p.i.*, showing comparable tumor burden (n = 6/group). TC, tumor core.

(G) Representative H&E images (left panel) and quantitative analyses of invasion index (right panel) in the G422^TN^-GBM model (initial inoculation of 5×10⁴ cells) and the GL261-GBM model (initial inoculations of 2×10⁵ cells) on day 7 *p.i.*, showing comparable tumor burden (n = 3/group). TI, tumor invasion.

Survival curves were analyzed using a log-rank (Mantel-Cox) test (B). Statistical analysis, two-way ANOVA followed by Sidak’s post hoc test (D), two-tailed unpaired Student’s t test (F and G, right panels). Error bars, mean ± SEM. ^*^*p* < 0.05; ^**^*p* < 0.01; ^***^*p* < 0.001; ns, not significant.


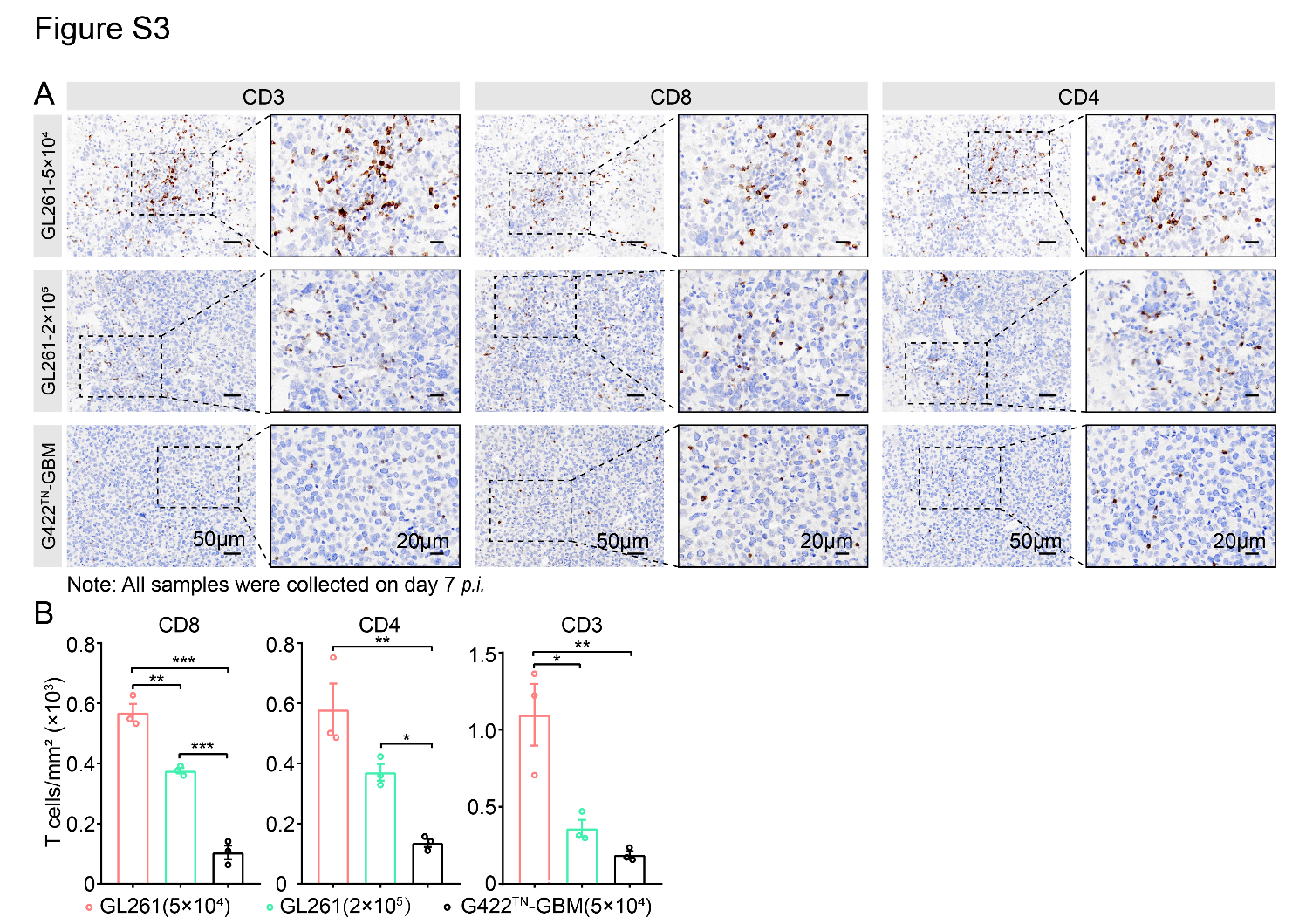


**Figure S3. Comparison of intratumor T infiltration between the G422^TN^-GBM model and the GL261-GBM model, related to Figure 1.**

(A) Representative IHC images of CD3, CD8 and CD4 staining in the G422^TN^-GBM model (initial inoculation of 5×10⁴ cells) and the GL261-GBM model (initial inoculations of 5×10⁴ and 2×10⁵ cells) on day 7 *p.i.*.

(B) Statistical analysis of CD3⁺, CD4⁺, and CD8⁺ T-cell infiltration in tumors on day 7 post-implantation (p.i.) in GL261-GBM and G422^TN^-GBM mice (n = 3/group).

Statistical analysis, one-way ANOVA followed by Tukey’s post hoc test (B). Error bars, mean ± SEM. ^*^*p* < 0.05; ^**^*p* < 0.01; ^***^*p* < 0.001.


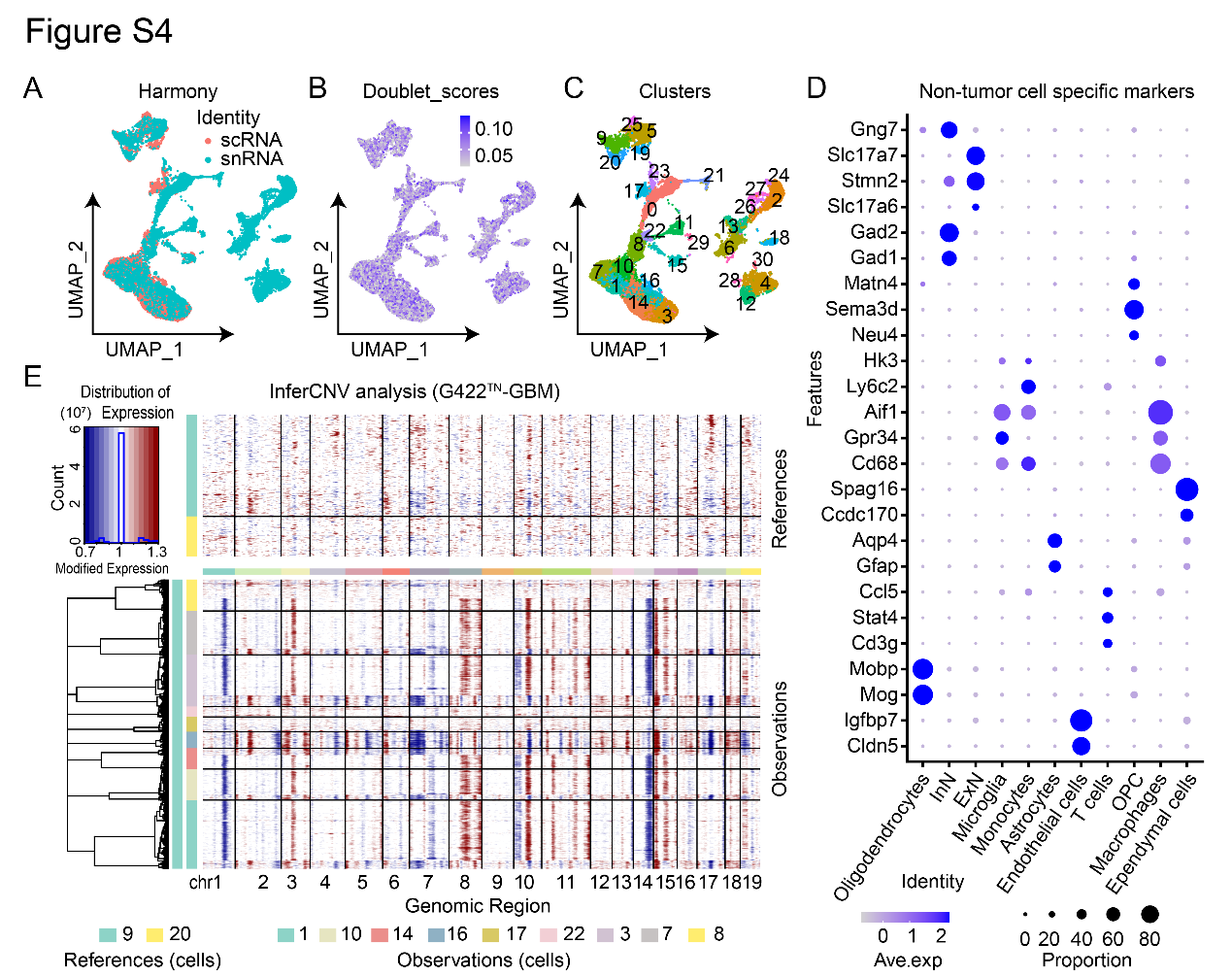


**Figure S4. Integrated analysis of scRNA-seq and snRNA-seq data from the G422^TN^-GBM model, related to Figure 1.**

(A) UMAP plot depicting the integration of scRNA-seq and snRNA-seq data using the Harmony algorithm in the G422^TN^-GBM model.

(B) UMAP plot showing doublet scores of the integrated scRNA-seq and snRNA-seq data in the G422^TN^-GBM model, calculated using Scrublet (Python package).

(C) UMAP plot showing all cell clusters in the integrated scRNA-seq and snRNA-seq data of the G422^TN^-GBM model.

(D) Dot plots showing the expression of specific marker genes across all non-tumor cell types in the integrated scRNA-seq and snRNA-seq data of the G422^TN^-GBM model. Data are plotted as log-normalized counts, and the dot size represents the proportion of cells with the given gene detected.  OPC, oligodendrocyte progenitor cells; InN, inhibitory neurons; ExN, excitatory neurons. Avg.exp, average expression.

(E) Hierarchical heatmap showing large-scale CNV profile of each G422^TN^-GBM cell cluster (1, 3, 7, 8, 10, 14, 16, 17 and 22). Red and blue colors represent high and low CNV level, respectively. T cells (cluster 9) and monocytes (cluster 20) are defined as reference cells.


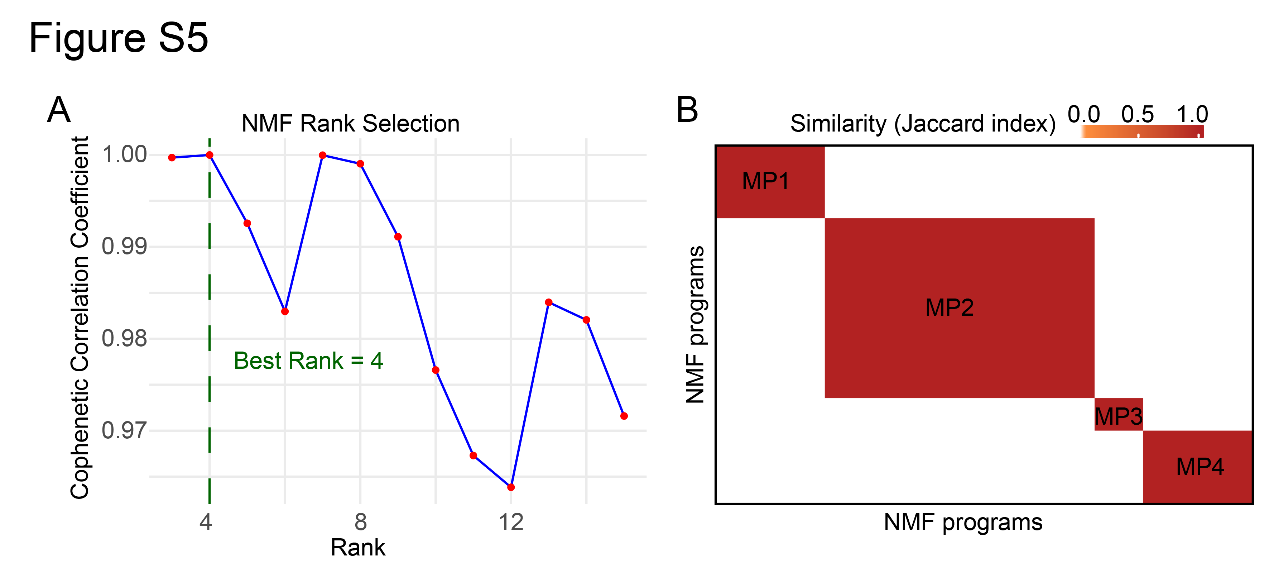


**Figure S5. Non-negative matrix factorization (NMF) analysis of G422^TN^-GBM cells from integrated scRNA-seq and snRNA-seq data, related to Figure 1.**

(A) Cophenetic correlation coefficients across different ranks for non-negative matrix factorization (NMF) analysis, with the optimal rank determined at 4 (green dashed line).

(B) Heatmap showing the pairwise similarity (Jaccard index) among the four NMF-derived meta-programs (MP1–MP4).


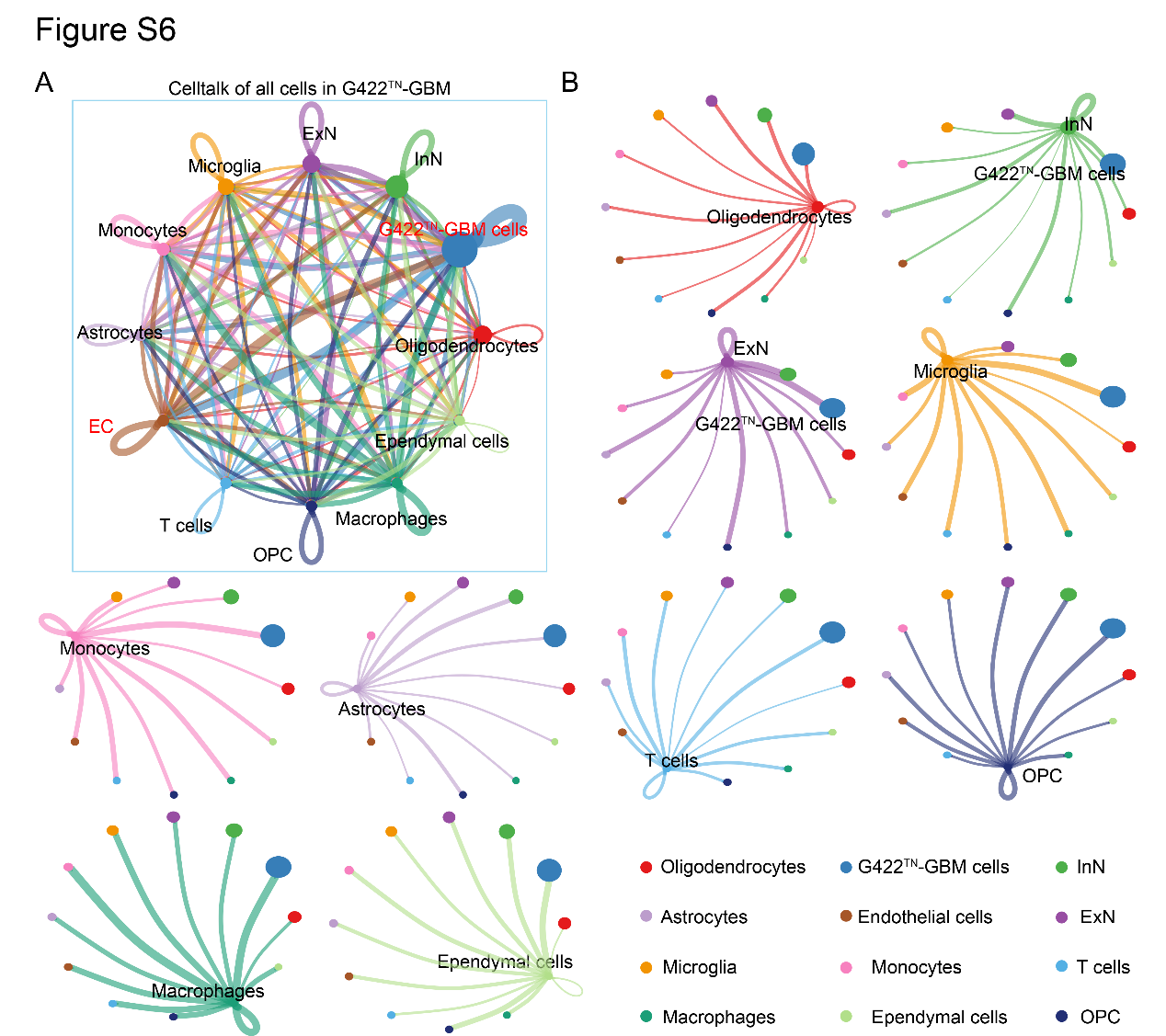


**Figure S6. Cell–cell communication analysis of the G422^TN^-GBM model using integrated scRNA-seq and snRNA-seq data, related to Figure 1.**

(A) Global cell–cell interaction network of all major cell types in the G422^TN^-GBM model inferred by CellChat. The size of each node represents the relative number of cells, and the thickness of the edges indicates the strength of intercellular communication. EC, endothelial cells.

(B) Cell-type-specific interaction networks showing outgoing signaling connections from each cell population, highlighting the distinct communication patterns of G422^TN^-GBM cells and other stromal and immune cell types.


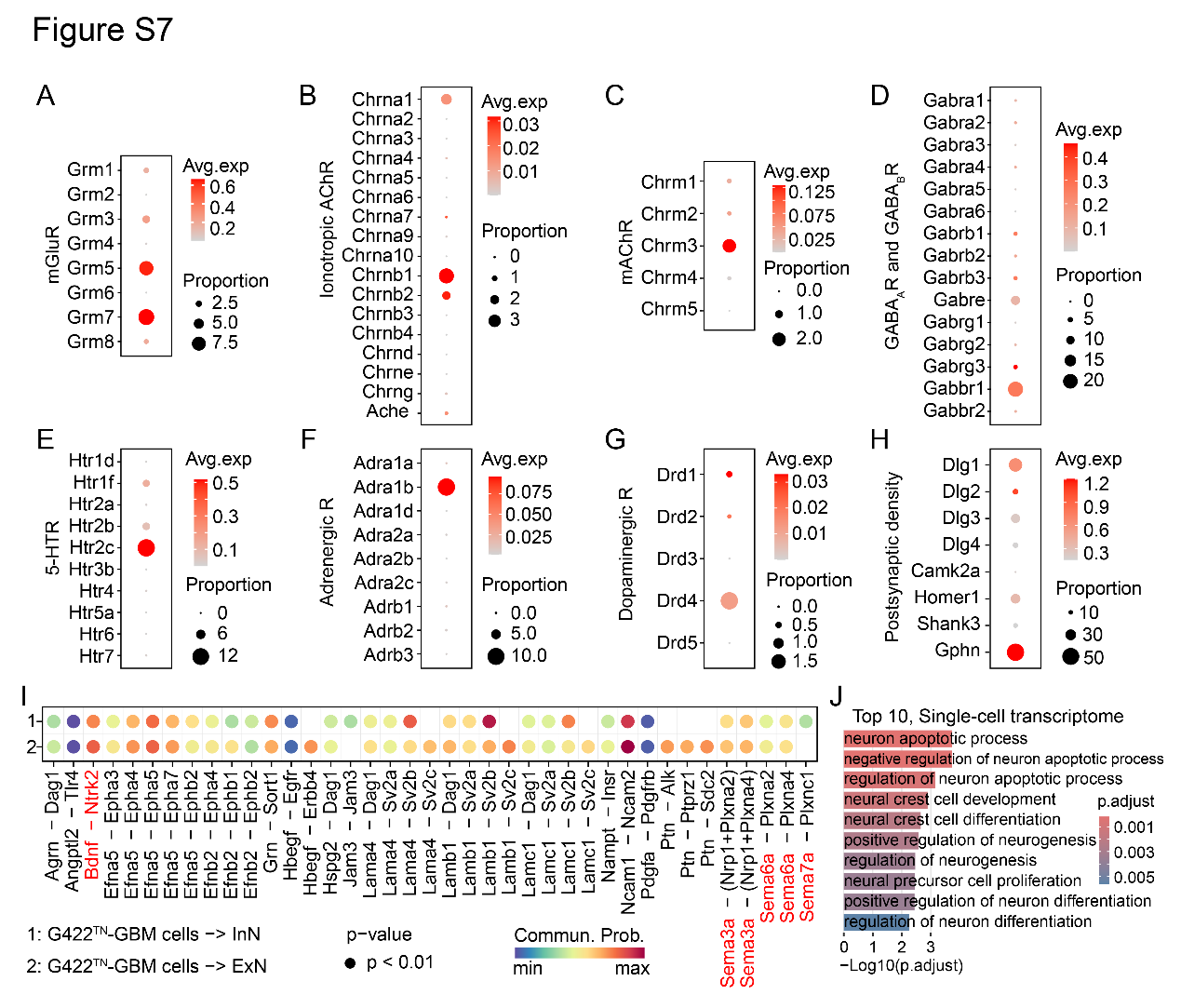


**Figure S7. Crosstalk analysis between G422^TN^-GBM cells and neurons using integrated scRNA-seq and snRNA-seq data, related to Figure 1.**

(A-H) Dot plots showing the expression of neurotransmitter receptors, including mGluR (A), ionotropic AChR (B), mAChR (C), GABA_A_R and GABA_B_R (D), 5-HTR (E), Adrenergic R (F), Dopaminergic R (G) and Postsynaptic density (H) in G422^TN^-GBM cells. Data are plotted as log-normalized counts, and the dot size represents the proportion of cells with the given gene detected. mGluR, metabotropic glutamatergic receptors; mAChR, muscarinic acetylcholine receptors. Avg.exp, average expression.

(I) Bubble plot showing significant ligand-receptor pairs mediating communication from G422^TN^-GBM cells to inhibitory neurons (InN, 1) and excitatory neurons (ExN, 2). The size of each bubble represents the significance of the interaction (p-value < 0.01), and the color intensity indicates the communication probability (from min to max).

(J) Top 10 GO enrichment pathways of G422^TN^-GBM cell–specific genes identified from integrated scRNA-seq and snRNA-seq data.


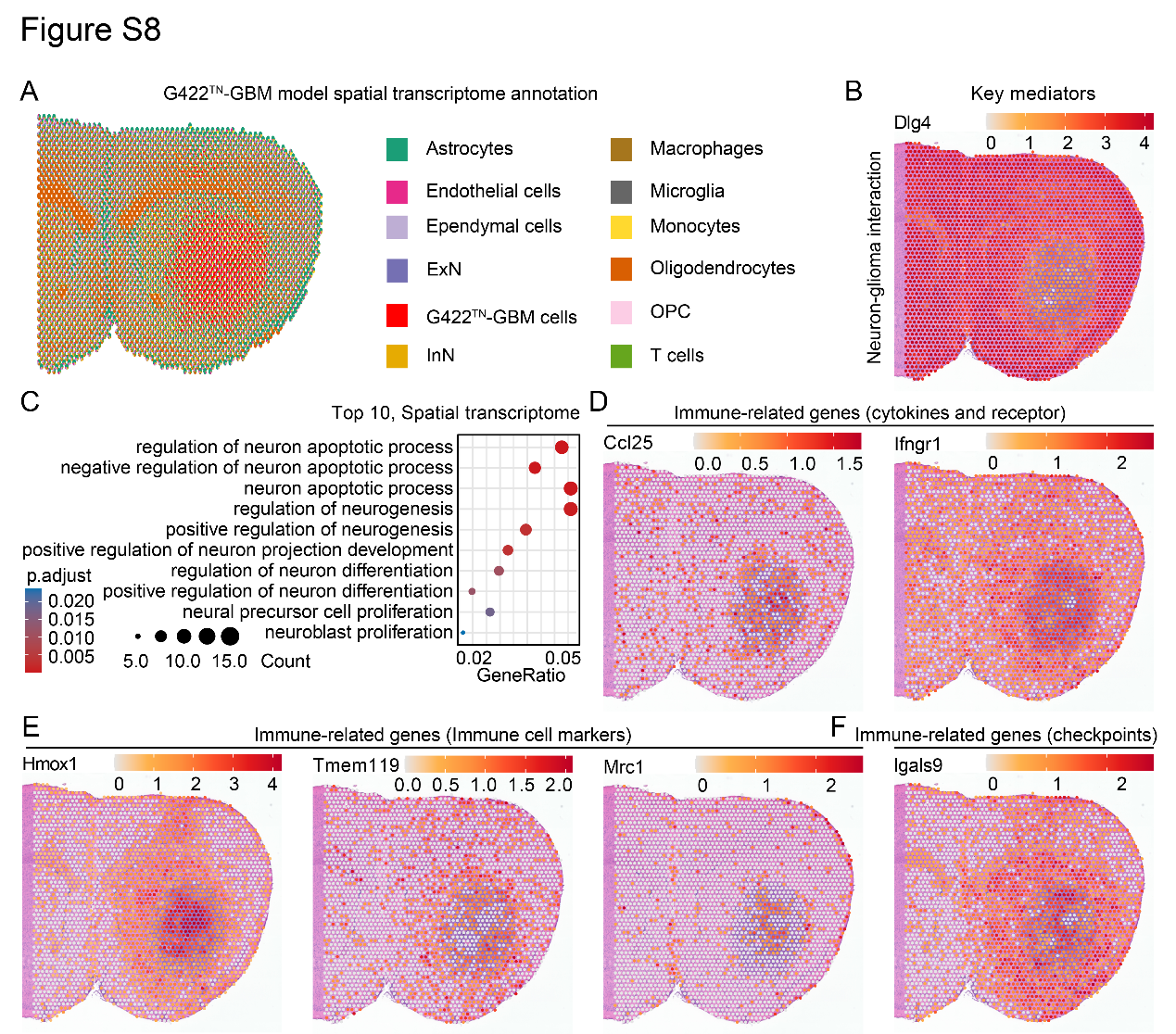


**Figure S8.** **Spatial transcriptomic analysis of the G422^TN^-GBM model, related to Figure 1.**

(A) Deconvolution analysis of spatial transcriptomic data using the CARD package based on the integrated scRNA-seq and snRNA-seq data of the G422^TN^-GBM model to infer the cellular composition of each spot.

(B) Spatial transcriptomics surface plots depicting regional expression of the neuron–glioma interaction mediator *Dlg4* in the G422^TN^-GBM model.

(C) Top 10 GO enrichment pathways of G422^TN^-GBM cell–specific genes identified from spatial transcriptomics data.

(D-F) Spatial transcriptomics surface plots showing regional expression of cytokine/receptor genes (*Ccl25*, *Ifngr1*), immune cell markers (*Hmox1*, *Tmem119*, *Mrc1*), and the checkpoint gene (*Lgals9*) in the G422^TN^-GBM model.


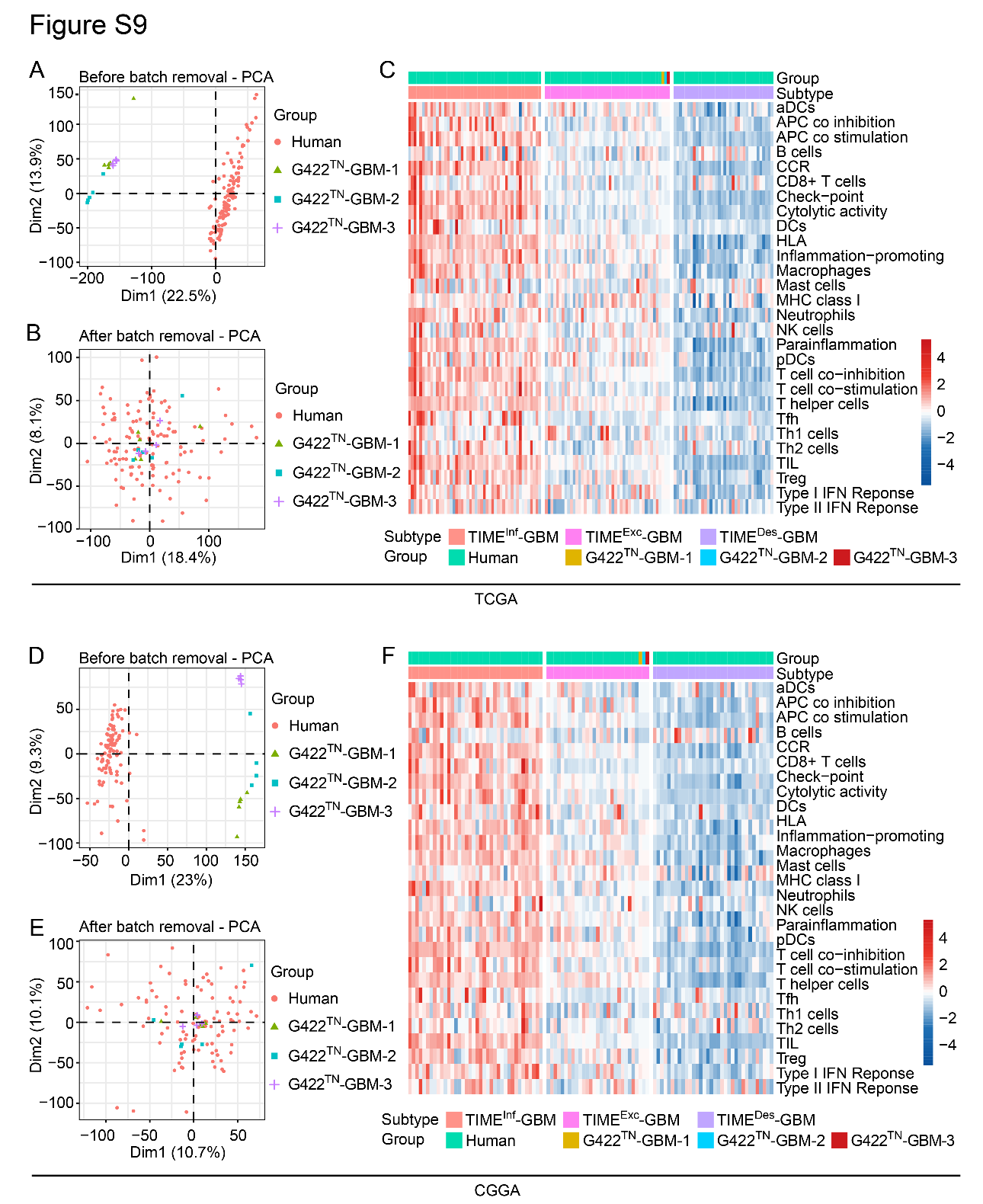


**Figure S9. Integrative clustering analysis of three batches of bulk RNA-seq data from the G422^TN^-GBM model with GBM patient datasets from TCGA and CGGA, related to Figure 1.**

(A-B) PCA plots showing overall variability of RNA-seq data from four batches: the TCGA cohort (n = 126) and G422^TN^-GBM model samples from three independent experiments (n = 14). Uncorrected data (A) and batch-corrected data (B) from ComBat are shown.

(C) Heatmap showing three TIME subtypes of GBM identified by partition around medoids (PAM) clustering, based on the enrichment scores of 23 immune cell types calculated by ssGSEA. The analysis was performed on the batch-corrected data from (B). TIME, tumor immune microenvironment.

(D-E) PCA plots showing overall variability of RNA-seq data from four batches: the CGGA-GBM cohort (n=98) and G422^TN^-GBM model samples from three independent experiments. Uncorrected data (D) and batch-corrected data (E) from ComBat are shown.

(F) Heatmap showing three TIME subtypes of GBM identified by PAM clustering, based on the enrichment scores of 23 immune cell types calculated by ssGSEA. The analysis was performed on the batch-corrected data from (E). RNA-seq data from each of the three independent G422^TN^-GBM experiments were averaged separately in panels (C) and (F).


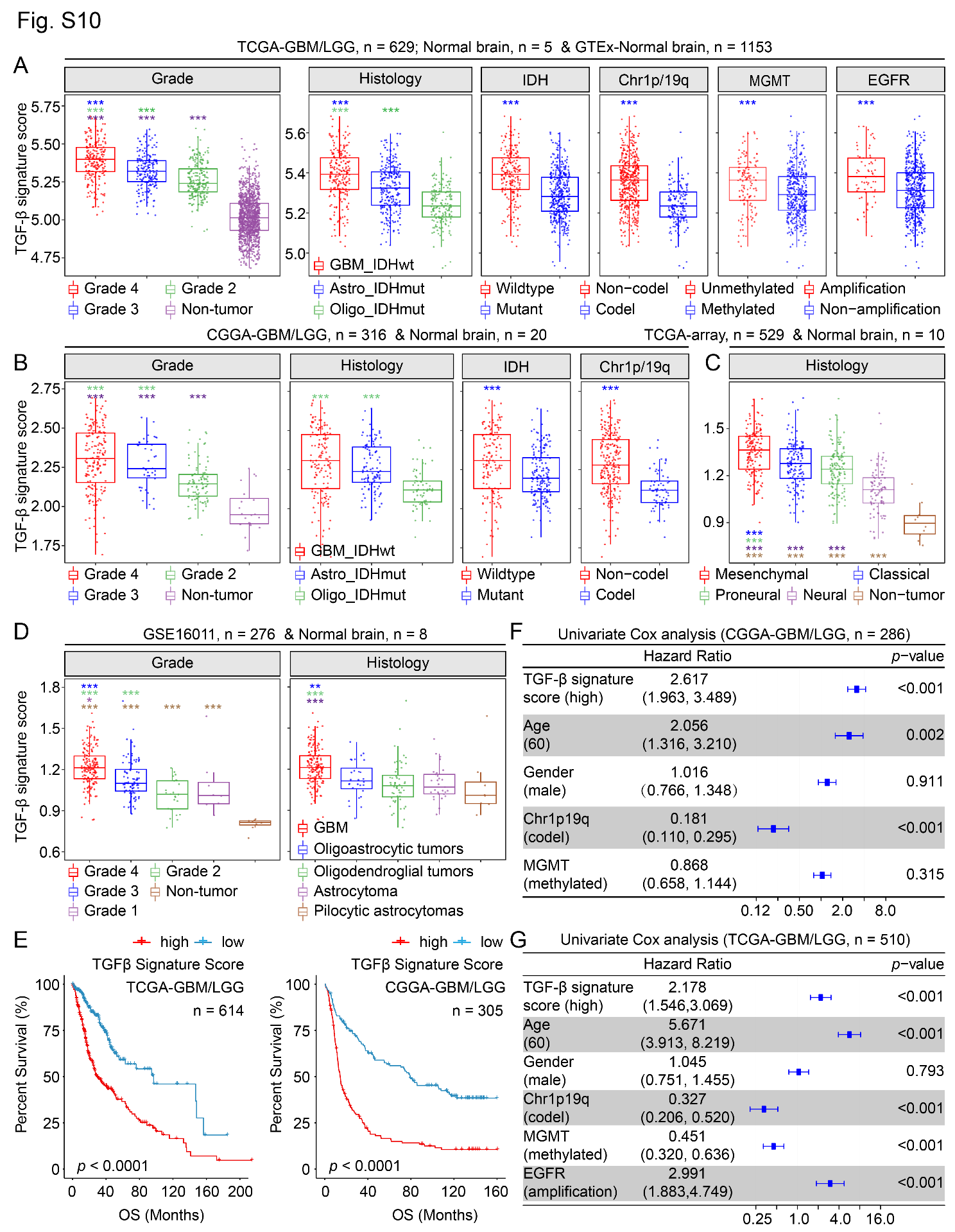


**Figure S10. Prognostic analysis of TGF-β signaling in glioma patients, related to Figure 2.**

(A-D) Boxplots showing ssGSEA-derived TGF-β signature scores across normal brains and gliomas by grade, histology, or molecular subtype in TCGA/GTEx (A, n=1787), CGGA (B, n=336), TCGA-array (C, n=539), and GSE16011 (D, n=284) cohorts. Center line shows median, box limits indicate upper and lower quartiles, and whiskers extend 1.5 times the interquartile range.

(E) Overall survival of glioma patients from TCGA (left panel, n=641) and CGGA (right panel, n=313) cohorts, stratified into high and low groups based on the median TGF-β signature score.

(F-G) Univariate Cox regression analysis of the overall survival of patients using CGGA (n = 286 cases) (F) and TCGA (n = 510 cases) (G) cohorts respectively. Forest plots with error bars show 2.5% (lower) and 97.5% (higher) bounds of the confidence interval.

Statistical analysis, one-way ANOVA followed by Tukey’s post hoc test (Grade and Histology in A, B, C and D), two-tailed unpaired Student’s t test (IDH, Chr1p/19q, MGMT and EGFR in A and B). Survival curves were analyzed using a log-rank (Mantel-Cox) test (E). Error bars, mean ± SEM. ^*^*p* < 0.05; ^**^*p* < 0.01; ^***^*p* < 0.001.


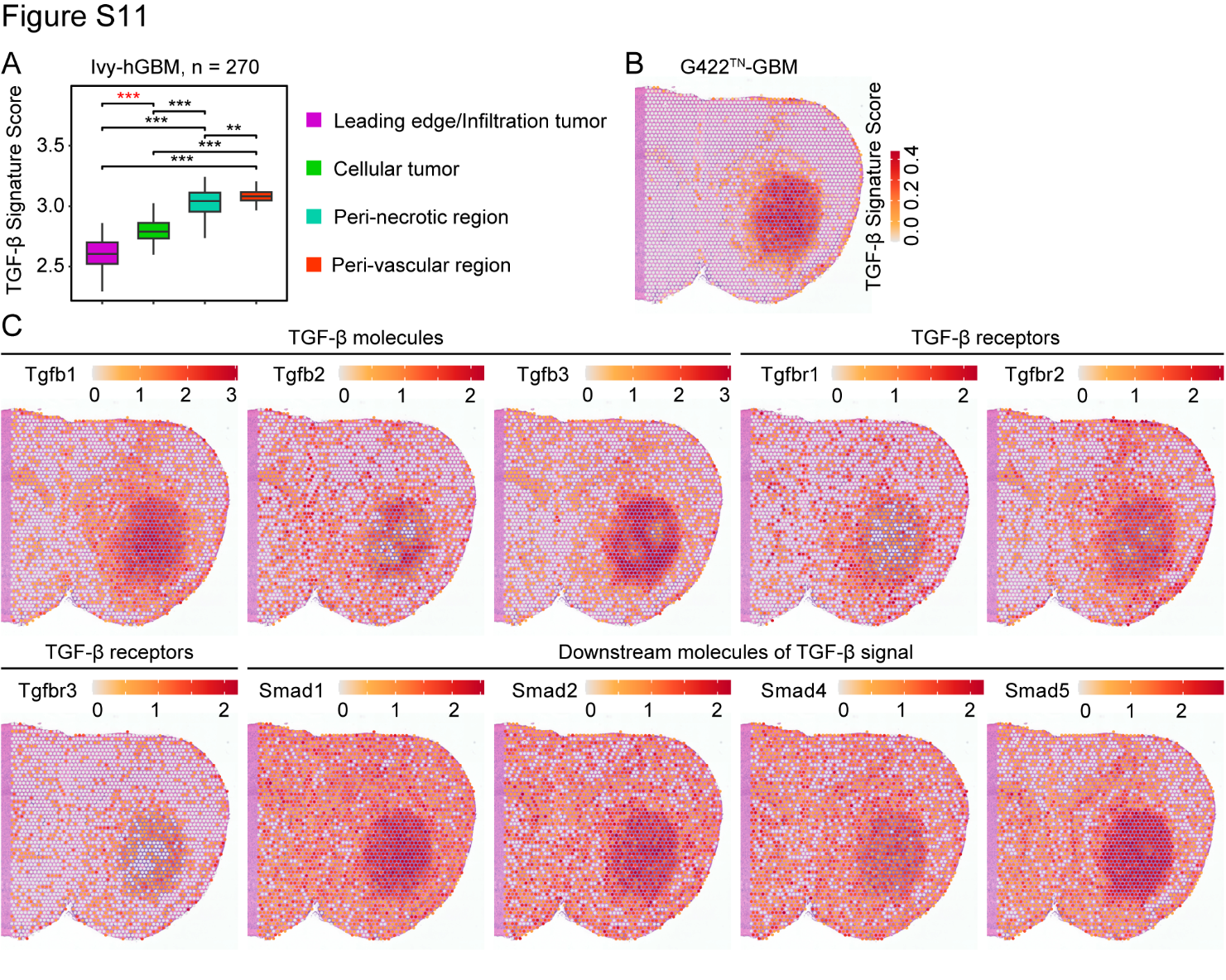


**Figure S11. Spatial distribution of TGF-β signaling signature score and related molecules in the Ivy-hGBM dataset and G422^TN^-GBM model, related to Figure 2.**

(A) Boxplots showing the estimated TGF-β signature score across different histological regions of hGBMs using bulk RNA-seq data from the Ivy-hGBM cohort (n = 270 cases).

(B) Surface plots showing the estimated TGF-β signature score in spatial transcriptomics data of our G422^TN^-GBM model.

(C) Spatial transcriptomics surface plots showing regional expression of TGF-β ligands (Tgfb1, Tgfb2, Tgfb3), receptors (Tgfbr1, Tgfbr2, Tgfbr3), and downstream effectors (Tgfbi, Tgfb1i1, Smad1, Smad2, Smad4, Smad5) in the G422^TN^-GBM model.

Statistical analysis, one-way ANOVA followed by Tukey’s post hoc test (A). Error bars, mean ± SEM. ^**^*p* < 0.01; ^***^*p* < 0.001,


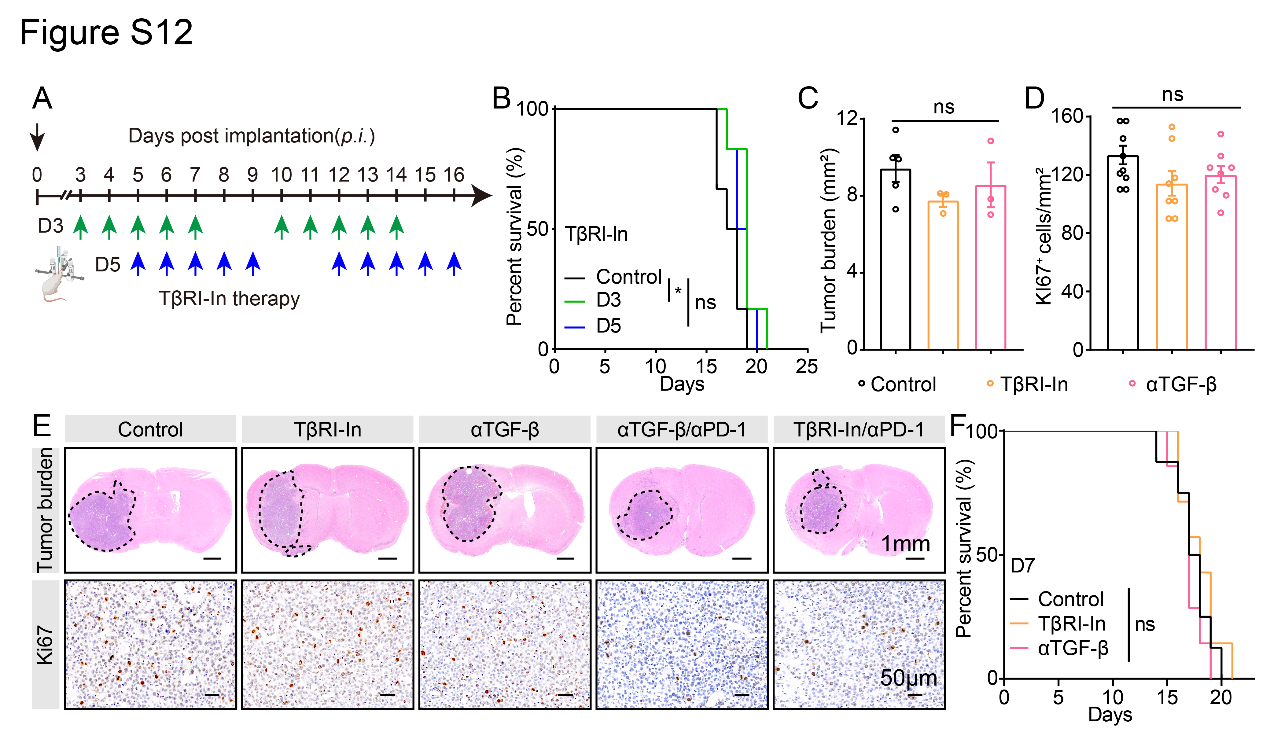


**Figure S12. Therapeutic effects of TGF-β signaling blockade in the G422^TN^-GBM model, related to Figure 2.**

(A) Schematic illustration depicting the treatment regimens with TβRI-In initiated on day 3 or 5 *p.i.*. TβRI-In, TGF-β receptor I inhibitor, Galunisertib, 50 mg/kg, ig, twice daily.

(B) Survival curve of the G422^TN^-GBM mice with TβRI-In treatment initiated on day 3 or 5 *p.i.* and control group (n = 7-8/group).

(C-D) Quantitative analyses of tumor burden (C, n = 3-5) and Ki67 IHC staining (D, n = 8–10) in the G422^TN^-GBM mice with TβRI-In treatment initiated on day 7 *p.i.* and control group.

(E) Representative H&E staining for tumor burden and IHC staining for Ki67 in G422^TN^-GBM tumors from Control, TβRI-In, αTGF-β, TβRI-In/αPD-1 and αTGF-β/αPD-1 groups on day 9 *p.i.*.

(F) Survival curve of the G422^TN^-GBM mice with TβRI-In and αTGF-β treatment initiated on day 7 *p.i.* and control group (n = 7-8/group).

Survival curves were analyzed using a log-rank (Mantel-Cox) test (B and F). Statistical analysis, one-way ANOVA followed by Tukey’s post hoc test (C and D). Error bars, mean ± SEM. ^*^*p* < 0.05; ns, not significant.


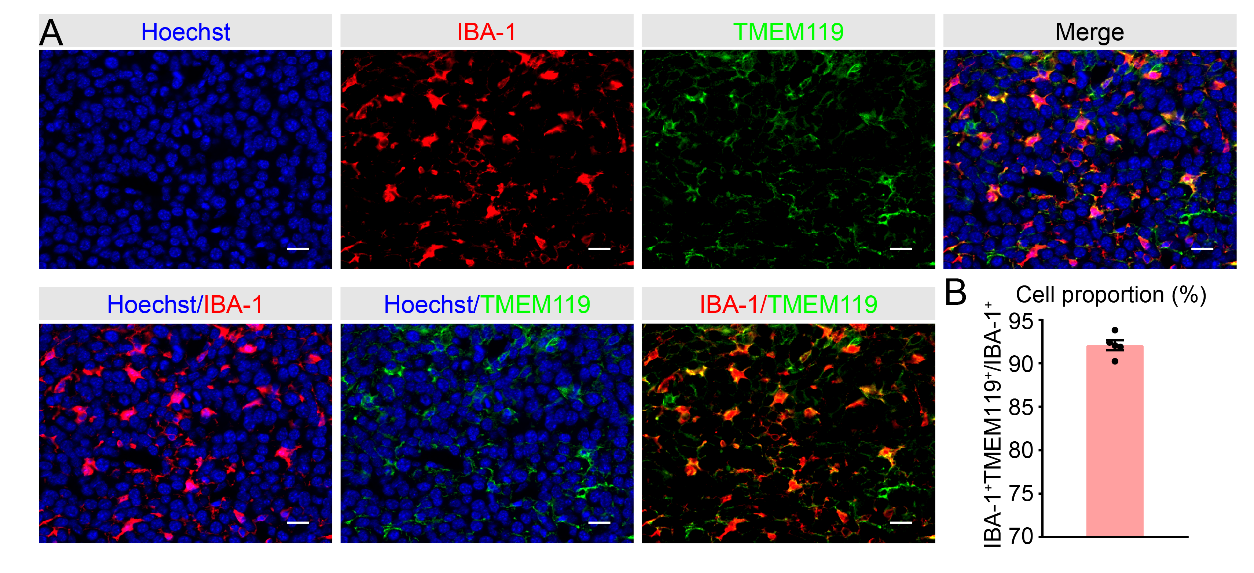


**Figure S13. Colocalization of TMEM119 and IBA-1 in the G422^TN^-GBM TME, related to Figure 2.**

(A) Representative immunofluorescence images of IBA-1 and TMEM119 double staining. (B) Percentage of IBA-1⁺ TMEM119⁺ cells among IBA-1⁺ cells.


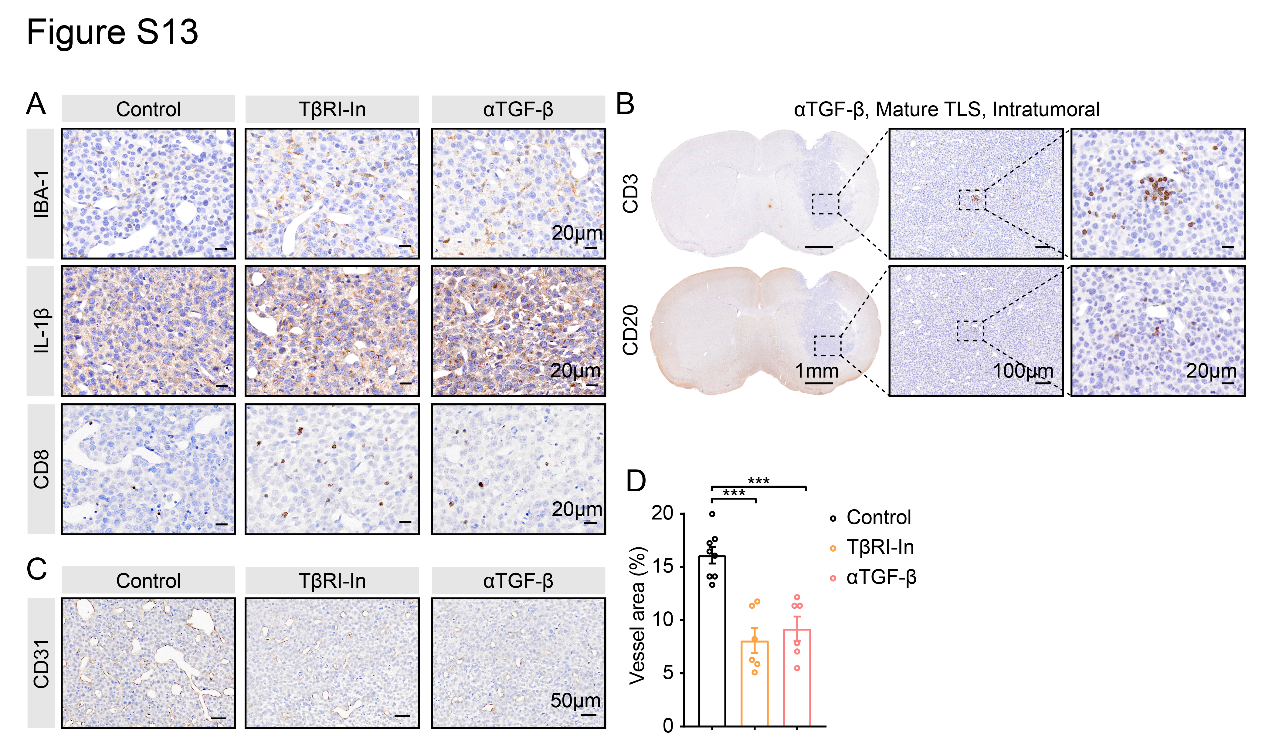


**Figure S14. Alterations in TME–related molecules following TGF-β signaling blockade in the G422^TN^-GBM model, related to Figure 2.**

(A) Representative IHC staining of IBA-1, IL-1β and CD8 in G422^TN^-GBM tumors from Control, TβRI-In, and αTGF-β groups on day 9 *p.i.*.

(B) Representative images of intratumoral TLS in the αTGF-β group detected by sequential whole-brain sections with IHC staining of CD3 and CD20.

(C-D) Representative IHC images (C) and quantitative analyses (D) of CD31 staining in the G422^TN^-GBM tumors from Control, TβRI-In, and αTGF-β groups (n = 6-8/group).

Statistical analysis, one-way ANOVA followed by Tukey’s post hoc test (D). Error bars, mean ± SEM. ^***^*p* < 0.001.


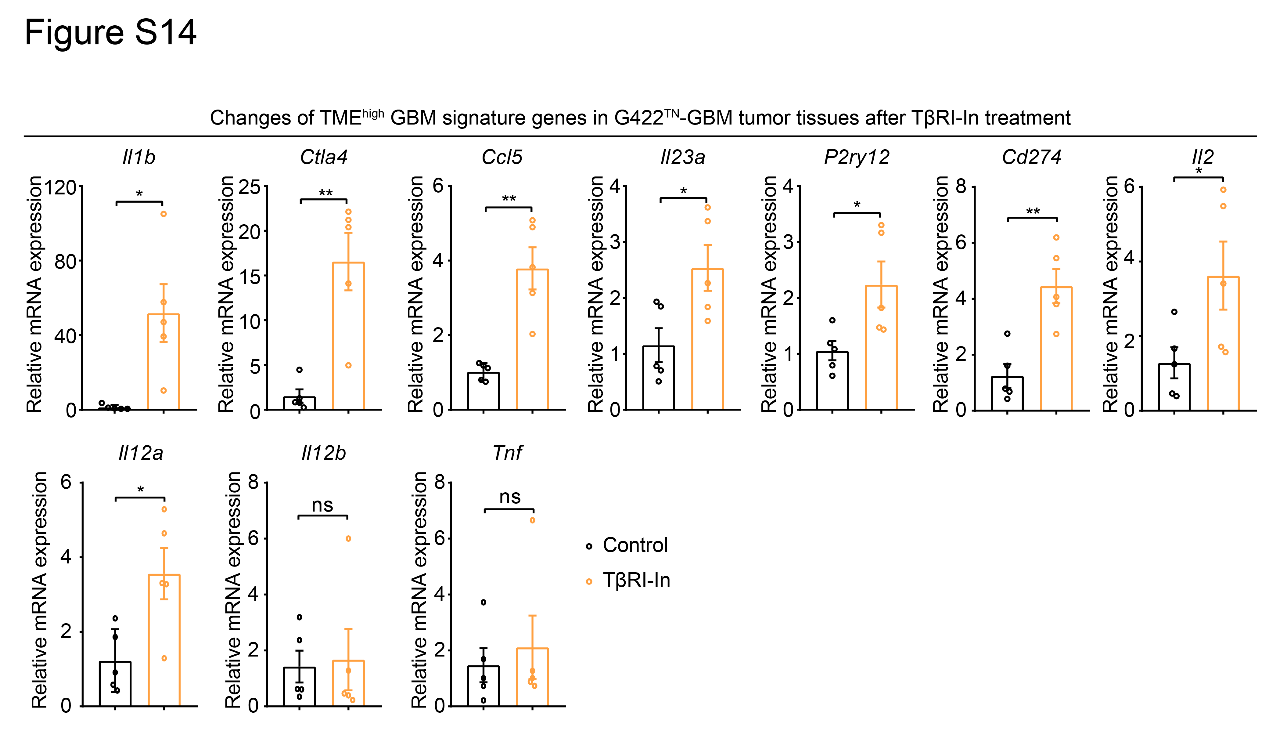


**Figure S15. Alterations in TME^High^ GBM signature genes following TGF-β signaling blockade in the G422^TN^-GBM model, related to Figure 2.**

RT-qPCR analysis showing increased *Il1b*, *Ctla4*, *Ccl5*, *Il23a*, *P2ry12*, *Cd274*, *Il12*, and *Il12a* mRNA levels, with unchanged *Il12b*, *Tnf* expression, in TβRI-In–treated G422^TN^-GBM tumors compared with controls (n = 5/group).

Statistical analysis, two-tailed unpaired Student’s t test. Error bars, mean ± SEM. ^*^*p* < 0.05; ^**^*p* < 0.01; ns, not significant.


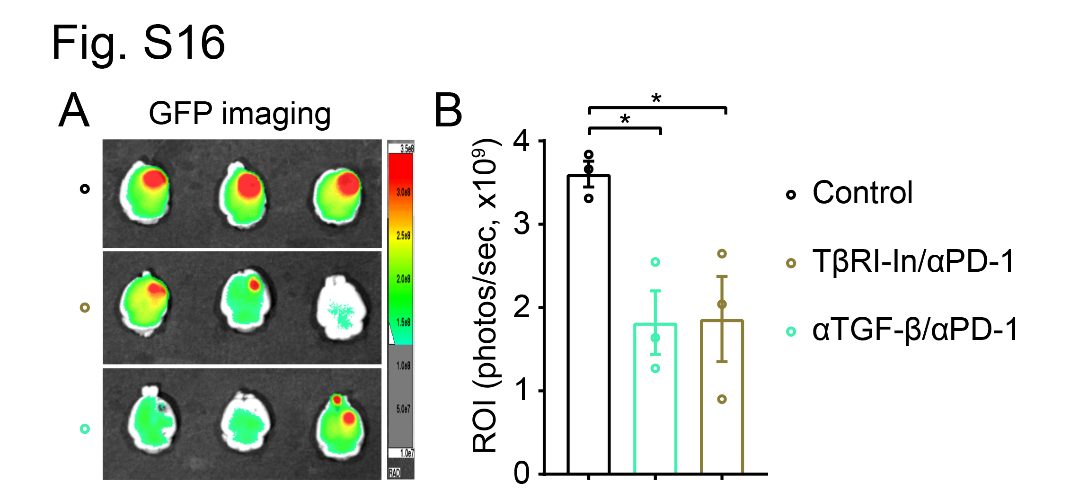


**Figure S16. Whole-brain GFP imaging showing the therapeutic effects of combined TGF-β signaling and αPD-1 blockade in the G422TN-GBM model, related to Figure 2.**

(A-B) Representative GFP images (A) and quantitative analyses of ROI values (B) for intracranial G422^TN^-GBM tumors in the Control, TβRI-In/αPD-1, and αTGF-β/αPD-1 groups (n = 3/group).

Statistical analysis, one-way ANOVA followed by Tukey’s post hoc test (B). Error bars, mean ± SEM. ^*^*p* < 0.05.


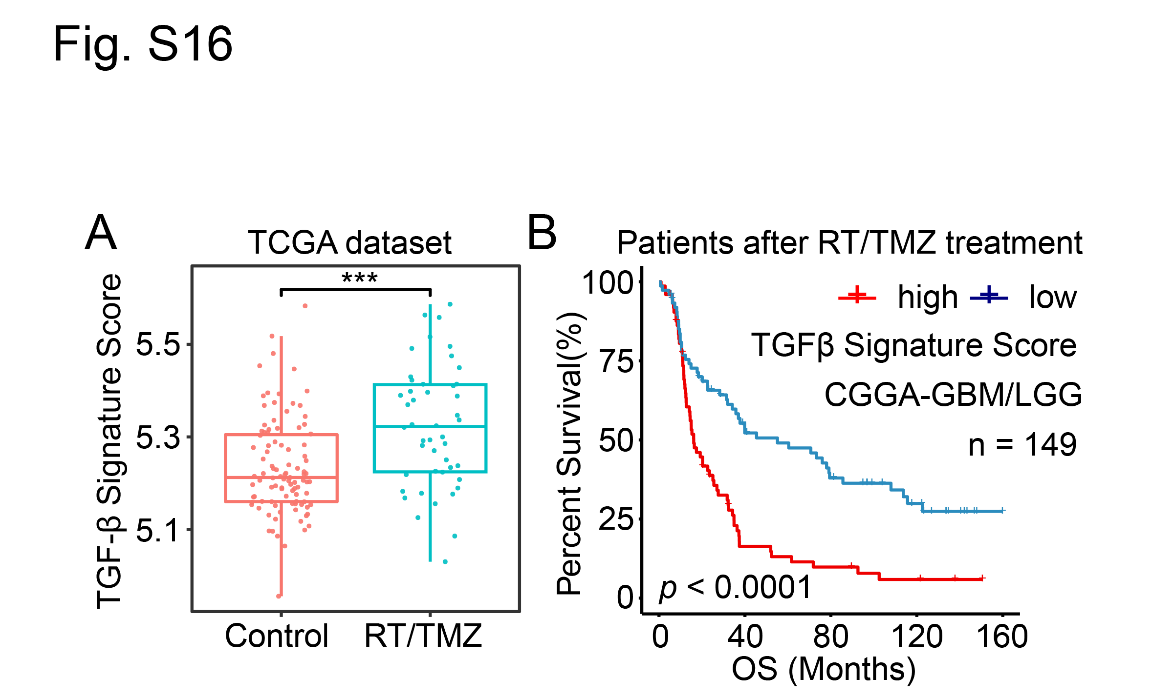


**Figure S17. Prognostic analysis of TGF-β signaling in glioma patients following RT/TMZ treatment, related to Figure 2.**

(A) Boxplots showing estimated TGF-β signature scores across different groups (RT/TMZ vs. Control) in glioma patients based on bulk RNA-seq data from the TCGA cohort (n = 145 cases).

(B) Overall survival of glioma patients following RT/TMZ treatment from TCGA (left, n=145) and CGGA (right, n=111) cohorts, stratified into high and low groups according to the median TGF-β signature score.

Statistical analysis, two-tailed unpaired Student’s t test (A). Survival curves were analyzed using a log-rank (Mantel-Cox) test (B). Error bars, mean ± SEM. ^***^*p* < 0.001.


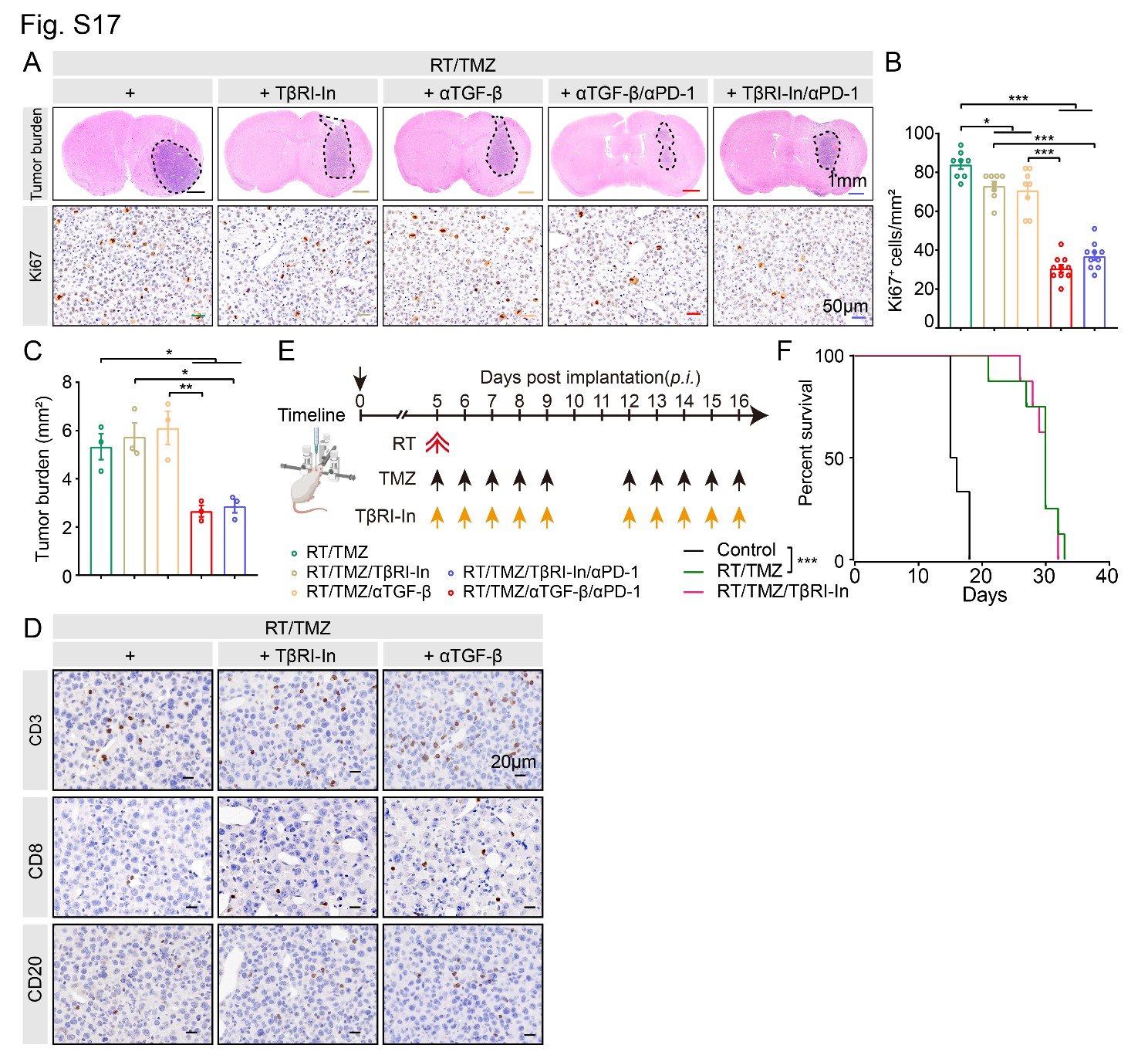


**Figure S18. Therapeutic effects of TGF-β signaling blockade in the G422^TN^-GBM model following RT/TMZ treatment, related to Figure 2.**

(A) Representative H&E staining for tumor burden and IHC staining for Ki67 in G422^TN^-GBM tumors from RT/TMZ, RT/TMZ/TβRI-In, RT/TMZ/αTGF-β, RT/TMZ /TβRI-In/αPD-1 and RT/TMZ/αTGF-β/αPD-1 groups on day 9 *p.i.*.

(B) Quantitative analysis of Ki67 IHC staining in G422^TN^-GBM mice treated with RT/TMZ, RT/TMZ/TβRI-In, RT/TMZ/αTGF-β, RT/TMZ/TβRI-In/αPD-1, or RT/TMZ/αTGF-β/αPD-1, with treatments initiated on day 7 p.i. (n = 8–10).

(C) Statistical analyses of tumor burden in G422^TN^-GBM tumors from RT/RMZ, RT/TMZ/TβRI-In, RT/TMZ/αTGF-β, RT/TMZ/TβRI-In/αPD-1 and RT/TMZ/αTGF-β/αPD-1 groups (n = 3/group).

(D) Representative IHC images of CD3, CD8 and CD20 in G422^TN^-GBM tumors from RT/TMZ, RT/TMZ/TβRI-In, and RT/TMZ/αTGF-β treatment groups on day 9 *p.i.*.

(E) Schematic diagram depicting the treatment regimens with RT, TMZ, TβRI-In, or their combinations initiated on day 5 *p.i.*. RT, a single dose of 10 Gy whole-brain irradiation (WBI); TMZ, 10 doses of temozolomide (50 mg/kg, ig); TβRI-In, 50 mg/kg, ig, twice daily.

(F) Survival curves of G422^TN^-GBM mice treated with RT/TMZ or RT/TMZ/TβRI-In, with treatments initiated on day 5 *p.i.*, and the control group (n = 7–8 per group).

Statistical analysis, one-way ANOVA followed by Tukey’s post hoc test (B and C). Survival curves were analyzed using a log-rank (Mantel-Cox) test (F). Error bars, mean ± SEM.  ^*^*p* < 0.05; ^***^*p* < 0.001.


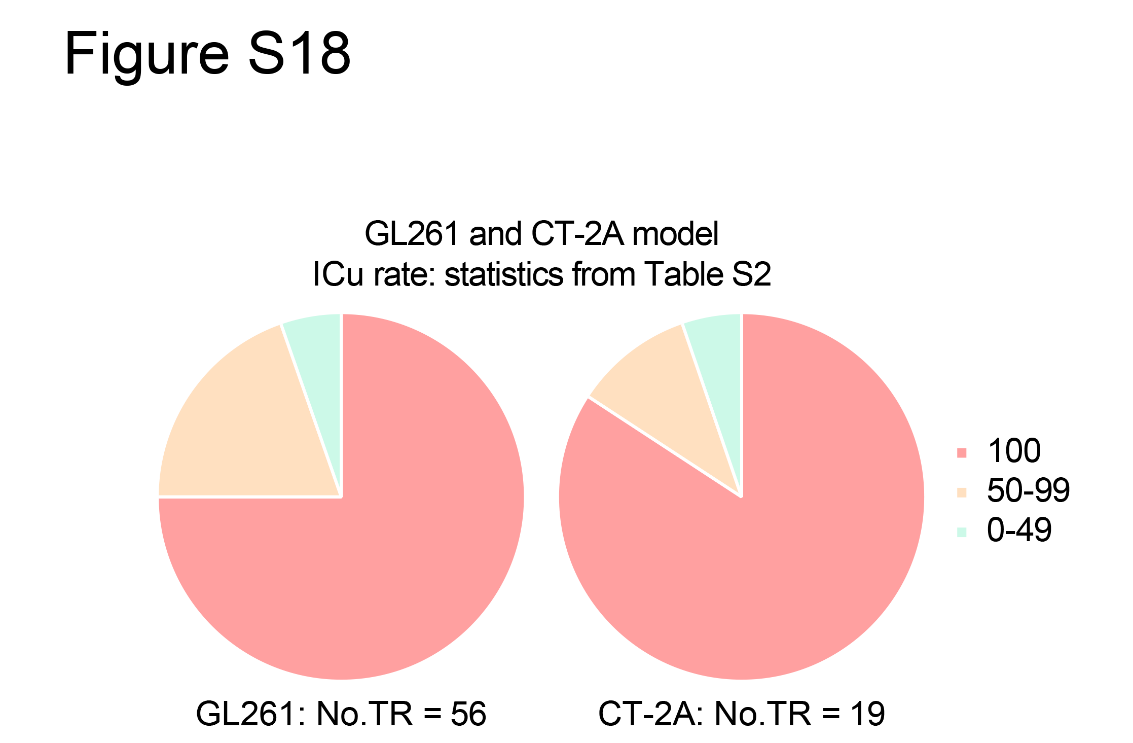


**Figure S19. Proportion of ICu mice among LTS mice in the GL261 and CT-2A models, related to Figure 3.**

Statistical analysis of the ICu rate, i.e., the proportion of cured mice in commonly used GL261 and CT-2A models that successfully resisted tumor rechallenge. Literature was searched using combined keywords including glioma, tumor rechallenge, and antitumor immune memory, while excluding studies involving rat models, non-adult diffuse glioma models, subcutaneous models, and brain metastasis models. Data collection was up to July 31, 2025. No.TR, number of tumor rechallenge assays.


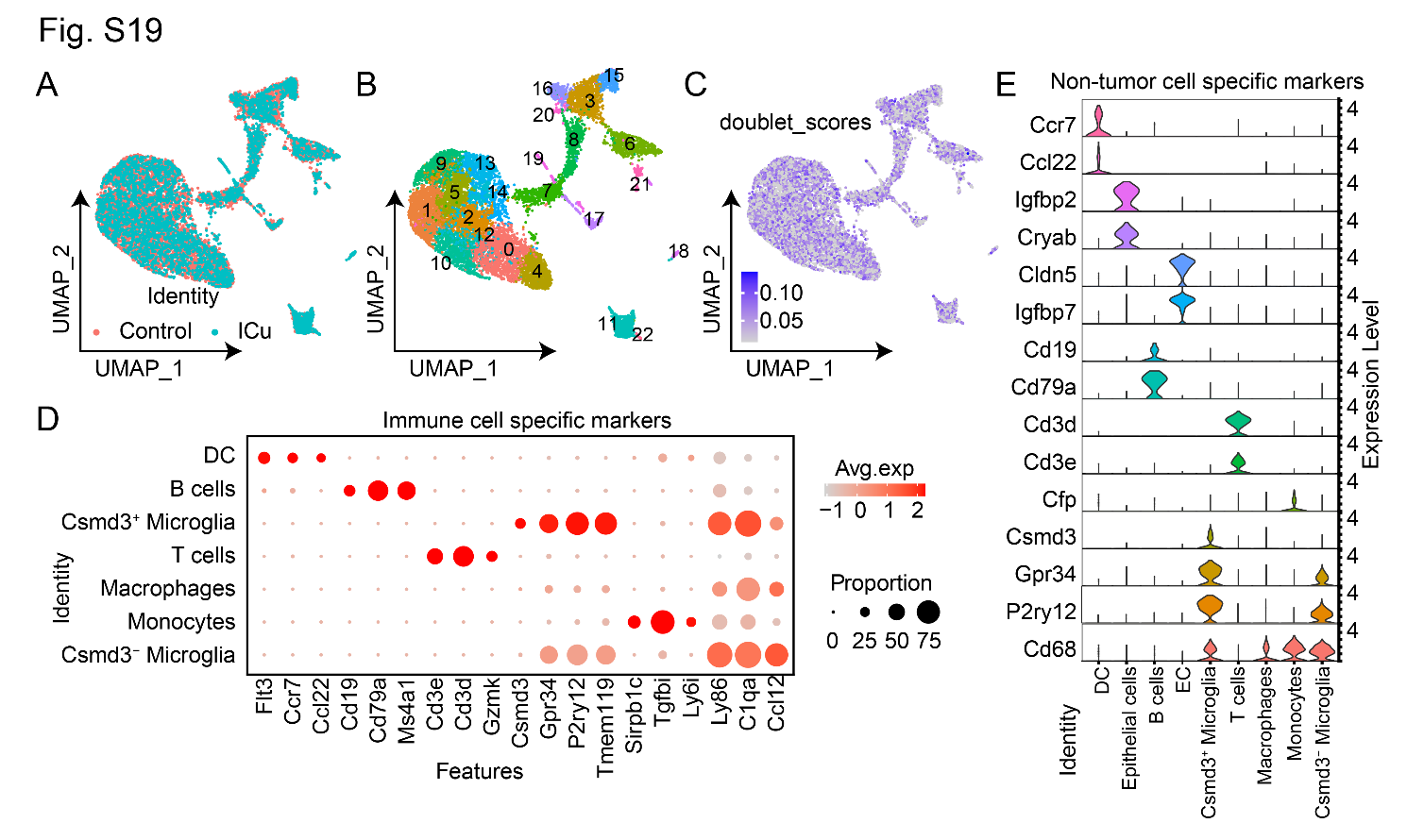


**Figure S20. Integration of scRNA-seq data** **from ICu and control mice, related to Figure 3.**

(A) UMAP plot depicting the integration of scRNA-seq data from ICu and control mice using the Harmony algorithm.

(B) UMAP plot showing all cell clusters in the integration of scRNA-seq data from ICu and control mice.

(C) UMAP plot showing doublet scores of the integration of scRNA-seq data from ICu and control mice, calculated using Scrublet (Python package).

(D) Dot plots showing the expression of specific marker genes across all immune cell types in the integration of scRNA-seq data from ICu and control mice. Data are plotted as log-normalized counts, and the dot size represents the proportion of cells with the given gene detected.  DC, dendritic cells. Avg.exp, average expression.

(E) Violin plots showing the expression of specific marker genes across all non-tumor cell types in the integration of scRNA-seq data from ICu and control mice.


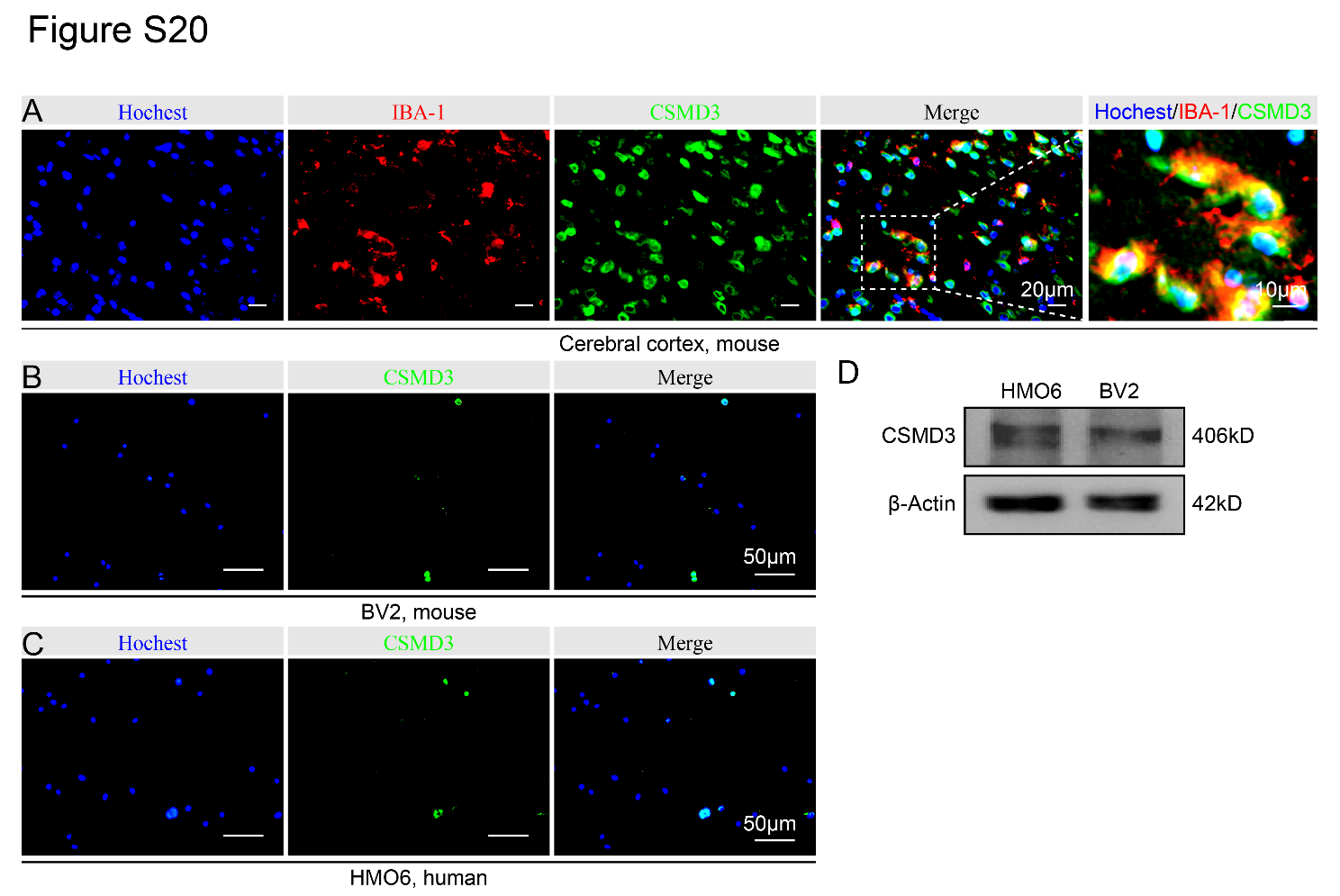


**Figure S21.** **IF staining and WB analysis showing CSMD3 expression in microglia, related to Figure 3.**

(A) Representative IF images of IBA1 (microglia marker) and CSMD3 staining in the mouse cerebral cortex.

(B) Representative IF images of CSMD3 staining in mouse BV2 microglia cells.

(C) Representative IF images of CSMD3 staining in human HMO6 microglia cells.

(D) Representative WB images of CSMD3 protein in BV2 and HMO6 cells with β-Actin used as a loading control.


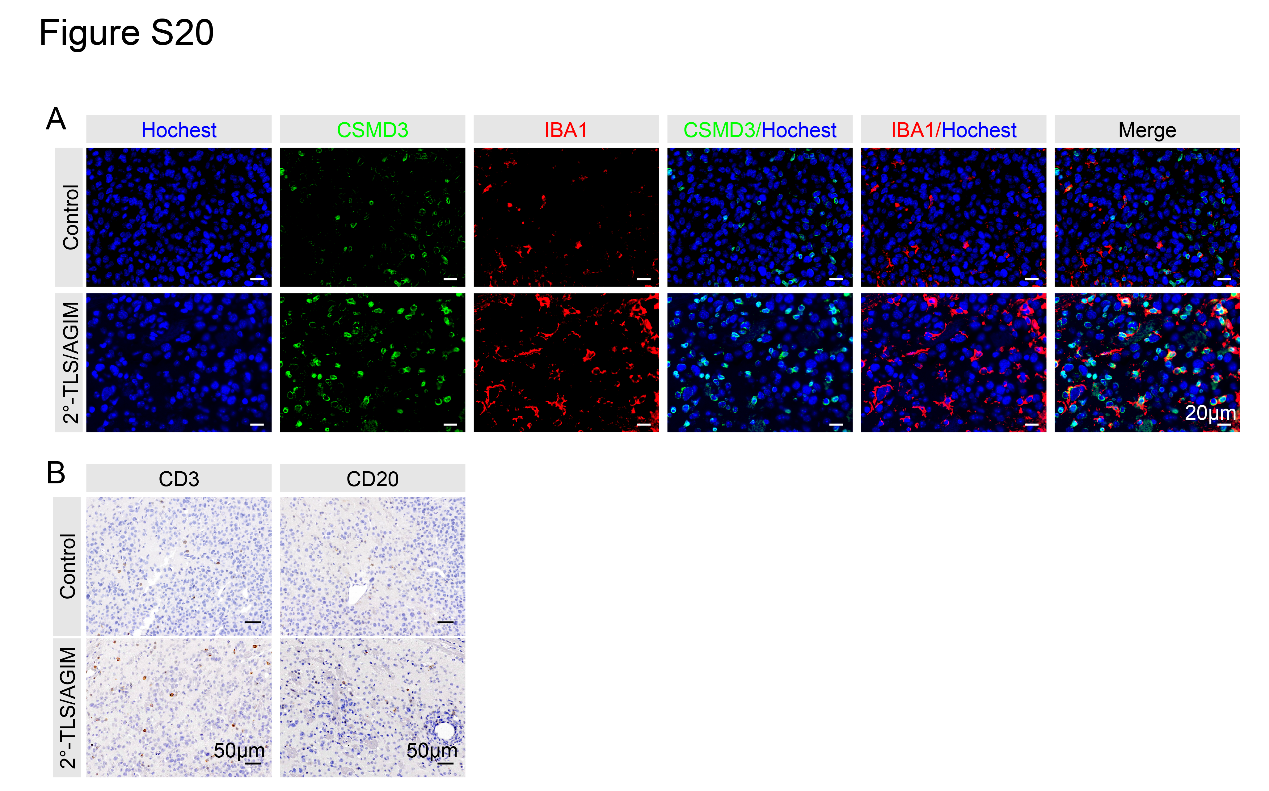


**Figure S22. Validation of increased CSMD3⁺ microglia, T cells, and B cells in** **ICu mice, related to Figure 3.**

(A) Representative IF images of IBA1 and CSMD3 staining in the TC region of ICu and control mice bearing G422^TN^-GBM tumors on day 7 *p.i.*.

(B) Representative IHC images of CD3 and CD20 staining in the TC region of ICu and control mice bearing G422^TN^-GBM tumors on day 7 *p.i.*.


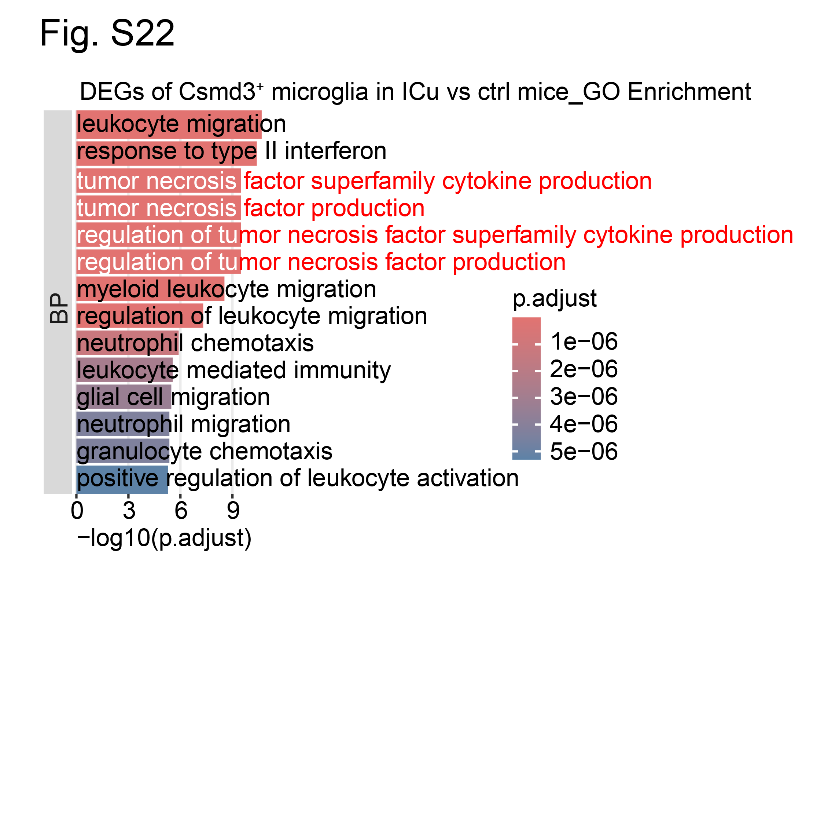


**Figure S23. Csmd3⁺ microglia exhibit a trained** **innate immune memory (IMM) phenotype in ICu mice, related to Figure 3.**

Top 14 GO-BP enrichment pathways of upregulated genes in Csmd3⁺ microglia from ICu versus control mice identified by GO enrichment analysis. Red-highlighted BP terms indicate strong activation of TNF-α signaling in Csmd3⁺ microglia of ICu mice, a hallmark of the trained IIM phenotype.


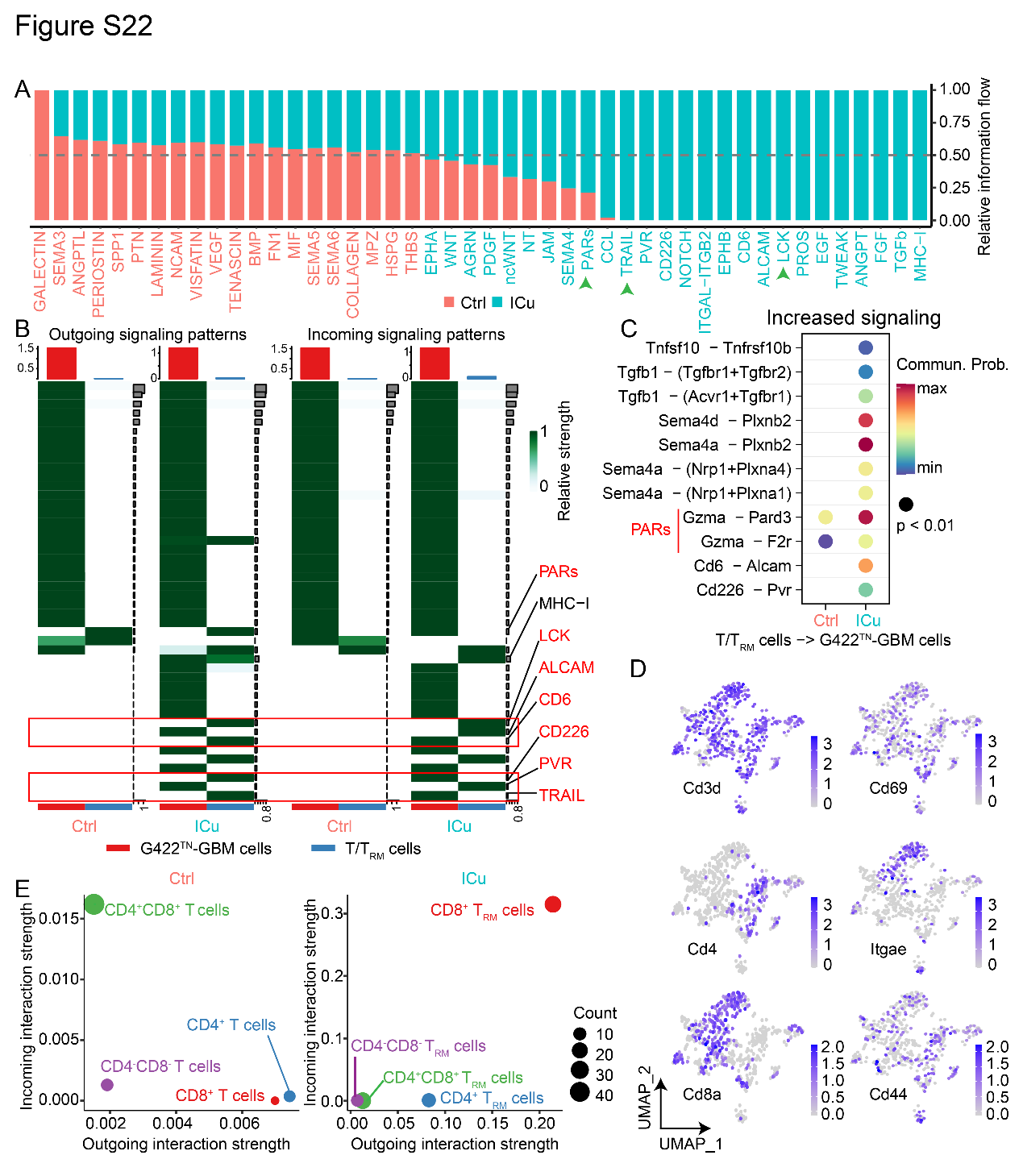


**Figure S24. Cell–cell communication between T/T_RM_ cells and G422^TN^-GBM cells and the subclusters of T/T_RM_ cells, related to Figure 3.**

(A) Signaling flow analysis of cell–cell communication showing pathway alterations between T–G422^TN^-GBM cell interactions in control mice and T_RM_–G422^TN^-GBM cell interactions in ICu mice.

(B) Outgoing and incoming signaling pattern analyses showing differential cell–cell communication strength between G422^TN^-GBM cells (red) and T/T_RM_ cells (blue) in control (Ctrl) and ICu (2°-LTS) mice. Each bar represents the relative contribution of specific ligand–receptor signaling pathways, with highlighted pathways (e.g., PARs, MHC-I, LCK, ALCAM, CD6, CD226, PVR, and TRAIL) indicating markedly enhanced immune-related interactions in the ICu group.

(C) Bubble plot showing the increased signaling probability of ligand–receptor interactions from T/T_RM_ cells to G422^TN^-GBM cells in ICu versus control (Ctrl) mice. Enhanced communication involves multiple pathways, including *PARs*, Cd6-Alcam, and *Cd226–Pvr* signaling, indicating strengthened immune–tumor interactions in the ICu group (*p* < 0.01).

(D) UMAP plots displaying representative expression patterns of T/T_RM_ cell markers (*Cd3d*, *Cd69*, *Cd4*, *Itgae*, *Cd8a*, and *Cd44*) in T/T_RM_ cell subsets derived from ICu and control mice.

(E) Scatter plot illustrating the major signaling senders (sources) and receivers (targets) among T/T_RM_ cell subsets in a two-dimensional space.


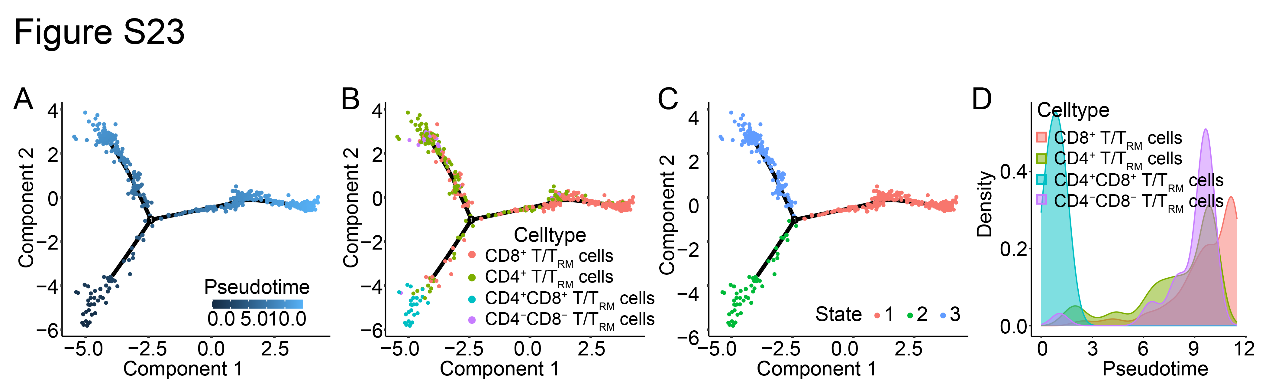


**Figure S25. Pseudotime analysis of T/T_RM_ cell subsets in ICu and control mice, related to Figure 3.**

(A-C) Trajectory plots of T/T_RM_ cells ordered along pseudotime (A), highlighting differentiation dynamics across subtypes (CD8⁺, CD4⁺, CD4⁺CD8⁺, CD4⁻CD8⁻) (B) and inferred cell states (State 1–3) (C).

(D) Density analysis highlights subtype-specific progression along the trajectory.


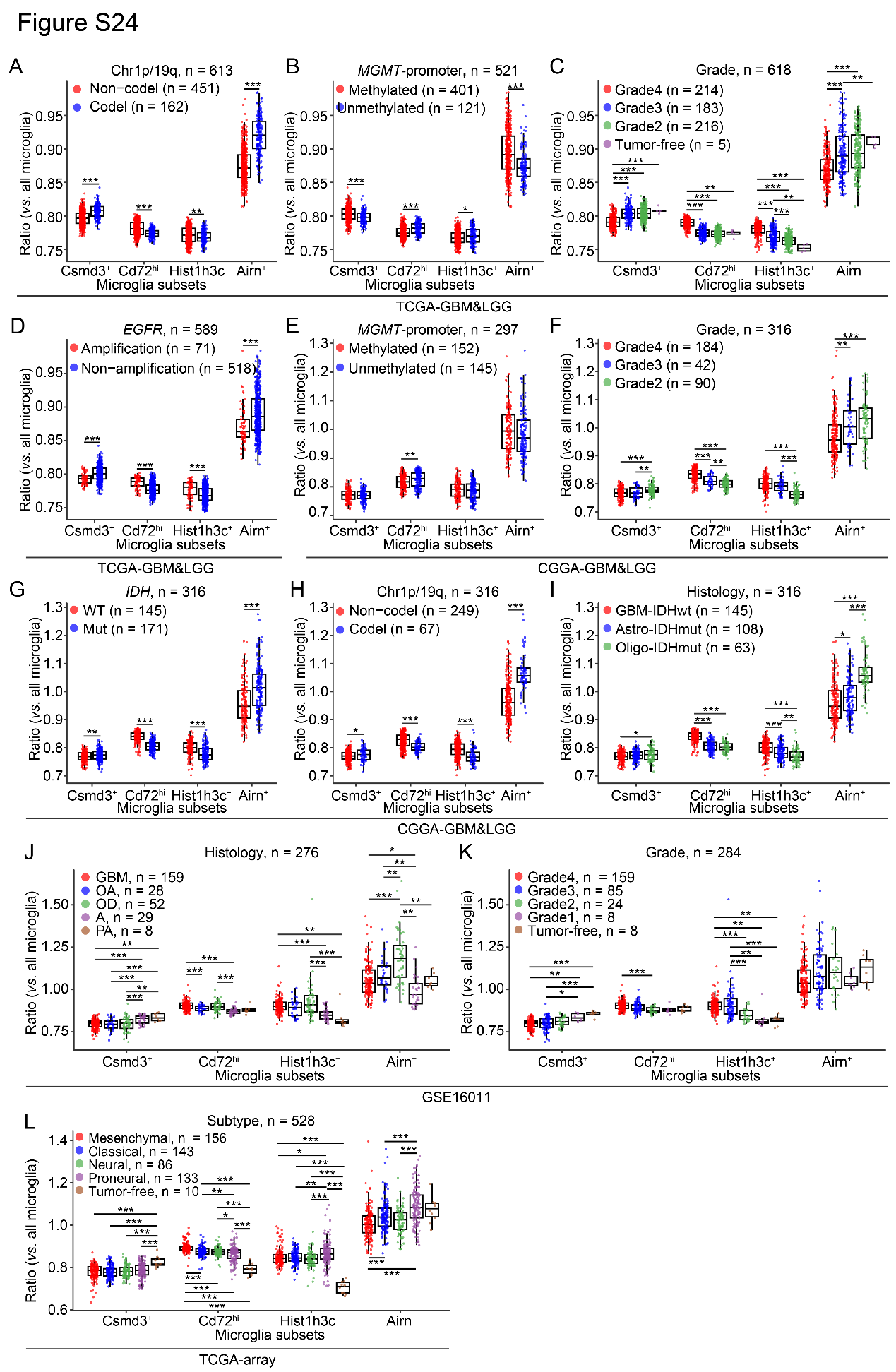


**Figure S26.** **Prognostic analysis of** **refined microglia subclusters identified from integrated scRNA-seq of ICu and control mice, related to Figure 4.**

(A-L) Boxplots depicting estimated proportions of microglia subclusters across diffuse gliomas by type, grade, or molecular signatures, analyzed across TCGA-GBM/LGG (A–D), CGGA-GBM/LGG (E–I), GSE16011 (J–K), and TCGA-array (L) cohorts. Center line shows median, box limits indicate upper and lower quartiles, and whiskers extend 1.5 times the interquartile range.

Statistical analysis, two-tailed unpaired Student’s t test (A, B, D, E, G and H), one-way ANOVA followed by Tukey’s post hoc test (C, F, I, J, K and L), Error bars, mean ± SEM. ^*^*p* < 0.05; ^**^*p* < 0.01; ^***^*p* < 0.001; ns, not significant.


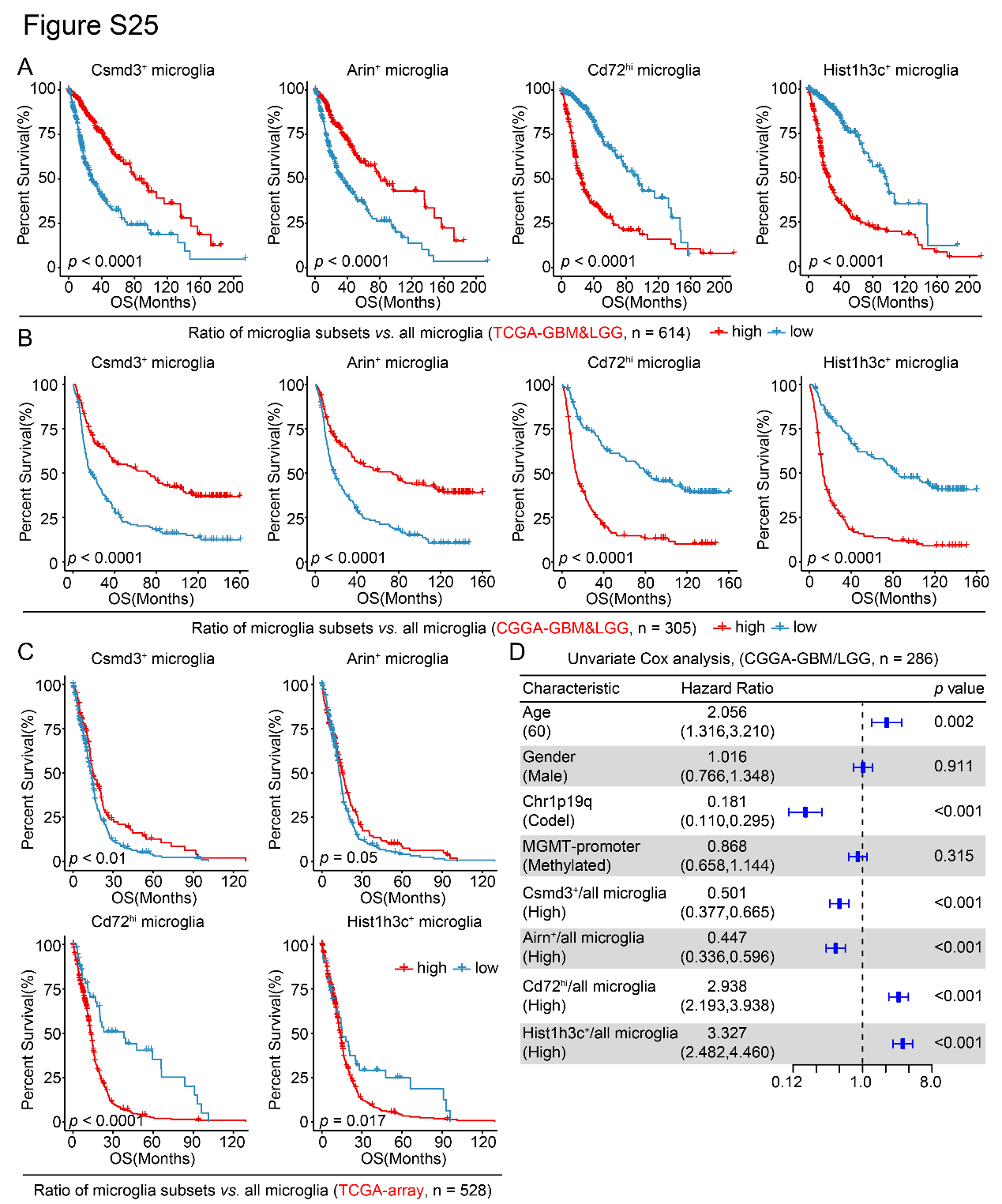


**Figure S27. Prognostic evaluation of refined microglia subclusters from integrated scRNA-seq of ICu and control mice using survival and Cox regression analyses, related to Figure 4.**

(A-C) Overall survival of glioma patients in TCGA (n=641) (A), CGGA (n=305) (B), and TCGA-array (n = 528) (C) cohorts, stratified by high vs. low median ratio of refined microglia subclusters to total microglia.

(D) Univariate Cox regression analysis of overall survival in CGGA patients (n = 286), stratified by high vs. low median ratio of refined microglia subclusters to total microglia. Forest plots with error bars show 2.5% (lower) and 97.5% (higher) bounds of the confidence interval.

Survival curves were analyzed using a log-rank (Mantel-Cox) test (A-C).


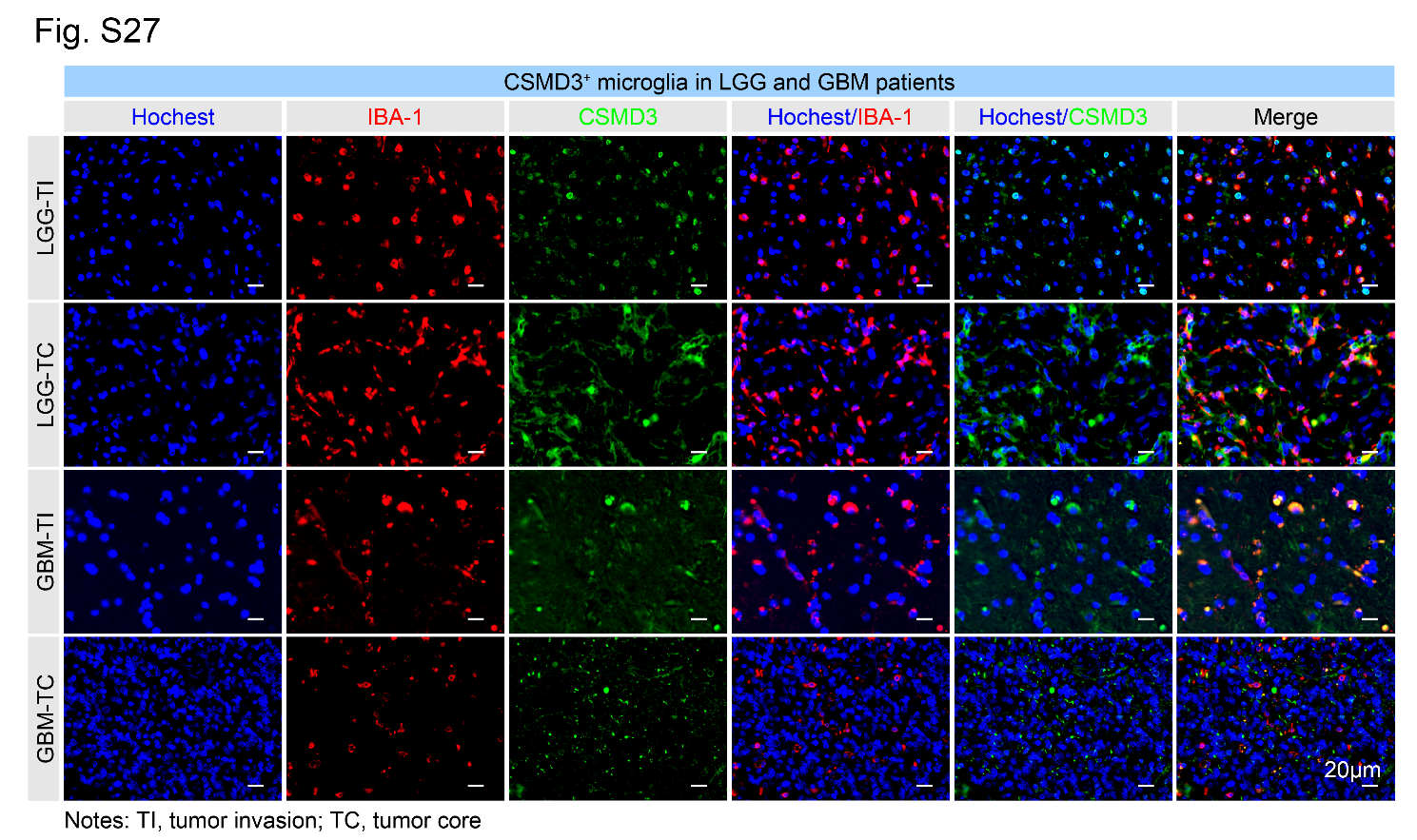


**Figure S28. Csmd3⁺ microglia in** **LGG and GBM patients, related to Figure 4.**

Representative IF images showing IBA-1 and CSMD3 with Hoechst staining in the TC and TI regions of LGG and GBM patients.


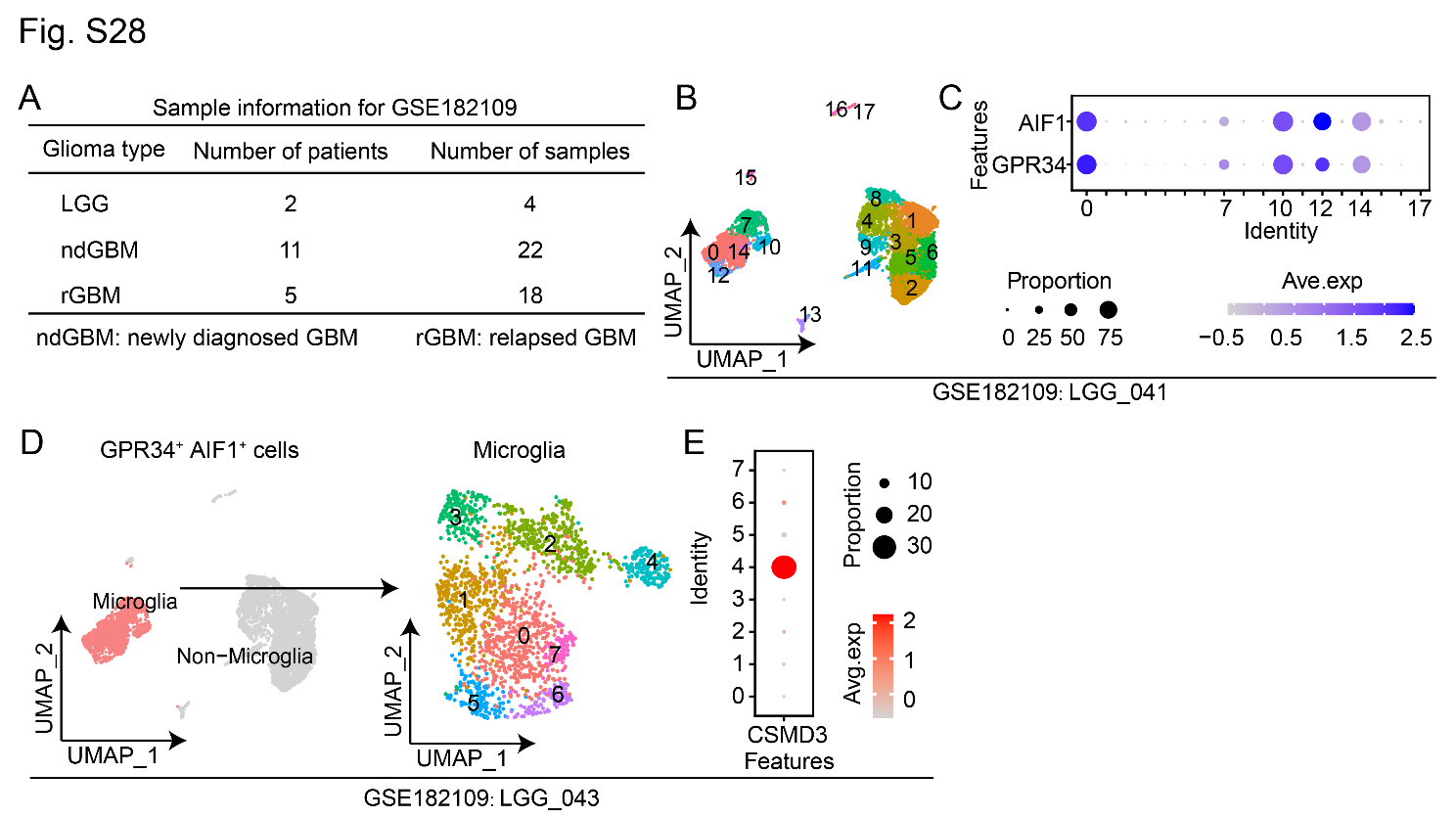


**Figure S29. Csmd3⁺ microglia in human glioma public scRNA-seq datasets, related to Figure 4.**

(A) Three-line table summarizing clinical characteristics of patients in the human glioma scRNA-seq dataset (GSE182109).

(B) UMAP visualization of all identified cell clusters in patient LGG_041.

(C) Dot plots depicting microglia marker gene expression (AIF1 and GPR34) across all cell clusters in patient LGG_041. Data are plotted as log-normalized counts, and the dot size represents the proportion of cells with the given gene detected. Avg.exp, average expression.

(D) UMAP plots depicting microglia (GPR34⁺AIF⁺) and non-microglia populations of LGG_041 patient (left panel), as well as microglial subclusters (right panel), based on public scRNA-seq data from the GEO dataset GSE182109.

(E) Dot plots showing the expression of CSMD3 in all microglia subclusters from LGG_041 patient. Data are plotted as log-normalized counts, and the dot size represents the proportion of cells with the given gene detected. Avg.exp, average expression.


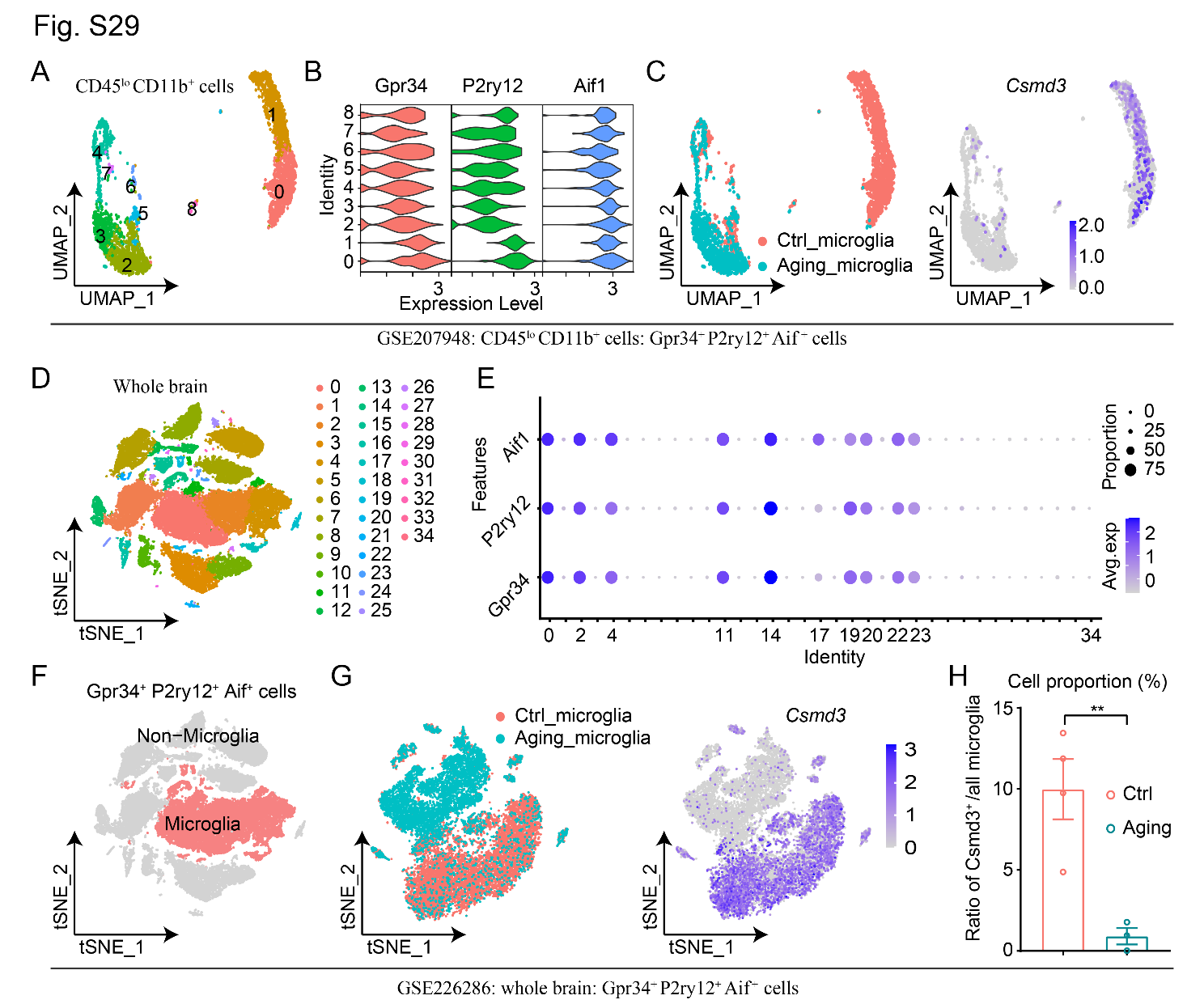


**Figure S30. Csmd3⁺ microglia in public mouse scRNA-seq datasets used to study microglia aging, related to Figure 4.**

(A) UMAP visualization of all cell clusters identified from flow-sorted microglia (CD45^lo^CD11b⁺) following scRNA-seq (GSE207948).

(B) Violin plots depicting the expression of microglia marker genes (Gpr34, Aif1, and P2ry12) across all subclusters in (A).

(C) UMAP visualization of aged versus control microglia after Harmony integration (left panel), highlighting Csmd3 expression predominantly in non-aged (control) microglia (right panel) (GSE207948).

(D) UMAP plot showing all cell clusters identified from mouse whole brain scRNA-seq (GSE226286).

(E) Dot plots depicting microglia marker gene expression (Gpr34, Aif1, and P2ry12) across all cell clusters in (D). Data are plotted as log-normalized counts, and the dot size represents the proportion of cells with the given gene detected. Avg.exp, average expression.

(F) UMAP plot showing microglia (Gpr34^+^Aif1^+^P2ry12^+^) identified from mouse whole brain scRNA-seq (GSE226286).

(G) UMAP plots of aged and control microglia after Harmony integration (left panel), with Csmd3 expression mainly restricted to non-aged (control) microglia (right panel) (GSE226286).

(H) Statistical analysis of the proportion of Csmd3⁺ microglia among all microglia during microglial aging, based on public scRNA-seq data from the GEO datasets GSE207948 and GSE226286.

Statistical analysis, two-tailed unpaired Student’s t test (H). Error bars, mean ± SEM. ^**^*p* < 0.01.


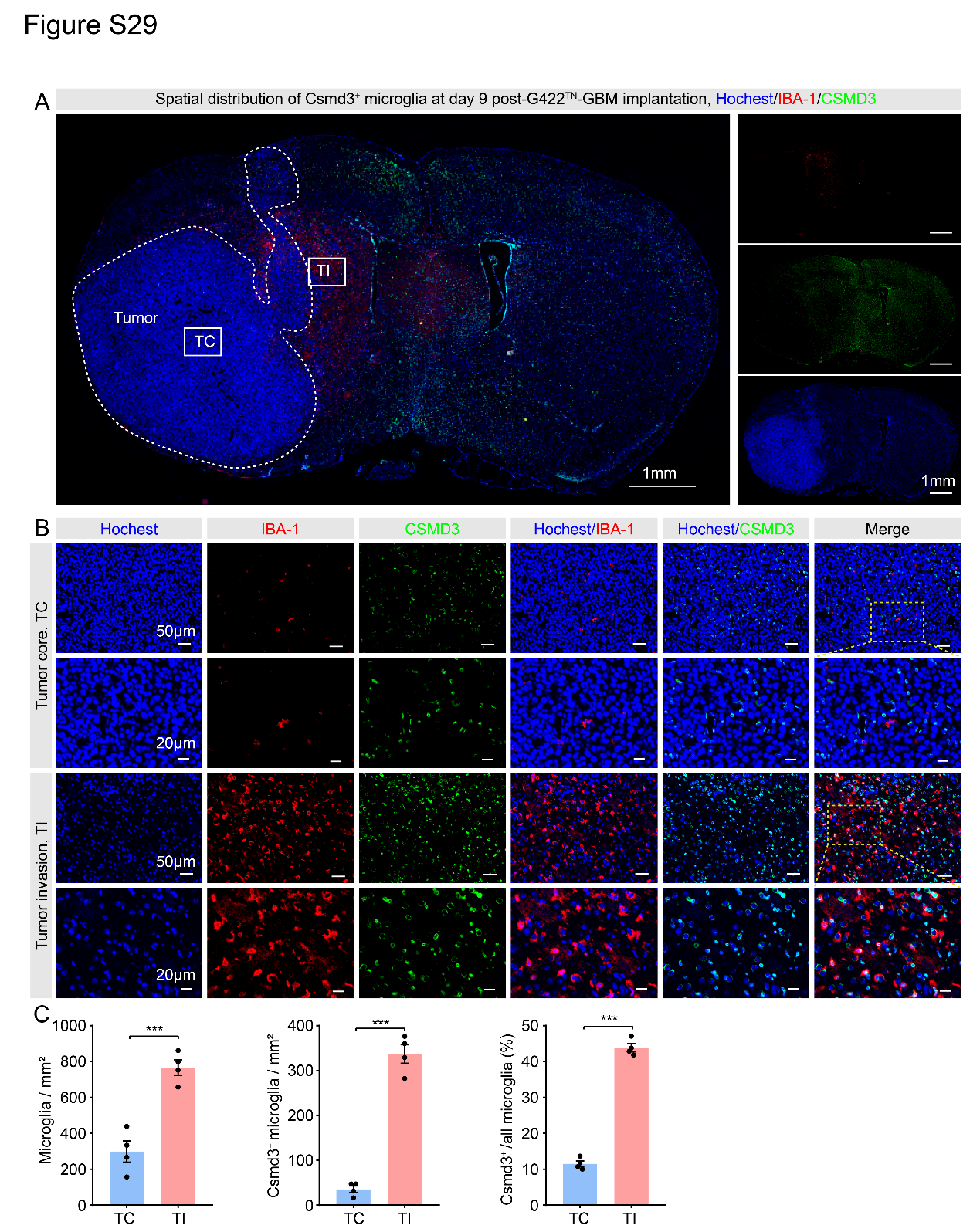


**Figure S31. Csmd3⁺ microglia in day 9 G422^TN^-GBM tumors, related to Figure 5.**

(A) Representative IF images of IBA1 and CSMD3 staining in the whole brain of G422^TN^-GBM mice.

(B) Representative IF images of IBA1 and CSMD3 staining in the TC and TI region of G422^TN^-GBM mice. TI, tumor invasion; TC, tumor score.

(C) Quantitative analysis of IF images showing microglia (IBA-1⁺) (left panel), Csmd3⁺ microglia (IBA-1⁺CSMD3⁺) (median panel), and the proportion of Csmd3⁺ microglia among all microglia (right panel) in G422^TN^-GBM tumors on day 9 *p.i.* across TC and TI region (n = 4/group).

Statistical analysis, two-tailed unpaired Student’s t test (C). Error bars, mean ± SEM. ^***^*p* < 0.001.


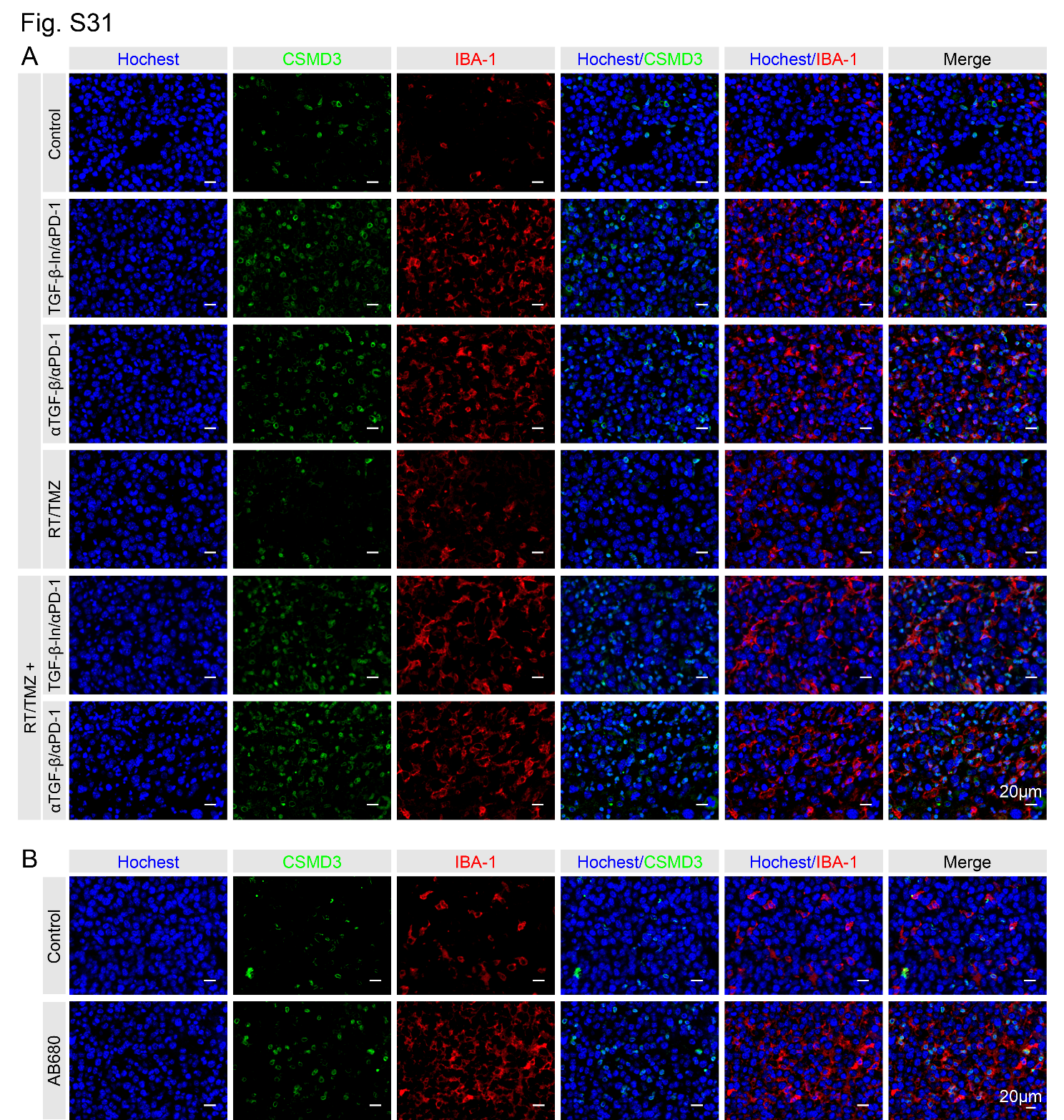


**Figure S32. Treatment-dependent distribution of Csmd3⁺ microglia in G422^TN^-GBM mice, related to Figure 5.**

(A) Representative IF images of IBA1 and CSMD3 staining in the TC region of the G422^TN^-GBM mice across TβRI-In/αPD-1, αTGF-β/αPD-1, RT/RMZ, RT/TMZ/TβRI-In/αPD-1, RT/TMZ/αTGF-β/αPD-1 treatment and control group on day 9 *p.i.*.

(B) Representative IF images of IBA1 and CSMD3 staining in the TC region of the G422^TN^-GBM mice from the AB680-treated and control groups on day 7 *p.i.*.


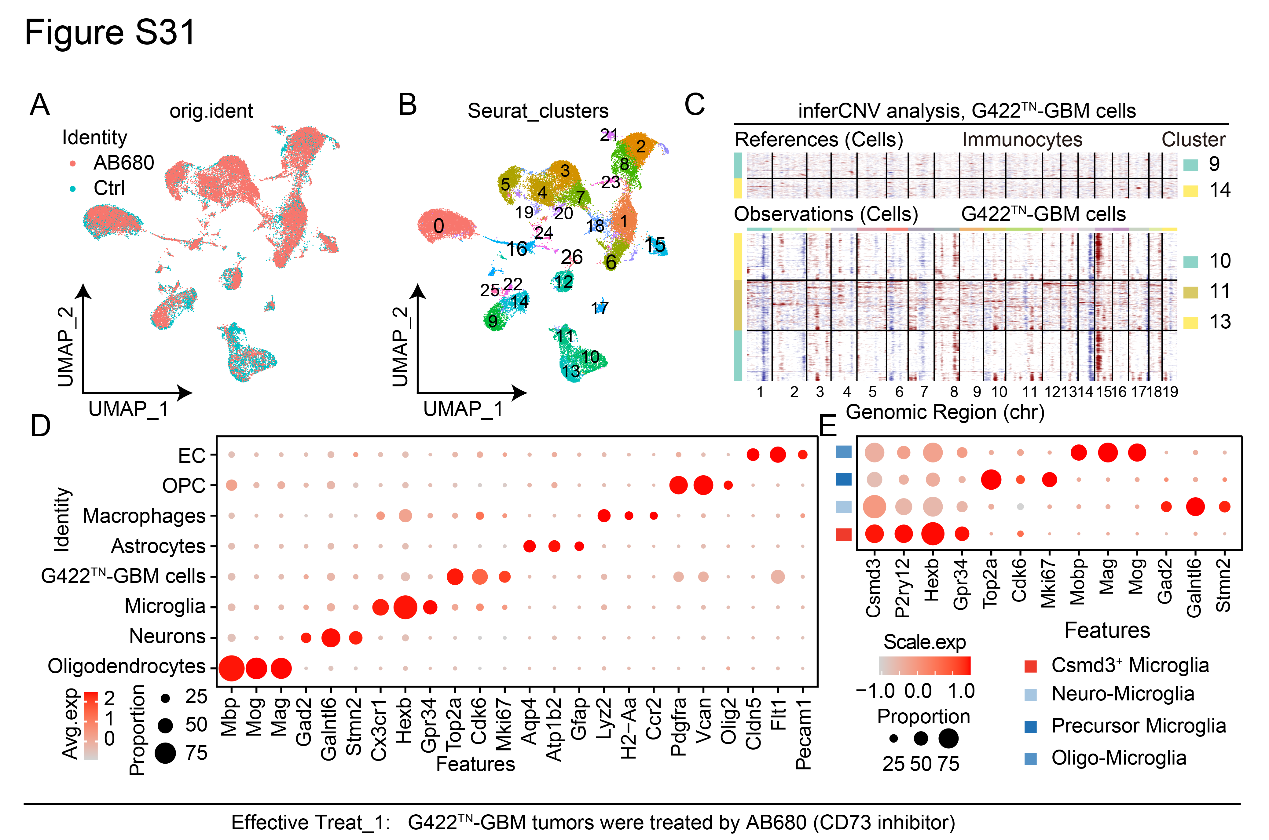


**Figure S33. Integrated snRNA-seq analysis of** **AB680-treated and control G422^TN^-GBM mice, related to Figure 5.**

(A) UMAP plot depicting the integration of snRNA-seq data from AB680-treated and control mice using the Harmony algorithm. AB680, CD73 inhibitor.

(B) UMAP plot showing all cell clusters in the integration of snRNA-seq data from AB680-treated and control mice.

(C) Hierarchical heatmap showing large-scale CNV profile of each G422^TN^-GBM cell cluster (10, 11, 13). Red and blue colors represent high and low CNV level, respectively. Macrophages (cluster 14) and microglia (cluster 9) are defined as reference cells.

(D) Dot plots showing the expression of specific marker genes across all cell types in the integration of snRNA-seq data from AB680-treated and control mice. Data are plotted as log-normalized counts, and the dot size represents the proportion of cells with the given gene detected. Avg.exp, average expression.

(E) Heatmap and dot plots showing the expression of marker genes across four microglial subtypes in the integrated snRNA-seq data from AB680-treated and control mice. Data are plotted as log-normalized counts, and the dot size represents the proportion of cells with the given gene detected. Scale.exp, scaled expression.


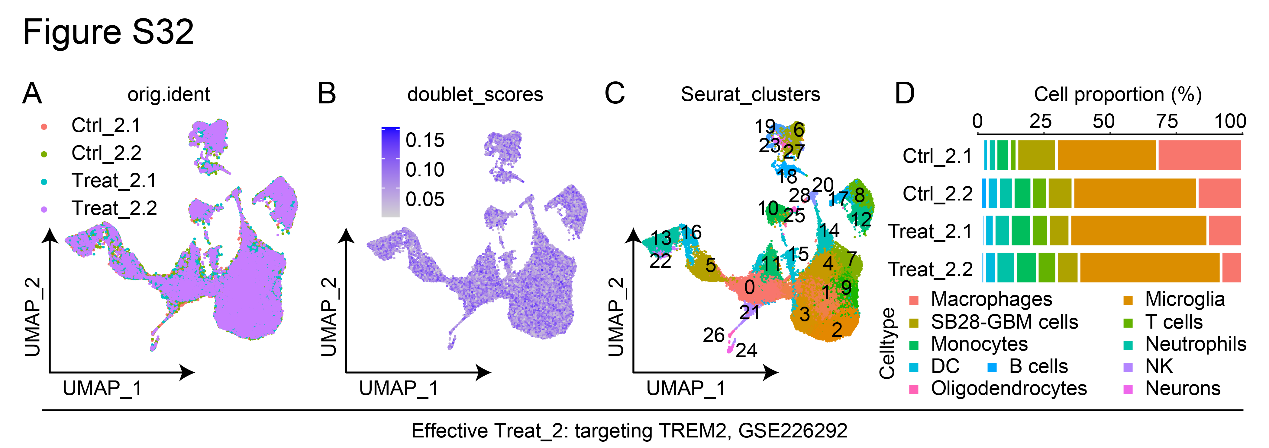


**Figure S34. Integrated scRNA-seq analysis of ASO-treated and control SB28-GBM mice, related to Figure 5.**

(A) UMAP plot depicting the integration of scRNA-seq data from ASO-treated and control mice using the Harmony algorithm. ASO, antisense oligonucleotides to target Trem2.

(B) UMAP plot showing doublet scores of the integrated snRNA-seq data from ASO-treated and control mice, calculated using Scrublet (Python package).

(C) UMAP plot showing all cell clusters in the integration of snRNA-seq data from ASO-treated and control mice.

(D) Stacked barplot of cell composition of ASO-treated and control mice from public scRNA-seq dataset (GSE226292).


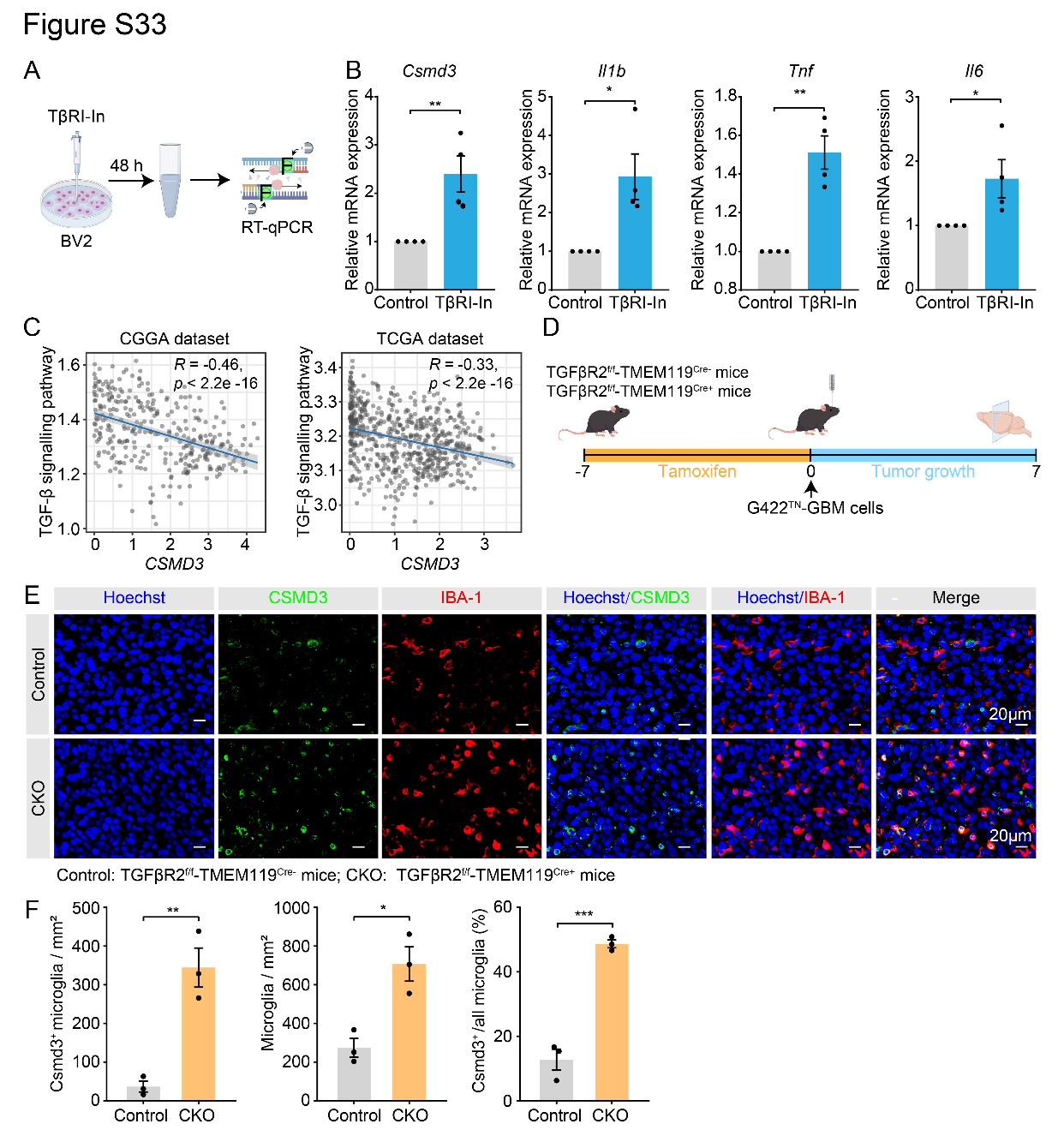


**Figure S35.** **Blocking** **TGF-β signaling in microglia *in vivo* and *in vitro* to examine microglial phenotypic changes and tumor infiltration, related to Figure 6.**

(A) Schematic illustration of RT-qPCR experiments performed 48 h after blocking TGF-β signaling in BV2 microglial cells *in vitro* (by Figdraw).

(B) RT-qPCR analysis showing elevated *Csmd3*, *Il1b*, *Tnf* and *Il6* mRNA levels in BV2 cells treated with TβRI-In compared to controls (n = 4/group).

(C) Correlation plots showing the association between CSMD3 expression and TGF-β signaling pathway scores in glioma samples from the Chinese Glioma Genome Atlas (CGGA) (left panel) and The Cancer Genome Atlas (TCGA) (right panel) datasets.

(D) Schematic illustration of sample collection on day 7 *p.i.* in the G422^TN^-GBM model established using TMEM119-CreER^T2^; Tgfbr2^flox/flox^ conditional knockout (CKO) mice (by Figdraw).

(E-F) Representative IF images (E) showing IBA1 and CSMD3 staining, and quantitative analysis (F) of microglia (IBA1⁺, left panel), Csmd3⁺ microglia (IBA1⁺CSMD3⁺, middle panel), and the proportion of Csmd3⁺ microglia among total microglia (right panel) in G422^TN^-GBM tumors on day 7 p.i. across CKO and control groups (n = 3/group).

Statistical analysis, two-tailed unpaired Student’s t test (B and F). Error bars, mean ± SEM. ^*^*p* < 0.05; ^**^*p* < 0.01; ^***^*p* < 0.001.


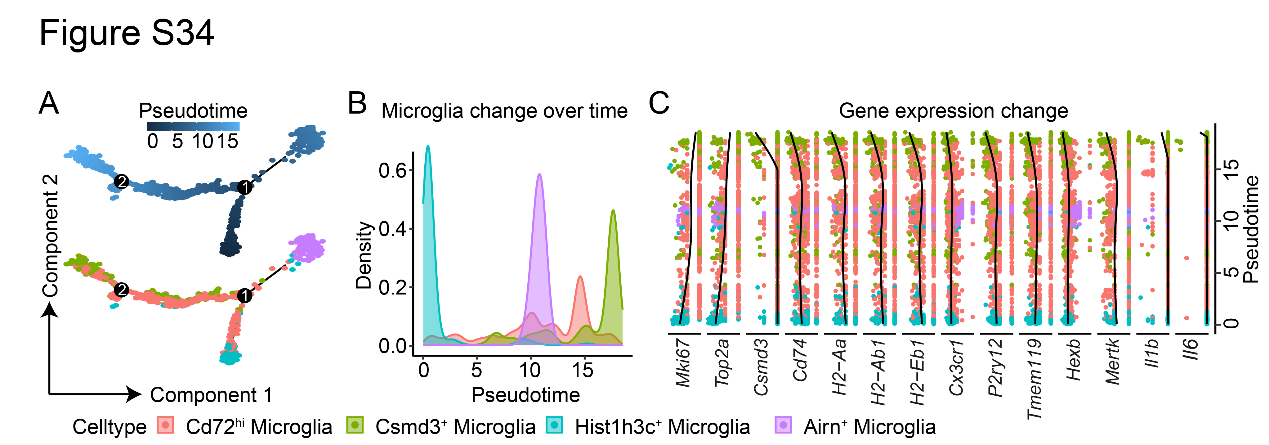


**Figure S36. Pseudotime analysis of microglia cell subsets in ICu and control mice, related to Figure 6.**

(A) Trajectory plots of microglia ordered along pseudotime (upper panel), highlighting differentiation dynamics across subtypes (lower panel).

(B) Density analysis highlights subtype-specific progression along the trajectory.

(C) Scatter plots show the expression levels of selected genes, including proliferative markers (*Mki67*, *Top2a*), immune activation genes (*Cd74*, *H2-Aa*, *H2-Ab1*, *H2-Eb1*), homeostatic markers (*Cx3cr1*, *P2ry12*, *Tmem119*, *Hexb*), and inflammatory mediators (*Mertk*, *Il1b*, *Il6*), ordered by pseudotime. Each dot represents a single cell, and the black curves indicate the smoothed expression trend. The results reveal a dynamic transition from proliferative to activated and inflammatory microglial states during disease progression.


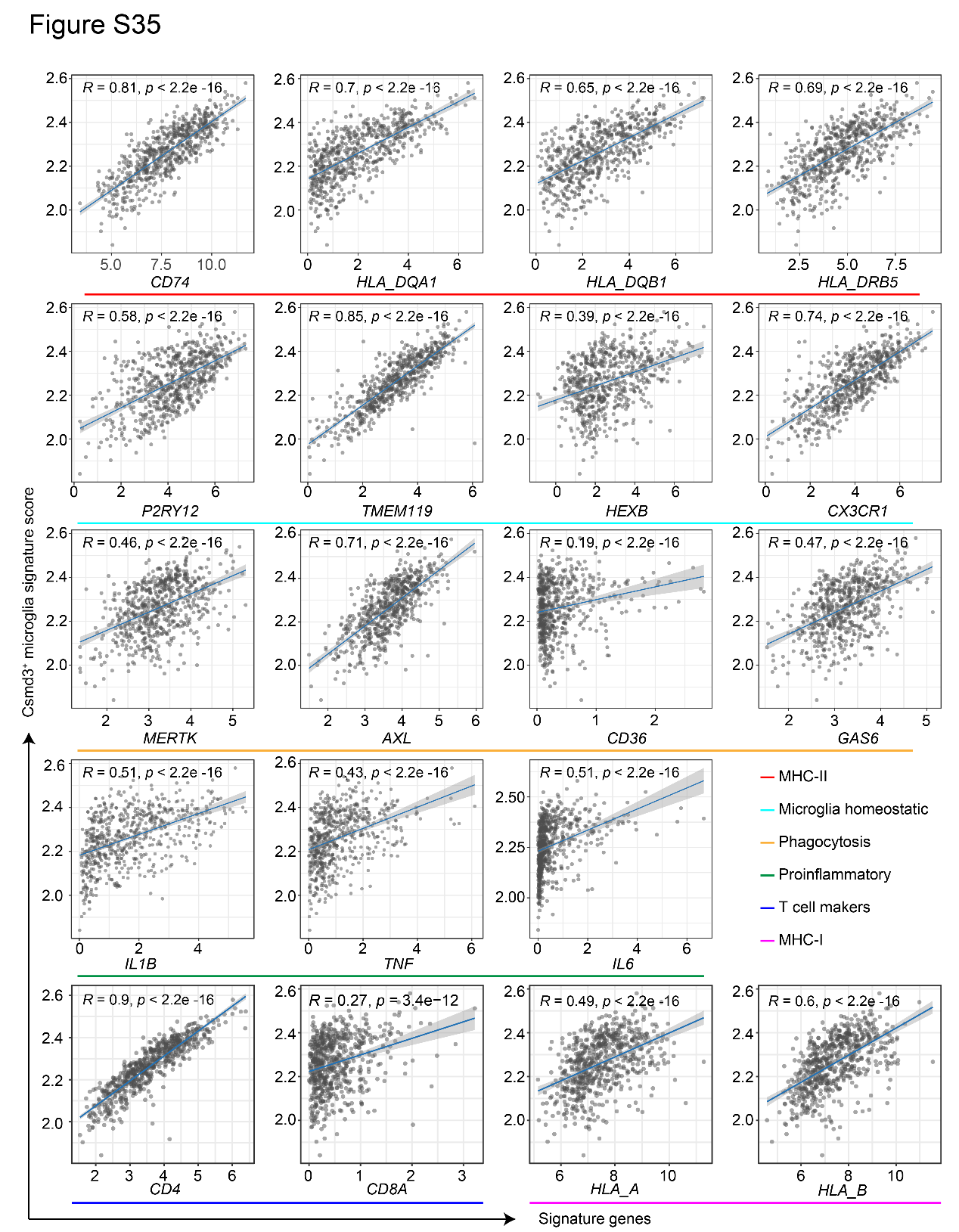


**Figure S37. Correlation analysis between Csmd3⁺ microglia and genes related to MHC-II, microglial homeostasis, phagocytosis, proinflammation, MHC-I, and T cell markers, related to Figure 6.**

Correlation plots showing the relationship between Csmd3⁺ microglia signature scores and MHC-II signature genes (*CD74*, *HLA-DQA1*, *HLA-DQB1*, *HLA-DRB5*), microglial homeostasis genes (*P2RY12*, *TMEM119*, *HEXB*, *CX3CR1*), phagocytosis genes (*MERTK*, *AXL*, *CD36*, *GAS6*), pro-inflammatory genes (*IL1B*, *TNF*, *IL6*), MHC-I genes (*HLA-A*, *HLA-B*), and T cell marker genes (*CD4*, *CD8A*) in glioma samples from The Cancer Genome Atlas (TCGA, n=614).


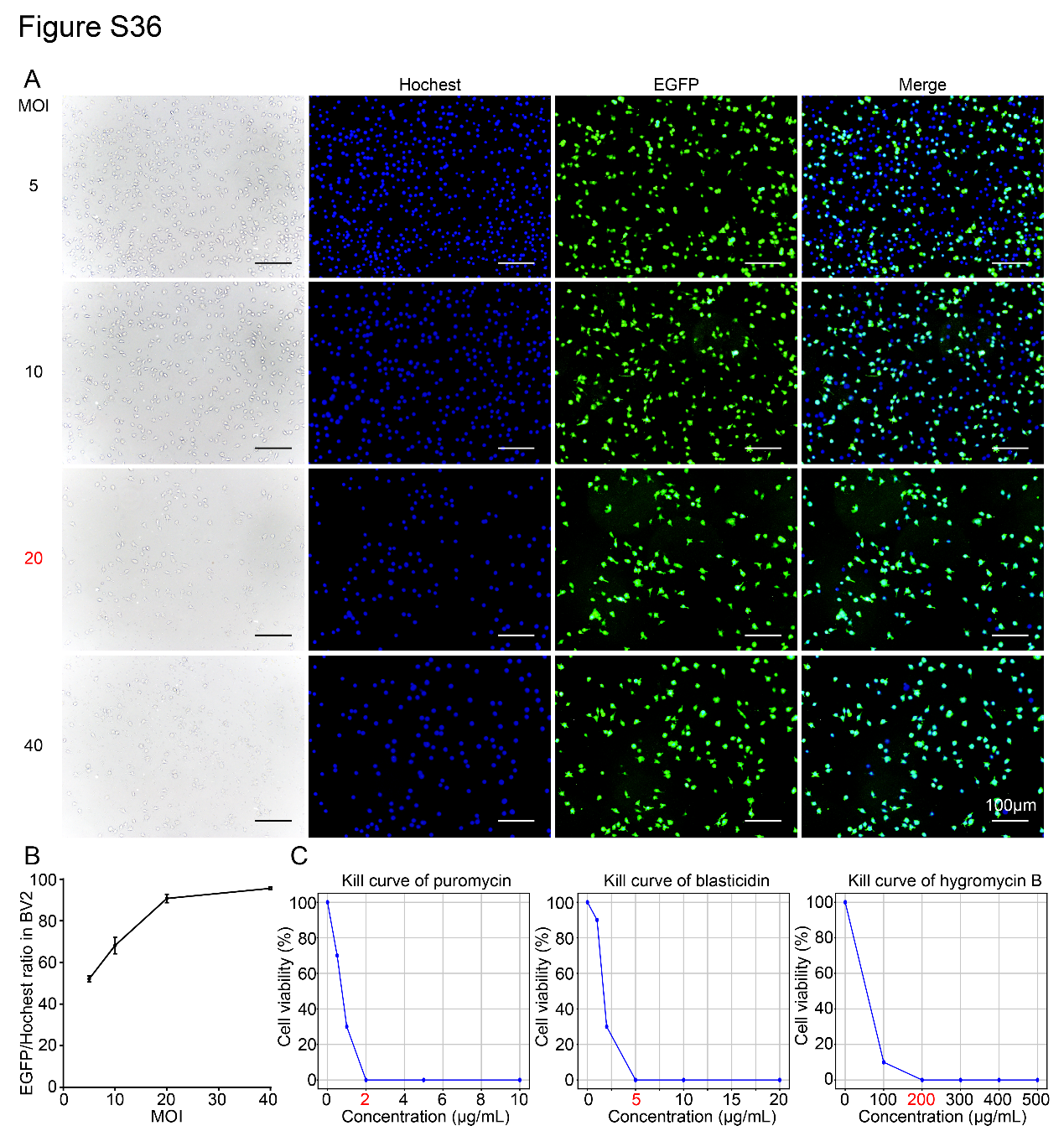


**Figure S38. Determination of the optimal MOI for BV2 cells and the minimal lethal concentrations of puromycin, blasticidin, and hygromycin B in pilot experiments, related to Figure 6.**

(A) Representative IF and bright-field images of BV2 cells at varying MOI values. MOI, multiplicity of infection.

(B) Quantitative analysis of viral infection efficiency in BV2 cells (EGFP/Hoechst) across different MOI values to determine the optimal MOI.

(C) Cell viability assays to determine the minimal lethal concentrations of puromycin, blasticidin, and hygromycin B in BV2 cells.


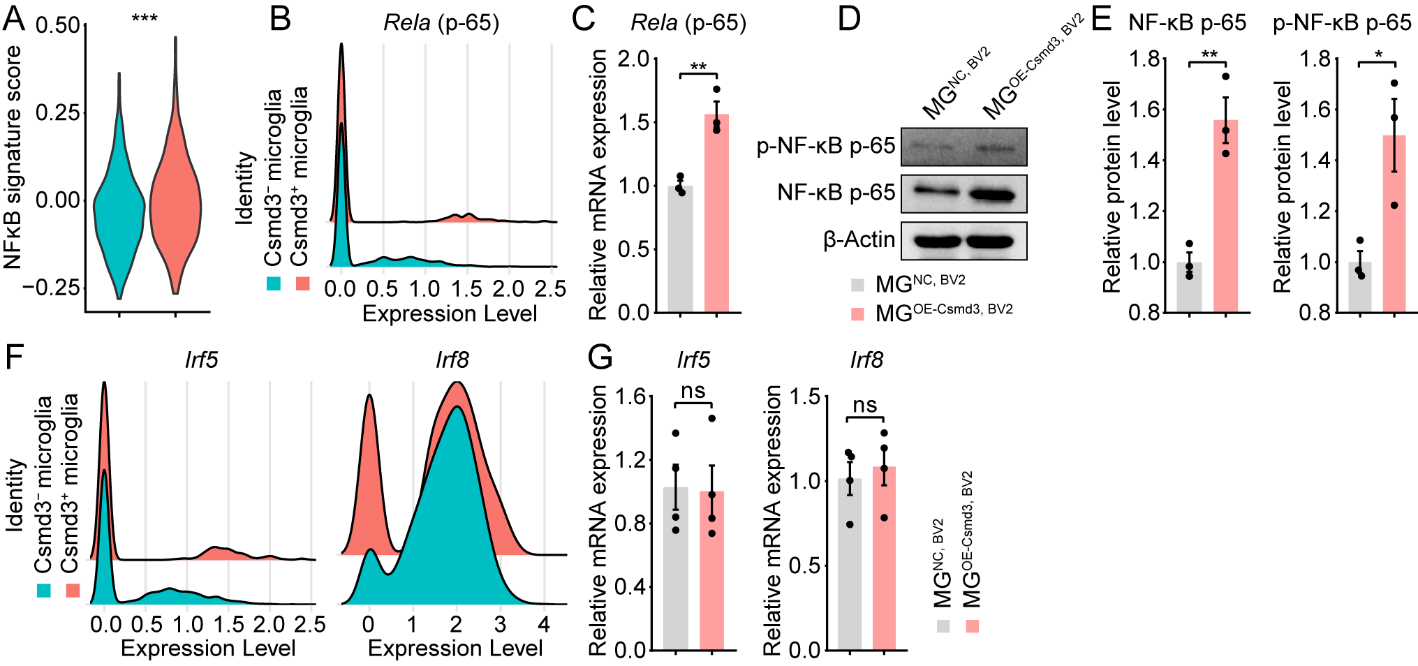


**Figure S39.** **Csmd3 membrane protein promotes pro-inflammatory activation of microglia, related to Figure 6.**

(A) Violin plots showing NF-κB signaling signature scores in Csmd3⁺ and Csmd3⁻ microglia.

(B) Ridge plots showing the expression of *Rela* (encoding p-65) in Csmd3⁺ and Csmd3⁻ microglia.

(C) RT-qPCR analysis showing *Rela* mRNA levels in MG^OE-Csmd3, BV2^ and MG^NC, BV2^ cells (n = 3/group).

(D-E) Representative Western blotting results (D) and statistical analysis (E) of NF-κB p-65 protein and p-NF-κB p65 in MG^OE-Csmd3, BV2^ and MG^NC, BV2^ cells (n = 3/group).

(F) Ridge plots showing the expression of *Irf5* and *Irf8* in Csmd3⁺ and Csmd3⁻ microglia.

(G) RT-qPCR analysis showing *Irf5* and *Irf8* mRNA levels in MG^OE-Csmd3, BV2^ and MG^NC, BV2^ cells (n = 4/group).

Statistical analysis, two-tailed unpaired Student’s t test (A, E, G). ∗*p* < 0.05; ∗∗*p* < 0.01; ns, no significance.


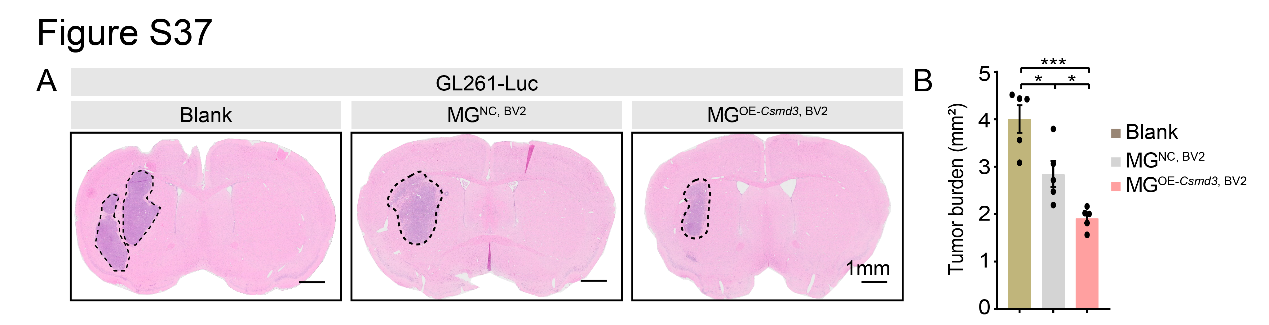


**Figure S40. Csmd3⁺ microglia markedly inhibit GBM progression in the GL261-GBM mouse model, related to Figure 6.**

(A-B) Representative H&E staining (A) and quantitative analysis (B) of GL261-GBM tumor burden on day 7 *p.i.* in the Blank group (GL261-GBM alone), GL261-GBM + MG^NC, BV2^, and GL261-GBM + MG^OE-^*^Csmd3^*^, BV2^ groups (n = 5/group). GL261-Luc, GL261-GBM cell overexpressing luciferase.

Statistical analysis, one-way ANOVA followed by Tukey’s post hoc test (B) Error bars, mean ± SEM. ^∗^*p* < 0.05; ^∗∗∗^*p* < 0.001.


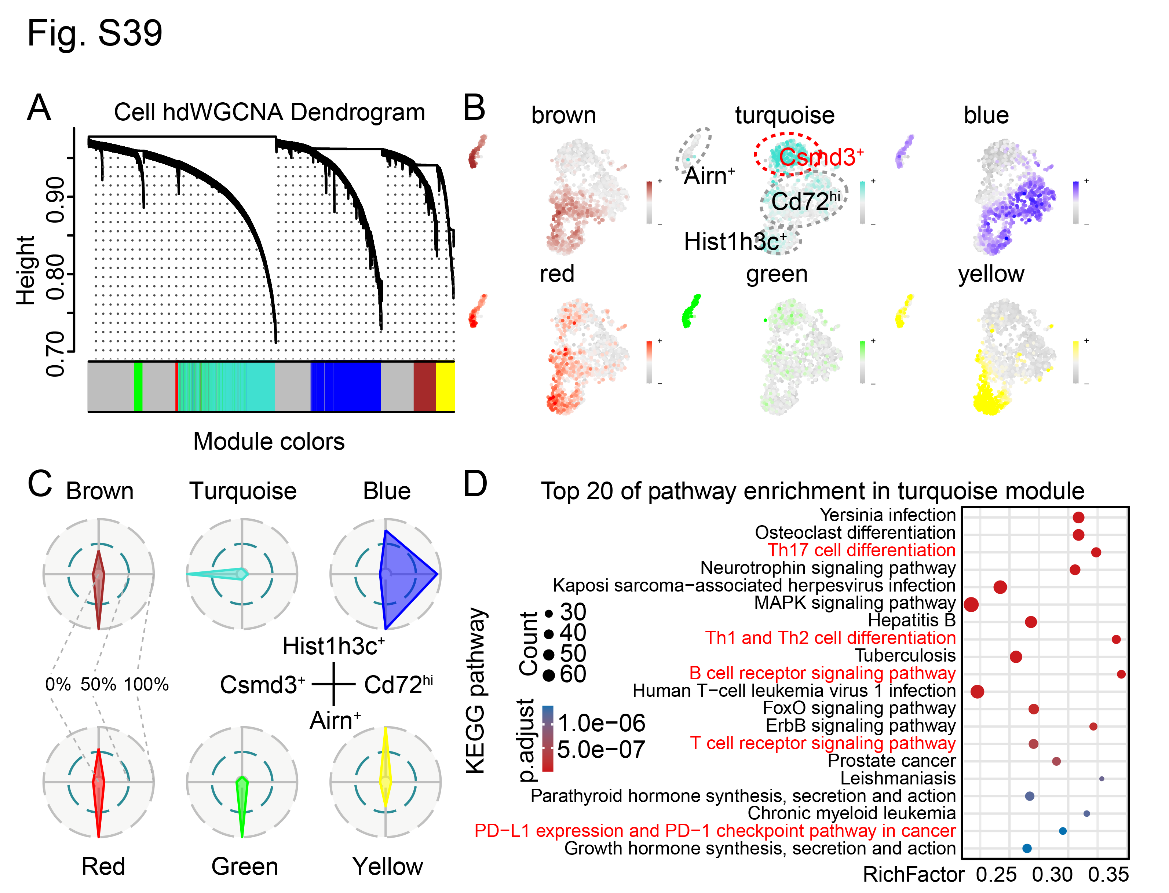


**Figure S41.** **HdWGCNA analysis of microglia from integrated scRNA-seq data of ICu and control mice, related to Figure 7.**

(A) Dendrogram from the hdWGCNA analysis conducted on microglia gene expression profiles, with colors indicating various gene modules. hdWGCNA, high dimensional weighted gene co-expression network analysis

(B) Feature plots showing harmonized module expression (hME) of hdWGCNA gene modules in microglia. hME, Module Eigengenes

(C) Radar plot visualizing the harmonized module expression (hME) of hdWGCNA gene modules across different microglial cell types. Each colored polygon corresponds to a specific gene module, illustrating its relative activity pattern among the various microglial cell types.

(D) Top 20 enrichment pathways of the turquoise module genes through KEGG pathway enrichment analysis.


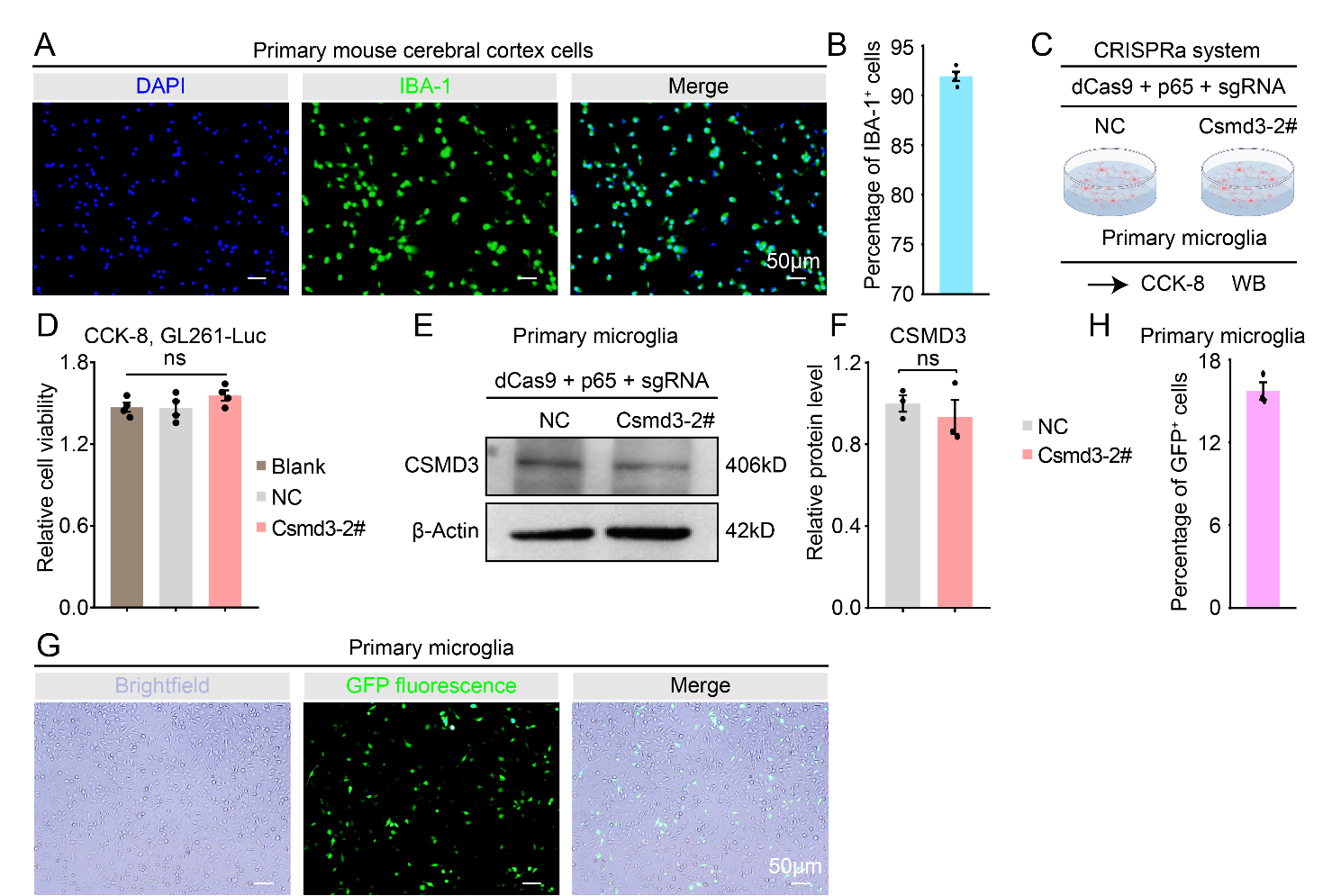


**Figure S42. Isolation of primary mouse microglia and subsequent viral infection-related experiments.**

(A-B) Representative immunofluorescence images of IBA-1 staining in primary mouse microglia after isolation (A) and quantification of the proportion of IBA-1^+^ cells (B) (n=4).

(C) Schematic illustration of simultaneous infection of primary microglia with the three lentiviral vectors required for the CRISPRa system and the subsequent experimental workflow.

(D) Statistical analysis of relative cell viability (CCK-8 assay) of GL261-GBM cells after 48 h stimulation with conditioned medium from virus-infected primary microglia (n=4/group).

(E-F) Representative Western blot images (E) and quantitative analysis (F) of CSMD3 protein expression in primary microglia following viral infection (n=3/group), with β-Actin used as a loading control. Simultaneous infection failed to upregulate CSMD3 expression.

(G-H) Representative GFP fluorescence images showing successful simultaneous infection with the three CRISPRa lentiviral vectors (G) and quantification of the proportion of GFP+ cells (H) (n = 3). Error bars, mean ± SEM. ns, not significant.

**Table S1.** Tumor microenvironment subtype classification and characteristics in GBM and other cancer types, related to Figures 1 and S9.

**Table S2.** Molecular mutation characteristics of the G422^TN^-GBM model, related to Figure 1.

**Table S3.** Signature gene sets used for signaling pathway analyses in this study, related to Figures 2, 3, 7, S10, S11 and S17. (A-B) Human TGF-β (A) and TLS (B) signaling signature gene sets. (C-G) Mouse TGF-β (C), IL1 (D), TNF (E), IL6 (F) and NFKB (G) signaling signature gene sets.

**Table S4.** Literature statistics on studies related to tumor rechallenge and antitumor immune memory in glioma, related to Figure S19.

**Table S5.** Differentially expressed genes (DEGs) defining Csmd3⁺ microglia relative to other microglial subsets, and between Csmd3⁺ microglia from ICu and control mice, related to Figures 6 and S23. (A) Csmd3⁺ microglia vs other microglia. (B) Csmd3⁺ microglia in ICu vs control mice.

**Table S6.** Clinical information of glioma patient samples used in this study, related to Figure 4.

**Table S7.** Signature gene sets of four identified microglial subclusters and total microglia identified from the integrated scRNA-seq data of ICu and control mice, related to Figures 4, 5, 7, S26, S27 and S37. (A) Csmd3⁺ microglia. (B) Cd72^hi^ microglia. (C) Hist1h3c^+^ microglia. (D) Airn^+^ microglia. (E) total microglia.

**Table S8.** Primer sequences used for RT-qPCR experiments in this study, related to Figures 2, 6, 7, S15, S35 and S39.
