# Supplementary material for: Identifying a Csmd3+ Microglial Subpopulation that Drives Cold‐to‐Hot Transition and Immune‐Cure in Glioblastoma
Source: Adv Sci (Weinh). 2026 Jul 20:e76690. Online ahead of print. doi: 10.1002/advs.76690 (PMC13383698; doi:10.1002/advs.76690)

**Raw Western blot images for**

**Identifying and targeting Csmd3^+^ microglia with** **immune-cure potential in a TME^Med^ glioblastoma mouse model**

Hai-Feng Jiang^1^, Pan-Pan Gao^1^, Yu-Wen Du^1^, Li-Qin Wu^2^, En-Zhi Yin^3^, Qi An^1^, Ze-Hua Ding^1^, Jin-Wen Shi^1^, Ya Shu^2^, Ruoqiao Chen^4^ *, Feng Liu^2^ *, Mingfeng Li^5,6,7^ *, Xiao Qian Chen^1^ *

**Raw data of Western blotting for Figure 6E**


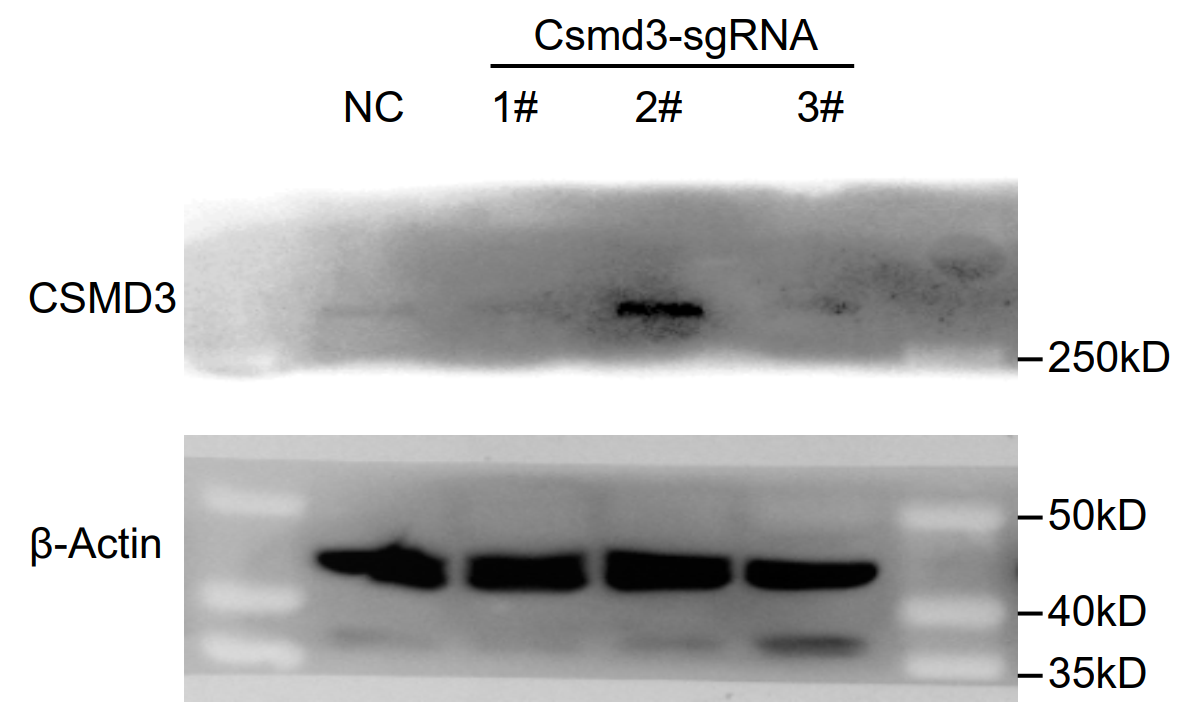


**Raw data of Western blotting for Figure S21D**


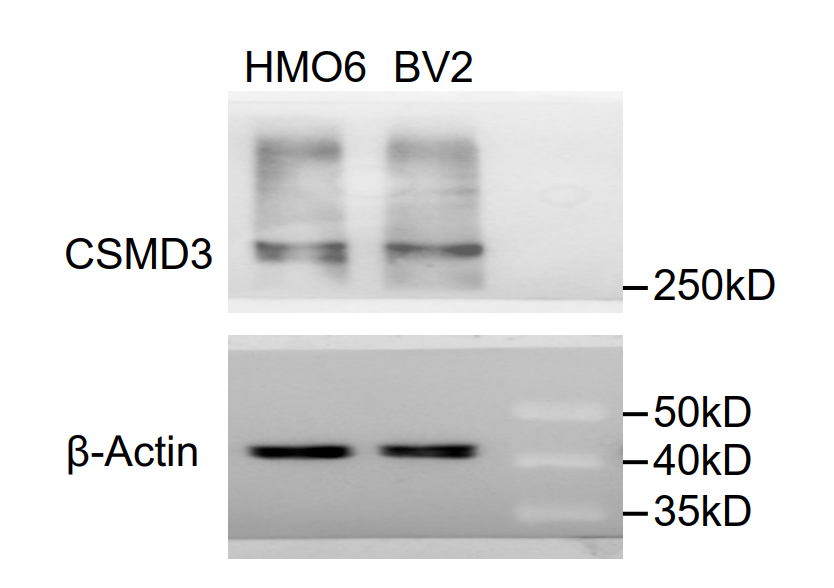


**Raw data of Western blotting for Figure S39D**


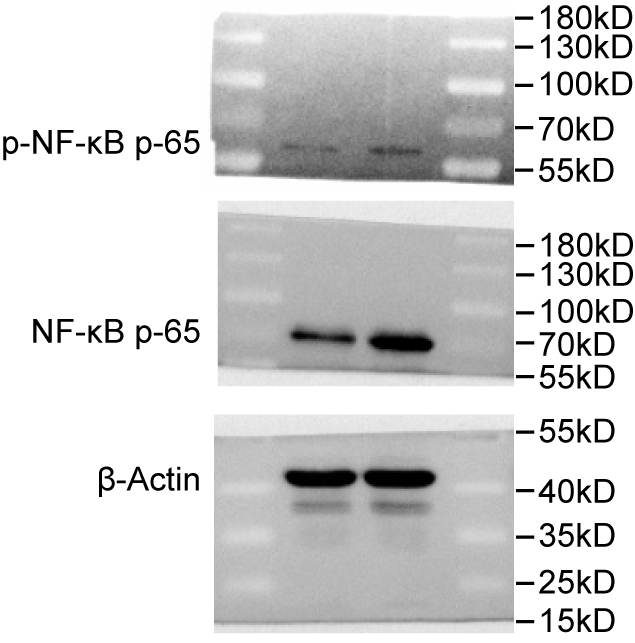


**Raw data of Western blotting for Figure S42E**


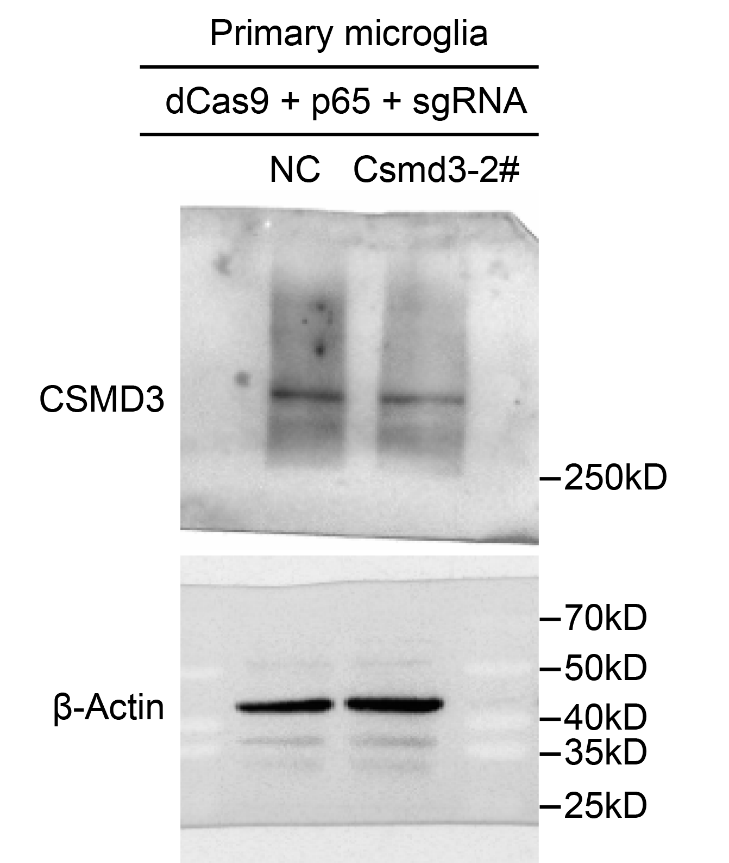

Supplement: Supplementary file 3 — Supporting File 3: advs76690‐sup‐0003‐Raw_Western_blot_images‐260606.docx. [file ADVS-9999-e76690-s003.docx]
